# CSNK1D inhibition suppresses head and neck squamous cell carcinoma progression through SHH and PTCH1 pathway

**DOI:** 10.1038/s41419-025-08276-7

**Published:** 2025-12-06

**Authors:** Li Yang, Xiang Li, Yun Zhu, Yu Zhang, Zheqi Liu, Chengzhong Lin, Canhua Jiang, Yu-Xiong Su, Tong Ji, Yang Wang, Jun Gao, Jinhai Ye

**Affiliations:** 1 Department of Oral and Maxillofacial Surgery, Zhongshan Hospital, Fudan University, Shanghai, China; 2 Department of Oral and Maxillofacial Surgery, Xiangya Hospital, Central South University, Changsha, China; 3 Department of Oral and Maxillofacial Surgery, The Affiliated Stomatological Hospital of Nanjing Medical University, Nanjing, China; 4 Shanghai Key Laboratory of Craniomaxillofacial Development and Diseases, Shanghai Stomatological Hospital & School of Stomatology, Fudan University, Shanghai, China; 5 Division of Oral and Maxillofacial Surgery, Faculty of Dentistry, The University of Hong Kong, Hong Kong SAR, China

**Keywords:** Oral cancer, Cancer microenvironment

## Abstract

Casein kinase 1δ (CK1δ, encoded by *casein kinase 1 delta*) (CSNK1D) is a serine/threonine protein kinase closely associated with cancer development. However, the biological functions of CSNK1D in head and neck squamous cell carcinoma (HNSCC) remain unclear. This study identifies CSNK1D as a previously unrecognized yet crucial regulator in the progression of HNSCC. CSNK1D expression levels were upregulated in tumor tissues and the up-regulation was positively correlated with poor survival in patients with HNSCC. Moreover, CSNK1D selectively bound SHH and PTCH1, and regulated the stability of the CSNK1D-SHH-PTCH1 complex to control the GLI1-BCL2 axis, leading to the downstream activation of the hedgehog pathway. SB-203580 could target and inhibit CSNK1D, offering new perspectives for the clinical treatment of HNSCC. Overall, CSNK1D is underscored as a vital oncogenic driver and promising therapeutic target for HNSCC.

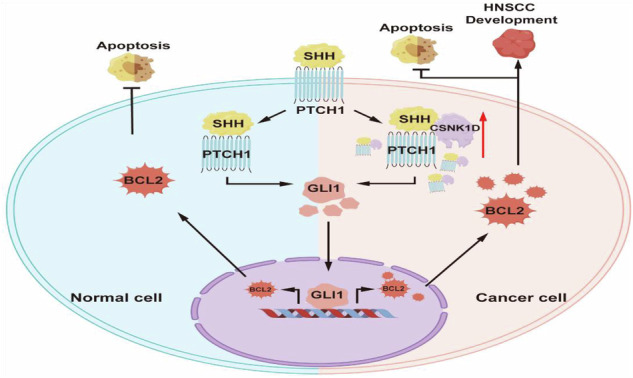

## Abstract (web summary)

Schematic illustration showing the suggested mechanism by which CSNK1D functions as an oncogene for HNSCC apoptosis through the hedgehog pathway. CSNK1D contributes to stable SHH and PTCH1 complexes to promote the nucleus entry of GLI1 to regulate BCL2 transcription.

## Introduction

Head and neck squamous cell carcinoma (HNSCC), the most common subtype of head and neck malignancy, arises from the oral cavity, pharynx and larynx. HNSCC ranks sixth among the most common cancers worldwide, with 890,000 new cases and 450,000 deaths reported in 2018 [1, 2]. Although HNSCC is a multi-stage and multi-process disease, most patients present with advanced-stage HNSCC upon diagnosis. Despite recent therapeutic advances in HNSCC treatment, the 5-year survival rate has only seen modest improvement [3]. Moreover, the complications after the treatment seriously affect the patients’ facial features, speech, chewing, and other functions, often leading to psychological stress. Consequently, HNSCC has become the second leading cancer associated with higher suicide rates [4] after pancreatic cancer. Therefore, there is an urgent need to deeply understand the development of HNSCC and identify more treatment targets aiming at prolonging and improving patients’ lives.

CSNK1D, also known as CK1δ, belongs to the CK1 (formerly named casein kinase 1) family. As a serine/threonine-specific kinase, CSNK1D comprises two lobes: the N-terminal lobe (N-lobe) mainly consisting of β-sheet strands, and the larger C-terminal lobe (C-lobe), primarily composed of α-helices and loop structures [5]. CSNK1D is considered a key player in various biological processes such as the circadian rhythm, DNA damage and cellular stress, cell cycle, mitosis, and meiosis, and specific functions associated with cytoskeleton components. CSNK1D is also involved in multiple pathological processes, including neurologic diseases and disorders, mediating drug addiction, metabolic diseases, tumorigenesis, and tumor progression. Moreover, significantly elevated CSNK1D levels have been observed in various cancer types, including breast carcinoma [6, 7], hepatocellular carcinoma [8, 9], prostate cancer [10], glioblastoma [11], multiple myeloma [12], and bladder cancer [13]. Existing research reported CSNK1D as a therapeutic target for breast cancer [6] and bladder cancer [13]. Owing to its involvement in many biological processes as a kinase, an increasing number of targeted drugs, such as SR-3029 [6], have emerged.

However, it remains unclear how CSNK1D regulates the development of HNSCC. To comprehensively investigate the role of CSNK1D in HNSCC development, we conducted large-scale bioinformatics analyses, animal experiments, and cell culture-based analyses. This study extensively explored the malignant biological characteristics and prognostic value of CSNK1D in HNSCC, with a focus on the potential role of CSNK1D in regulating malignant cells. It is anticipated that delineating the landscape of CSNK1D in HNSCC could facilitate improvements in drug therapy and the clinical management of HNSCC.

## Methods

### Patient specimens and ethics

All human experiment procedures were approved by the Ethics Committee of Affiliated Stomatological Hospital of Nanjing Medical University, China (Approval No. PJ2021-138-001). All samples were obtained with the patients’ informed consent. A total of 109 paired tumor and paracancerous tissue samples from HNSCC patients with intact clinical information (70 males and 39 females, aged between 28 and 93 years old), who underwent tumor resection, were collected in the Department of Oral and Maxillofacial Surgery, Histologic and pathologic diagnoses of the tissue samples were independently confirmed by 2 experienced histopathologists. Unpaired tumor samples and normal samples from patients with incomplete clinical information were excluded. Moreover, patients who underwent preoperative treatment such as radiation or chemotherapy were also excluded from our study. Patient demographic and clinical characteristics are summarized in Supplementary Table S1.

### Cell culture

HNSCC cell lines (CAL27, HN4, HN6, HSC3, and FADU) and human oral keratinocytes (HOK) cell lines were provided by the Chinese Academy of Sciences. HN6 cells were cultured in DMEM/F-12 (Gibco) medium containing 10% fetal bovine serum (FBS) (Biological Industries) and 1% penicillin/streptomycin (Invitrogen). The other cell lines were cultured in high-glucose DMEM (Gibco) containing 10% FBS and 1% penicillin/streptomycin.

### Plasmid, siRNA, and lentivirus transfection assay

The CAL27 and HN4 cells (2 × 10^5^ per well) were seeded in 6-well plates and cultured for 24 h, and then transfected with pEGFP-C1-CSNK1D-Flag, pEGFP-C1-CSNK1D(AA9-271)-Flag, pEGFP-C1-CSNK1D(AA299-314)-Flag, pEGFP-C1-CSNK1D(AA314-415)-Flag, and pEGFP-C1-CSNK1D(AA299-415)-Flag plasmids (public protein and plasmid library) or CSNK1D siRNA (GenePharma, China) and GLI1 siRNA (Generay, China) using Lipofectamine 2000 (Invitrogen, USA). The transfection medium was refreshed after 6 h. For lentivirus transfection, the CAL27 and HN4 cells were transfected with OE-CSNK1D (Ribio) or SH-CSNK1D (GeneChem) lentivirus or control vector lentivirus. After 72 h, 2 µg/ml of puromycin (Invitrogen) was added to the medium, and the medium was cultured for another 14 days until the stably transfected cell lines were established. The transfected cells were then used for RNA extraction, immunofluorescence, co-immunoprecipitation, and Western blot assays.

### Cell counting kit‑8, IC50 and colony formation assays

For the cell counting kit‑8 assay, 2000 cells were seeded in a 96-well plate, the cell absorbance was measured at 450 nm at 0 h after the cell adhered to the wall, and then after every 24 h for 96 h. The half-maximal inhibitory concentration (IC50) of SB-203580 against the CAL27 and HN4 cells was determined by measuring cell viability after incubation for 72 h with serial dilutions of SB-203580. For the colony formation assays, 2000 cells were seeded in a 6-well plate and cultured for 7–14 days until obvious colony formation. Thereafter, the samples were fixed in 4% paraformaldehyde, stained with 0.05% crystal violet, and imaged.

### Wound healing and transwell assay

The wound-healing assay was conducted for migration evaluation. Briefly, cells were cultivated in 6-well plates until a density of 90–95%, and a wound was created by scraping two mutually perpendicular lines along the cell monolayer in the 6-well plates. The wound healing process was observed at 0 h, 32 h and 48 h using an inverted microscope. Transwell chambers (8-μm; Corning, Acton, MA, USA) were used for the transwell assay. To assess migration, we plated 200 μl serum-free medium containing 1 × 10^5^ CAL27 and 0.75 × 10^5^ HN4 cells in the upper chambers, while 600 μl of the complete medium was added in the lower chamber. The incubation duration was 32 h for CAL27 and 36 h for HN4. Thereafter, the chambers were fixed with 4% paraformaldehyde for 30 min, stained with crystal violet (C0121, Beyotime), and photographed at 50× magnification for counting. A similar protocol was followed for invasion assessment, except cells were further required to invade the Matrigel matrix (Thermo). For each chamber, randomly select 5 fields of view for statistical analysis.

### 3D tumor sphere formation

For proliferation evaluation, 20,000 cells were cultivated per well in a 96-well ultra-low adhesion plate with a U-shaped bottom and photographed at 2 days and 7 days after cultivation. For invasion assessment, the tumor spheres were transferred into 200 µl of a mixture of cold 7.5 mg/ml matrigel basement membrane matrix (Corning) and 2 mg/ml of rat tail collagen I (Life Technologies, Carlsbad, CA, USA). The images of the spheroids were taken with a Leica inverted microscope with 50× magnification every 24 h (for 7–14 days) to monitor the extent of spheroid invasion.

### Tunel assay

To determine whether CSNK1D expression or the treatment with SB-203580 affects tumor cell apoptosis, we conducted TUNEL assays of CAL27 and HN4 cells transfected with siRNA or oe-lentivirus or treated with SB-203580. After the treatment, apoptotic cells were identified using the TUNEL BrightRed Apoptosis Detection Kit (Vazyme, China). The cells were fixed with 4% paraformaldehyde for 30 min at room temperature, washed three times with phosphate-buffered saline (PBS), and then incubated with 0.1% Triton X-100 for 5 min. After three additional washes with PBS, the cells were incubated with the TUNEL-labeling mixture for 1 h at 37 °C in the dark. The samples were then washed three times with PBS and imaged using fluorescence microscopy.

### Flow cytometry-based apoptosis analysis

Flow cytometry was employed to measure apoptosis using the TACS Annexin V-FITC Apoptosis Detection Kit (Trevigen, Inc.), according to the manufacturer’s instructions. Detached cells in the spent culture medium and trypsinized cells were collected, mixed, and centrifuged at 1000 rpm for 5 min at room temperature. The cells were washed twice with PBS and centrifuged at 1000 rpm for 5 min, before incubating the cell pellet in the Annexin V Incubation Reagent (1% Annexin V-FITC and 1× propidium iodide solution in 1× calcium-containing binding buffer) for 15 min in the dark at room temperature. The cell mixture was then diluted to 1:5 in 1× binding buffer.

### Quantitative real-time PCR (qRT-PCR)

Total RNA was extracted using Total RNA Extraction Reagent (Vazyme), and complementary DNA (cDNA) was synthesized with HiScript II Q RT SuperMix for qPCR (Vazyme), according to the manufacturers’ instructions. The RT-PCR was performed on the LightCycler® 96 Instrument (Roche, Life Science), LightCycler® 480 Instrument (Roche, Life Science) and QuantStudio™ 7 Flex real-time fluorescence quantitative PCR systems. The forward and reverse primers used were synthesized by Genscript (Nanjing, China) and are listed in supplementary materials. The comparative threshold cycle (2^−ΔΔCt^) method was employed to calculate the relative mRNA expression of FCs. Target gene expression was normalized to the reference gene *GAPDH*. All PCRs were performed in triplicate.

### Chromatin immunoprecipitation-qPCR (ChIP-qPCR)

For chromatin immunoprecipitation (ChIP), a total of 2 × 10^7^ cells were trypsinised, washed twice with PBS and collected after centrifugation. The cells were subsequently fixed with formaldehyde to crosslink and preserve protein-DNA interactions. Protein-DNA complexes were sheared via Micrococcal Nuclease digestion, followed by immunoprecipitation with ChIP-grade antibodies against GLI1 (Genetex GTX124274). The negative (Normal Rabbit IgG) control was used for protein-bound DNA sequences. The mixture was further inverse-crosslinked at 65 °C overnight, and the enriched DNA was extracted and subjected to qRT-PCR using BCL2 primer. The procedure was conducted as detailed by the manufacturer’s instructions (ChIP kit; BersinBio).

### Western blotting

Tissues and cells were harvested and lysed with the Radio immunoprecipitation assay buffer (Beyotime) containing phenylmethanesulfonyl fluoride (PMSF; Beyotime) and protease inhibitor (PI; Dalian Meilun Biotechnology), followed by centrifugation at 12,000 rpm for 10 min at 4 °C. Thereafter, sodium dodecyl sulfate-polyacrylamide gel electrophoresis (SDS-PAGE) loading buffer (Beyotime) (Quarter volume of supernatant) was added to the supernatant and boiled in a water bath for approximately 10 min. Subsequently, the protein samples were separated on 8%, 10%, 12%, and 15% sodium dodecyl sulfate-polyacrylamide gels and transferred to polyvinylidene fluoride membranes. The membranes were blocked for more than 2 h in TBST (Tris-Buffered Saline, 0.1% Tween 20 Detergent) buffer containing 5% skim milk, then incubated overnight at 4 °C with the primary antibodies. After that, the PVDF membranes were incubated with the secondary antibodies for 1 h and bound with Tanon High-sig ECL Western Blotting Substrate (180-5001; Tanon) for detection.

### Membrane, cytoplasmic and nuclear protein extraction

A total of 107 cells were harvested and washed with 1X PBS. Membrane protein extraction was performed using the Bebel® BBproExtra® Membrane protein extraction kit (Bebel, China), following the manufacturer’s protocol. The cytoplasmic and nuclear fragment protein extraction was conducted using a Nuclear protein and cytoplasmic protein extraction kit (Beyotime, China), according to the manufacturer’s protocol.

### Immunoprecipitation

Cells were harvested and lysed using Western and IP cell lysate (P0013, Beyotime) containing protease inhibitors. The collected supernatant was precleared with protein A/G agarose (P2055-2ml, Beyotime), then immunoprecipitated with primary antibodies for 1–2 h, followed by incubation with protein A/G agarose overnight. Thereafter, the precipitates were eluted and loaded onto SDS gels for immunoblotting (IB) assays.

### Tandem mass tag (TMT)-based quantitative proteomics analysis

Fresh HNSCC and paired adjacent normal tissue samples were homogenized and the samples were lysed with SDT (4%SDS, 100 mM Tris-HCl, 1 mM DTT, pH 7.6) buffer. The protein concentrations were quantified with the BCA Protein Assay Kit (Bio-Rad, USA). After tryptic digestion, the obtained peptides were desalinated and labeled with TMTsixplex™ (Thermo). The peptide mixtures were fractionated by high pH reverse-phase HPLC using an Agilent 300 Extend C18 column, after which the peptides were combined into 18 fractions and dried by vacuum centrifugation. The tryptic peptides were dissolved in 0.1% formic acid and directly loaded onto an analytical column (Thermo Scientific EASY column, 10 cm, ID 75 µm, 3 µm, C18-A2). The resulting peptides were subjected to a nanospray ionization (NSI), followed by tandem mass spectrometry (MS/MS) in a Q Exactive™ Plus (Thermo) coupled to ultra-performance liquid chromatography (UPLC). The *m*/*z* scan range was 300–1800 for the full scan, and the fixed first mass was set as 200 *m*/*z*.

### Immunohistochemistry and immunofluorescence

Tissues were fixed with formalin, embedded in paraffin, and sectioned into 4-μm thick. The sections were deparaffinized in xylene, rehydrated in a graded series of ethanol, boiled with sodium citrate buffer (10 mM sodium citrate, 0.05% Tween 20, pH 6.0) to retrieve antigens for 15 min, and cooled to room temperature. The sections were then blocked with 3% H_2_O_2_ for 20 min, followed by goat serum for 1 h at 37 °C, and incubated with the primary and secondary antibodies, developed with diaminobenzidine (DAB), counterstained with hematoxylin, and imaged with an upright microscope (Leica, Germany). For immunofluorescence, cells were fixed in 4% paraformaldehyde for 30 min at room temperature, permeabilized with 0.25% Triton X-100, and then blocked with goat serum for 1 h at room temperature. Cells were washed and incubated with Fluor-labeled secondary antibody after 12–16 h of incubation with the primary antibody dilution, followed by imaging using fluorescence microscopy.

### In vivo orthotopic xenograft model

All animal experiment procedures were approved by the Ethics Committee of Nanjing Medical University, China (Approval No. IACUC 2305020). In total, 20 female BALB/c nude mice (5–6 weeks old) were obtained from Beijing Vital River Laboratory Animal Technology Co., Ltd. (Beijing, China) and randomized into 4 groups. The flanks of nude mice were then injected with control- or sh-CSNK1D-transfected HN4 cells, or with control- or LV-CSNK1D-transfected CAL27 cells. Each mouse received 5 × 10^6^ cells in 200 µl of DMEM and Matrigel.

### In vivo translational experiment

Ten female BALB/c nude mice (5–6 weeks old) were obtained from Beijing Vital River Laboratory Animal Technology Co., Ltd. (Beijing, China). After adapting to the environment, the flanks of each mouse were injected with 5 × 10^6^ HN4 cells. On day 14 after implantation, the mice were randomized into 2 groups and treated every four days (for 16 days) by intraperitoneal injection of 1 μM/kg body weight/day of SB203580 in 1 ml of PBS or plain PBS as a control. Mice weight was measured before every subcutaneous injection. On day 30, mice were euthanized, and then subcutaneous tumors were collected for analysis.

### Molecular docking

For protein docking, the structural files of CSNK1D (PDB ID: 6PXN), PTCH1 (PDB ID: 6RMG), and SHH (PDB ID: 6PJV) were downloaded from the PDB database. The protein structures were imported into Pymol2.3.0 to remove water molecules and heteroatoms from the crystal structure, and uploaded to the HDOCK SERVER software (tp://huanglab.phys.hust.edu.cn/) for docking. Finally, the optimal docking combination (Model No. 1) was selected and subjected to graphical analysis in Pymol2.3.0. For protein and small molecule compound docking, the SDF file formats of the main active ingredients of the core drug were obtained from the PubChem database, and the key target protein structures from the PDB database. Pymol software was used to optimize the target by clearing water molecules and small molecule ligands, while AutoDock Tools was used for hydrogenation and charge treatment, and the resulting structure was saved in the pdbqt format. The molecular docking of key targets as receptors and their corresponding active ingredients as ligands was performed with Vina within Pyrx software to calculate binding energy and output result files. Finally, PyMol software was used for result visualization. The affinity (kcal/mol) value represents the binding ability of two molecules, and the lower the binding ability, the more stable the ligand-receptor binding. The lower the binding energy, the better the binding.

### Data acquisition and Bioinformatics analysis

Differential gene expression analysis and Kyoto Encyclopedia of Genes and Genomes analysis were performed using proteomics data to determine the differentially expressed genes and significantly enriched pathways. The gene expression omnibus (GEO) datasets of GSE74530 and GSE78060 (http://www.ncbi.nlm.nih.gov/gds/) were analyzed using R software (http://www.bioconductor.org/) to evaluate the CSNK1D expression profile in human HNSCC cancer and normal pancreatic tissues. The Kaplan–Meier plotter was used to analyze the relationship between the CSNK1D mRNA expression level and overall survival (OS) of HNSCC patients using GEPIA (http://gepia.cancer-pku.cn/). The HNSCC gene expression dataset was downloaded from the TCGA database (https://www.cancer.gov/ccg/research/genome-sequencing/tcga) and divided into two groups (CSNK1D-high and CSNK1D-low groups), based on the CSNK1D expression. For the significantly enriched pathways, Gene Set Enrichment Analysis was performed after differential gene expression analysis. TRRUST (https://www.grnpedia.org/trrust/) and chip-atlas (https://chip-atlas.org/) were employed to obtain the possible target gene regulated transcriptionally by GLI1. Jaspar (https://jaspar.elixir.no/) was used to determine the binding of GLI1 to the promoter of BCL2. Moreover, DGIDB (https://dgidb.org/) and LI000FWD (https://maayanlab.cloud/l1000fwd/result/650086ebc99977002d2bc8d2) were used to obtain the small molecule compounds targeting CSNK1D, while Venny 2.0 (https://bioinfogp.cnb.csic.es/tools/venny/) was employed to generate Venn diagram.

### Statistical analysis

Each experiment was performed at least in three independent replicates, and the results were expressed as the mean ± standard deviation (SD) using GraphPad Prism version 9.0. Two-tailed unpaired Student t-test, one-way and two-way ANOVA tests (Bonferroni’s post-hoc test), and Mann–Whitney tests were conducted where appropriate.

## Results

### CSNK1D is highly expressed in HNSCC tissues and the expression is positively correlated with disease progression and prognosis

**Fig. 1:**
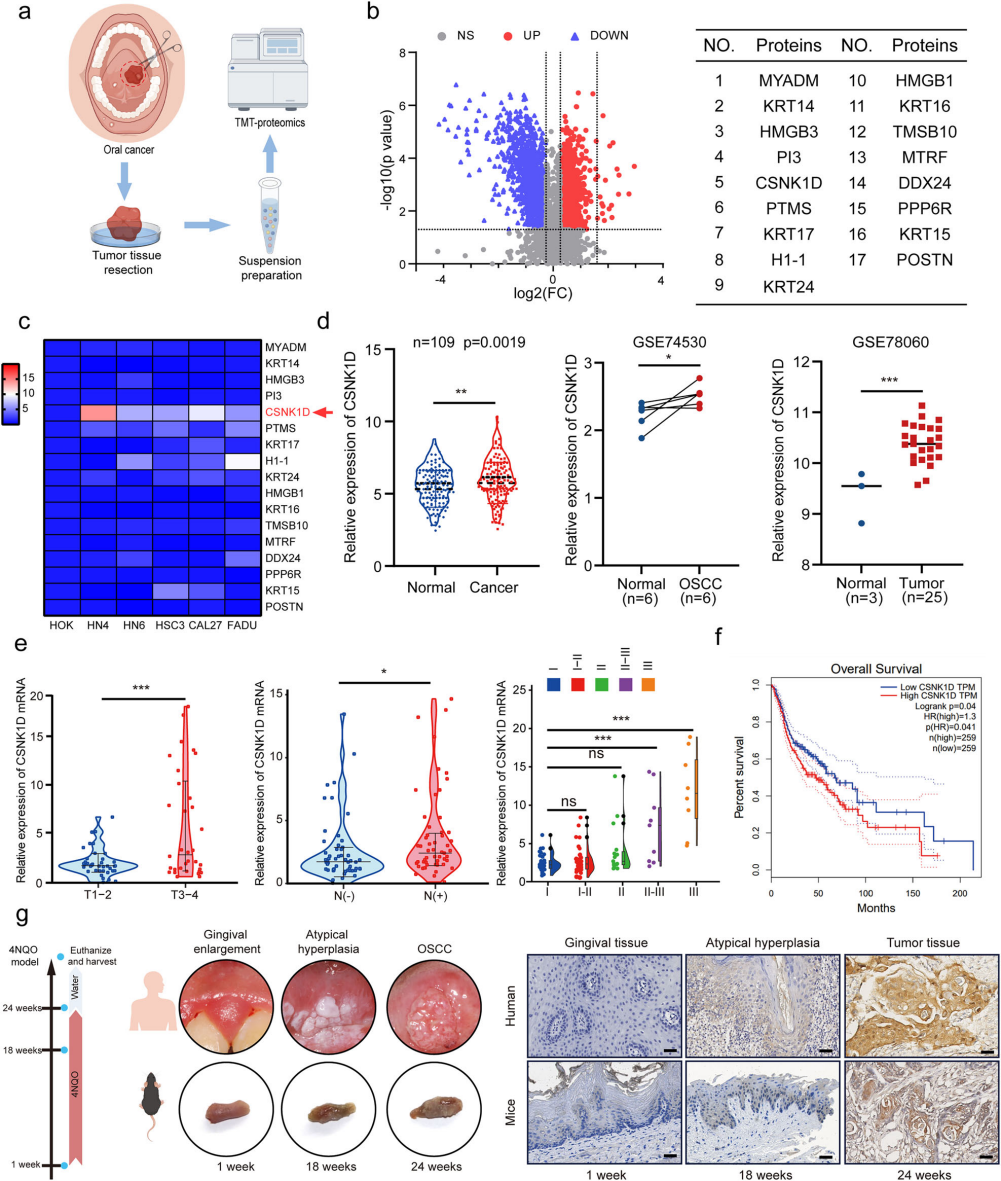
The expression, clinical and prognostic characteristics of CSNK1D in head and neck cancer. **a** The schematic representation of TMT-proteomics analysis of three paired oral squamous cell carcinoma (HNSCC) tissues and normal tissues. **b** Volcano plot of the differentially expressed genes (DECs) in three paired HNSCC tissues (|FC| > 1.2 and *P* < 0.05). **c** Comparison of the 17 upregulated genes that were detected in five groups of HNSCC cell lines and HOK by qRT-PCR. CSNK1D had the highest relative expression. **d** qRT-PCR analysis of CSNK1D expression in 111 paired HNSCC tissues and normal tissues. Comparison of CSNK1D expression in human HNSCC tissue samples and matched non-neoplastic tissue in Gene Expression Omnibus (GEO) datasets GSE74530 and GSE78060. **e** The CSNK1D expression disparity in HNSCC with different clinical traits in 111 paired HNSCC tissues and normal tissues. **f** Kaplan–Meier plots of the survival time in The Cancer Genome Atlas (TCGA) HNSCC cohort of patients (*n* = 520) with high vs low expression of CSNK1D in the primary tumor (segregated using median expression level as cut-off value). **g** Schematic diagram indicating the construction of a mouse HNSCC model fed with 4NQO and images of immunohistochemical samples. Representative IHC staining images of CSNK1D in human and mouse normal mucosa, atypical hyperplasia and HNSCC tissues (scale bar, 50 μm). IHC immunohistochemistry, HNSCC Head and neck squamous cell carcinoma.

To screen dysregulated proteins in human HNSCC, we performed TMT-based quantitative proteomics analysis in 30 human HNSCC tumor samples and paired adjacent normal tissues (Fig. 1a). A total of 4987 proteins were identified. We screened for the differentially expressed proteins using the criteria of fold change ≥1.2 or ≤0.83 and *p* < 0.05 and identified 2200 differentially expressed proteins, including 1006 up-regulated and 1194 down-regulated proteins (Fig. 1b). The top 17 upregulated proteins were detected included MYADM among others (Fig. 1b). qRT-PCR was performed to determine the expression levels of these proteins in HOK cells and HNSCC cell lines, and the results revealed CSNK1D overexpression in all 5 HNSCC cell lines (Fig. 1c). To further verify the expression characteristics of CSNK1D in HNSCC, we first verified the mRNA level of CSNK1D in HNSCC tissues relative to paracancerous tissues. The results indicated that the CSNK1D expression level was significantly higher in HNSCC tissues than in normal tissues (Fig. 1d). Consistently, CSNK1D levels were increased in HNSCC tissues relative to normal tissues, according to GSE74530 and GSE78060 patterns in GEO dataset. Furthermore, we observed higher mRNA levels of CSNK1D in HNSCC patients with a higher pathological grade and at advanced clinical stage than those with the low grade and at an early stage, which was also observed in HNSCC patients with lymph node metastasis (Fig. 1e). Moreover, the expression level of CSNK1D negatively correlated with the survival rate (*p* = 0.04) in HNSCC patients (Fig. 1f). To gain insight into the role of CSNK1D in HNSCC progression, we measured CSNK1D expression in normal, atypical hyperplasia or cancer tissues of human and 4NQO-induced HNSCC mice via IHC analysis. CSNK1D expression was gradually elevated in the three developmental stages (Fig. 1g). In conclusion, CSNK1D was highly expressed in HNSCC tissues and its expression was positively correlated with disease progression and prognosis.

### CSNK1D promotes the proliferation and inhibits apoptosis of HNSCC cells

**Fig. 2:**
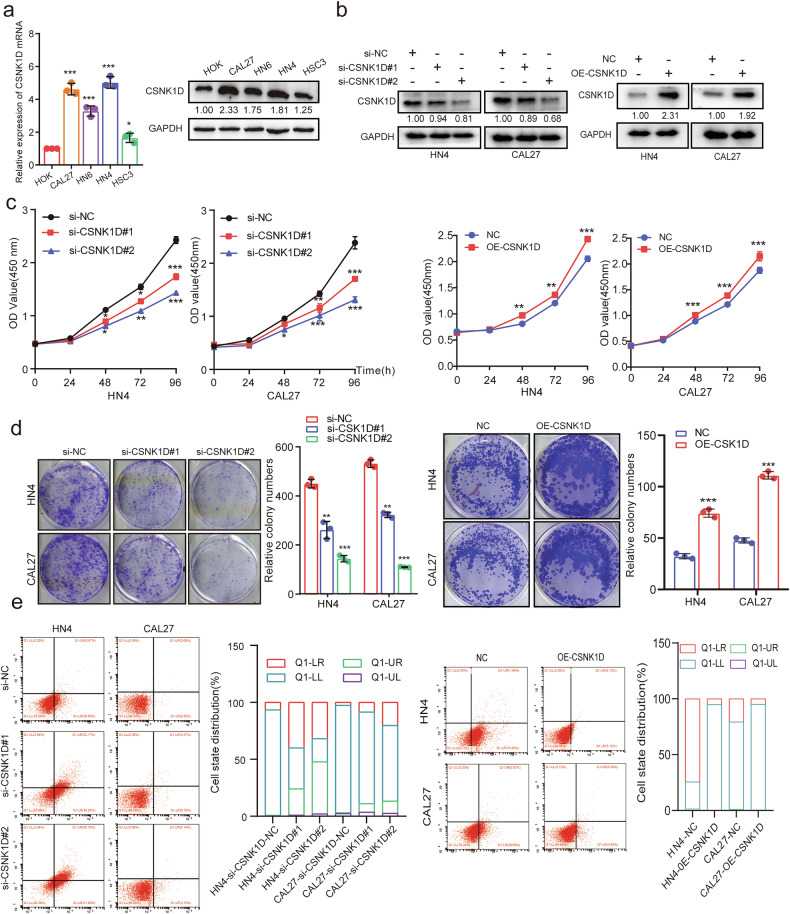
Effect of CSNK1D on the proliferation and apoptosis potential of HNSCC cells in vitro. **a** The mRNA and protein expression of CSNK1D were in the five groups of HNSCC cell lines and HOK, as shown by qRT-PCR and Western blot analysis. HNSCC, Head and neck squamous cell carcinoma. **b** Western blot analysis of CSNK1D protein levels assessed in HN4 and CAL27 cells treated with si-CSNK1D or oe-CSNK1D. **c** Growth curves of CSNK1D-silencing or CSNK1D overexpression and control HN4 and CAL27 cells as measured by a CCK-8 assay, **P < 0.01, ***P < 0.001. **d** Representative images and quantitative analysis of anchor-dependent colony formation assays in HN4 and CAL27 cells, **P < 0.01, ***P < 0.001, error bar values represent the standard deviation (SD). **e** Apoptosis flow cytometry of si-CSNK1D or oe-CSNK1D and corresponding control HN4 and CAL27 cells (scale bar, 100 μm).

**Fig. 3:**
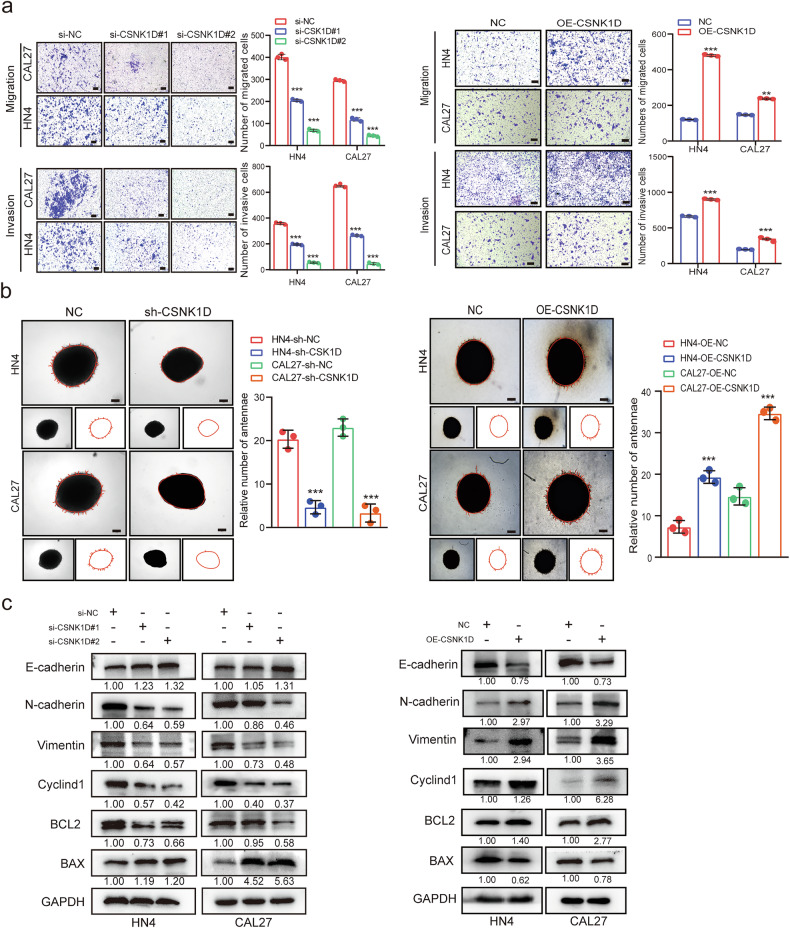
Effects of the elevated CSNK1D on the tumor malignant potential of HNSCC cells in vitro. **a** Migration and invasion of HN4 and CAL27 cells in the control and CSNK1D-knockdown or CSNK1D-overexpressing groups (*n* = 3) (scale bar, 200 μm). **b** Spheroid invasion assays showing enhanced cell invasion of the CSNK1D overexpressing or CSNK1D-silencing and control CAL27 and HN4 cells (scale bar, 200 μm). **c** Western blot analysis of E-cadherin, N-cadherin, vimentin, Cyclind1, Bcl2, and Bax proteins in HN4 and CAL27 cells after CSNK1D overexpression.

To further explore the specific role of CSNK1D in HNSCC, we first evaluated CSNK1D expression in the normal HOK cells and HNSCC cell lines (CAL27, HN4, HN6, and HSC3) via RT-PCR and Western blot analysis. We observed higher expression levels of CSNK1D in HNSCC cell lines than in HOK cells, especially in the CAL27 and HN4 cell lines (Fig. 2a). Thus, CAL27 and HN4 HNSCC cells were selected for subsequent experiments. The expression and distribution of CSNK1D in HNSCC cells were first investigated via immunofluorescence and western blot. We observed that CSNK1D was predominantly localized in the cytoplasm of tumor cells, with limited nuclear distribution (Fig. S1a, b). To investigate the effects of CSNK1D on the HNSCC cells, we constructed the si-RNA to knockdown (si-CSNK1D) or overexpression lentivirus to overexpress the CSNK1D (OE-CSNK1D) in the cells, which inhibited or enhanced the levels of CSNK1D protein (Fig. 2b) and mRNA (Fig. S1c, d). We also performed the CCK8 assay (Fig. 2c), cloning experiments (Fig. 2d) and 3D tumor sphere formation assay (Fig. S1e) to elute the effects of CSNK1D on the proliferation. Our data illustrated that CSNK1D knockdown inhibits the proliferation of CAL27 and HN4 cells, while its overexpression enhanced the proliferation of the cells. Moreover, flow cytometry data showed that CSNK1D knockdown enhanced the apoptosis of CAL27 and HN4 cells, which was decreased after overexpression of CSNK1D (Fig. 2e). This was consistent with the Tunel assay results (Fig. S1f). In summary, our data suggested that CSNK1D promoted the proliferation and inhibited the apoptosis of HNSCC cells.

### CSNK1D enhances the migration and the invasion of HNSCC cells

The effects of CSNK1D on the migration and invasion of HNSCC cells were investigated via wound healing, transwell and 3D tumor sphere formation assays. As shown in Fig. 3a and Fig. S2a, the migration ability was inhibited in the si-CSNK1D group but increased in the OE-CSNK1D group compared with the control group. Furthermore, the transwell and 3D tumor sphere formation assay results indicated reduced invasion ability in the si-CSNK1D group compared with the control group, while CSNK1D overexpression facilitated the invasion of HNSCC cells in the OE-CSNK1D group (Fig. 3a, b). Finally, the proliferation-, apoptosis- and EMT-associated indexes were analyzed via Western blotting (Fig. 3c and Fig. S2b). Our data showed that the results were consistent with that of the in vitro cell phenotype experiment. Collectively, CSNK1D promoted the proliferation, inhibited apoptosis, and facilitated the migration and invasion of HNSCC cells.

### CSNK1D promotes HNSCC progression by activating the Sonic Hedgehog pathway

**Fig. 4:**
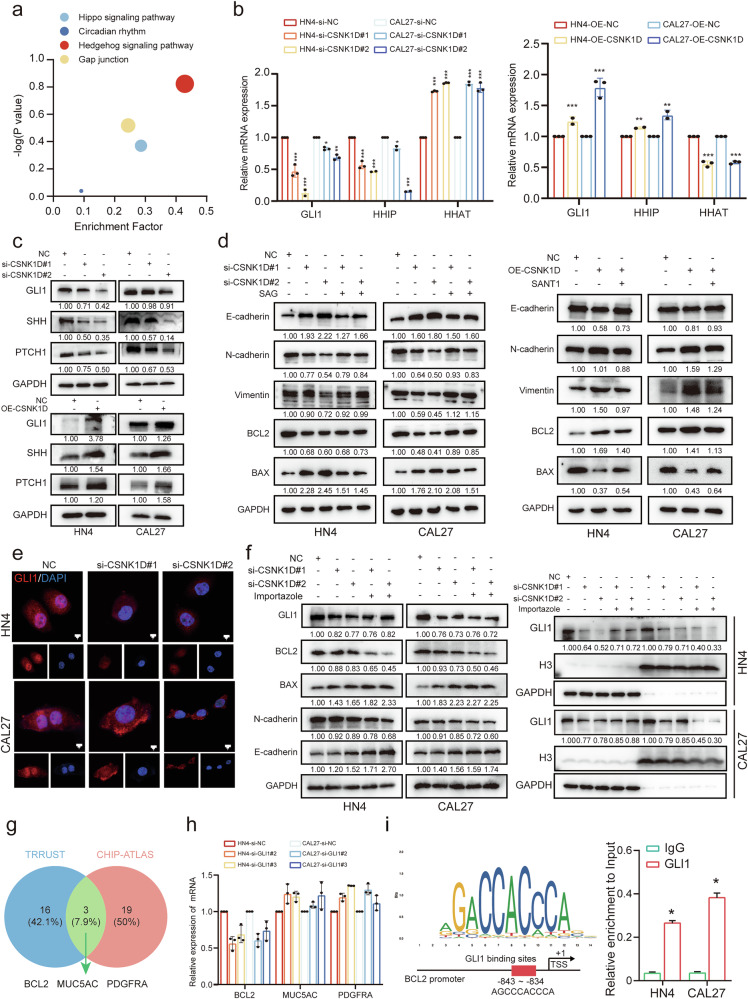
The positive correlation of CSNK1D with the hedgehog pathway in HNSCC. **a** The dot blot of gene enrichment analysis. **b** Expression levels of key genes in the hedgehog pathway in HN4 and CAL27 cells in the control (con) and CSNK1D-silencing (si-1, si-2) or CSNK1D-overexpressing (ov) groups. **c** Western blot analysis of GLI1, SHH and PTCH1 proteins in HN4 and CAL27 cells after CSNK1D silencing or overexpression (*n* = 3). **d** Western bot analysis of E-cadherin, N-cadherin, vimentin, Bcl2, and Bax proteins in HN4 and CAL27 cells after CSNK1D silencing and addition of the hedgehog pathway agonist SAG or overexpression and addition of the hedgehog pathway inhibitor SANT1 (*n* = 3). **e** Immunofluorescence analysis showing the distribution of GLI1 (red) in the CSNK1D knockdown and control HN4 and CAL27 cells. (scale bar, 25 μm). **f** Protein expression of Gli1, Bcl2, Bax, N-cadherin, and E-cadherin and distribution of GLI1 in nucleus and cytoplasm of HN4 and CAL27 cells treated with importazole (nuclear transport inhibiter) after CSNK1D silencing. **g** Venn diagram demonstrating the overlapping of the target genes of the transcription factor GLI1 predicted by TRRUST and CHIP-ATLAS. **h** Detection of three candidate mRNA levels by qRT-PCR upon GLI1 silencing in HN4 and CAL27 cells (*n* = 3). **i** The binding site within the GLI1 and BCL2 promoter region, as determined via JASPAR. Chromatin immunoprecipitation demonstrated that the GLI1 antibody could effectively precipitate with the −843 to −834 region of the BCL2 promoter.

To decipher the downstream signaling pathways of CSNK1D, we analyzed the data from the KEGG (Fig. 4a) and GSEA (Fig. S3a) databases and observed significant changes in the HH pathway. Therefore, we evaluated some indicators of Hedgehog pathway. The levels of mRNAs of indicators, such as GLI1 and HHIP, were decreased after CSNK1D knockdown, but were enhanced by the overexpression of CSNK1D (Fig. 4b). Moreover, the expression of proteins in the HH pathway, such as GLI1, SHH, and PTCH1, was reduced by the si-CSNK1D treatment, but elevated by OE-CSNK1D (Fig. 4c). To further verify whether CSNK1D affected tumor development through the HH pathway, we applied the agonists of the HH pathway (SAGs) into the CSNK1D-knockdown CAL27 or HN4 cells, and the HH pathway inhibitor (SANT1) into CSNK1D-overexpressed cells. Our results indicated that SAGs alleviated the inhibitory effects of CSNK1D-knockdown on the proliferation and EMT, while SANT1 inhibited the effects of OE-CSNK1D on proliferation (Fig. 4d). All these suggested that the HH pathway was attributed to the effects of CSNK1D on HNSCC prognosis.

GLI1 mediates the biological functions of the Hedgehog pathway by regulating the transcription of target genes in the nucleus. The present study found that the si-CSNK1D or OE-CSNK1D treatment affected the nucleus entry of GLI1. CSNK1D knockdown reduced GLI1 levels in the nucleus (Fig. 4e), while OE-CSNK1D increased it (Fig. S3b). This was consistent with the western blot results of cytoplasmic and nuclear protein isolation (Fig. S3c), suggesting that CSNK1D regulated the nucleus entry of GLI1. Furthermore, we treated the cells with importazole, a nucleus transport inhibitor, and observed that the decreased protein levels caused by CSNK1D knockdown were more alleviated (Fig. 4f), while importazole inhibited the protein expression increase caused by OE-CSNK1D (Fig. S3d). Moreover, there were lower nucleus GLI1 levels in both si-CSNK1D and OE-CSNK1D cells under the importazole treatment than in the control (Fig. 4f and Fig. S3e). To identify the downstream target genes for GLI1, we conducted bioinformatics analysis using two databases, and found that BCL2, MUC5AC, and PDGFRA genes might be the targets (Fig. 4g). Thus, we evaluated the transcription levels of these three genes in GLI1-deficient cells. We observed that BCL2 was significantly reduced in GLI1-deficient cells compared to other genes (Fig. 4h). The bioinformatics analysis using the Jaspar database suggested the possible binding between GLI1 and BCL2 (Fig. 4i), and the CHIP test results on HN4 and CAL27 cells showed the enrichment of GLI1 in BCL2, suggesting that GLI1 may be attributed to the transcription of BCL2 (Fig. 4i). In summary, our data indicated that CSNK1D promoted HNSCC cancer progression by increasing the nucleus entry of GLI1 and activating the hedgehog GLI1/BCL2 signaling pathway.

### CSNK1D (AA314-415) promotes the formation of CSNK1D/SHH/PTCH1 complex and regulates the nucleus entry of GLI1

**Fig. 5:**
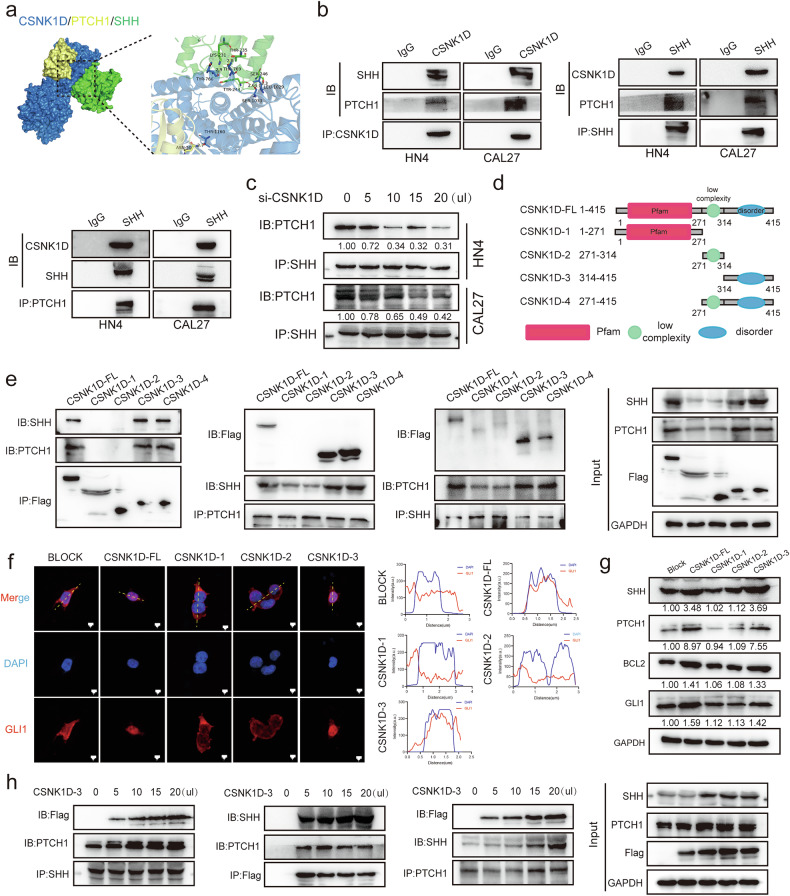
Effects of CSNK1D on the binding of SHH to PTCH1 in the hedgehog pathway. **a** Molecular docking of CSNK1D with SHH and PTCH1. **b** The CSNK1D- SHH and CSNK1D-PTCH1 complexes. **c** Co-immunoprecipitation between SHH and PTCH1 in HN4 and CAL27 cells treated with different concentrations of si-CSNK1D. **d** Truncated versions of FLAG-CSNK1D produced from the CSNK1D domain. **e** Co-immunoprecipitation after transfection of different truncated versions of FLAG-CSNK1D. **f** The distribution of GLI1 in 293T cells treated with Block, CSNK1D-FL, CSNK1D-1, CSNK1D-2, and CSNK1D-3. **g** Protein expression of SHH, PTCH1, BCL2, and GLI1 in 293T cells treated with Block, CSNK1D-FL, CSNK1D-1, CSNK1D-2, and CSNK1D-3. **h** Co-immunoprecipitation after transfection of different concentrations of CSNK1D-3.

The binding of the Hh ligand (SHH) to PTCH1 initiates the HH signaling pathway and regulates the activation of GLI in cells. We performed molecular docking analysis, and found that CSNK1D may act as the bridging protein in the binding of SHH to PTCH1 (Fig. 5a). Thus, we further explored the effect of CSNK1D on the binding of SHH to PTCH1 via co-immunoprecipitation, and observed that three molecules could be co-immunoprecipitated with each other in the HN4 and CAL27 cells (Fig. 5b). Moreover after the CSNK1D knockdown decreased the binding of SHH to PTCH1 in a dose-dependent manner (Fig. 5c). To explore how CSNK1D was involved in the binding of SHH to PTCH1, we constructed truncated plasmids (CSNK1D-FL: full length, CSNK1D-1: CSNK1D with the C-terminus deletion of 272–415 amino acids, CSNK1D-2: CSNK1D with low complexity domain, CSNK1D-3: CSNK1D with disorder domain, CSNK1D-4: CSNK1D with the truncated 271–415 amino acids) (Fig. 5d). These truncated plasmids were transfected into 293T cells, and the co-IP results indicated that CSNK1D-FL, CSNK1D-3 and CSNK1D-4 could bind SHH and PTCH1. This suggested that the C-terminus 314–415 domain of CSNK1D attributed to the binding of SHH to PTCH1 (Fig. 5e). Consistently, the transfection of CSNK1D-FL and CSNK1D-3 increased the nucleation proportion of GLI1, while nucleation levels of GLI1 remained unchanged in BLOCK, CSNK1D-1 and CSNK1D-2 transfection cells (Fig. 5f). Furthermore, the enhanced expression of SHH, PTCH1, BCL2, and GLI1 indicated that CSNK1D-FL and CSNK1D-3 can function (Fig. 5g). Immunoprecipitation was performed after transfecting different concentrations of CSNK1D-3 fragments and the result showed that the binding of SHH and PTCH1 increased as the concentration of CSNK1D-3 increased, suggesting that CSNK1D-3 could indeed promote the binding of SHH to PTCH1 (Fig. 5h). In summary, CSNK1D-3 (AA314-415) promoted the formation of CSNK1D/SHH/PTCH1 complex and regulated the entry of GLI1 into the nucleus.

### CSNK1D promotes tumorigenesis in mice

**Fig. 6:**
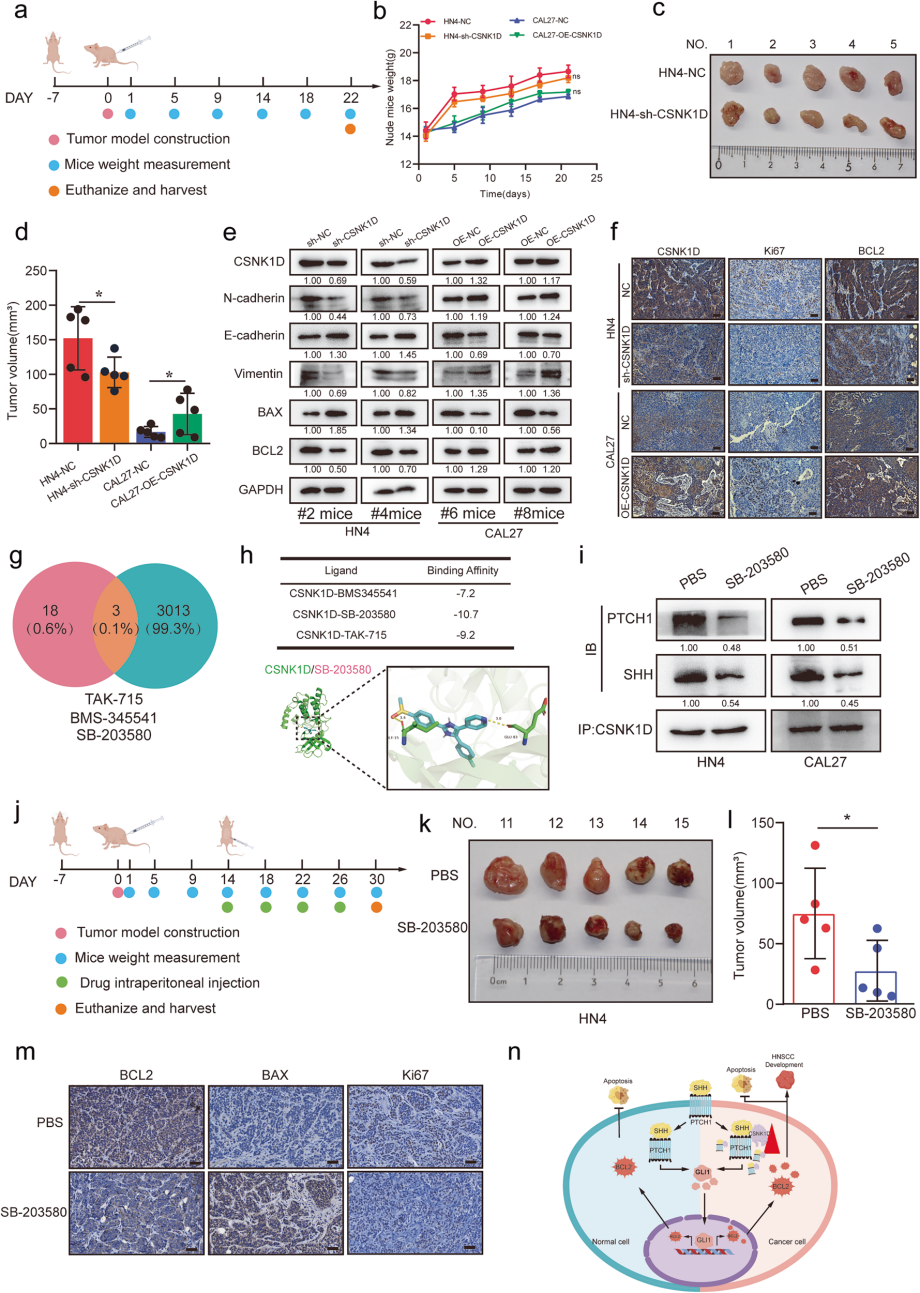
Effect of CSNK1D knockdown on the orthotopic head and neck tumor growth in vivo. **a** Schematic diagram of the subcutaneous implantation of HNSCC cell lines in nude mice. **b** Evaluation of mouse body weights every four days for two weeks after tumor cell injection. **c** Tumor images of the HN4-NC and HN4-sh-CSNK1D cells in subcutaneous tissues. **d** Sizes of tumors in the HN4-NC and HN4-sh-CSNK1D cells in subcutaneous tissues at the end of the experiment. **e** Protein expression of CSNK1D, N-cadherin, E-cadherin, vimentin, Bax, and Bcl2 in mouse orthotopic tumor tissues. **f** Representative IHC staining images of CSNK1D, ki67 and BCL2 in mouse orthotopic tumor tissues (scale bar, 100 μm). **g** Venn diagram demonstrating the overlapping of the drugs targeting CSNK1D predicted by DGIDB and L1000FWB. **h** List of binding affinity of CSNK1D and predicted drugs, Pattern diagram of the combination of CSNK1D and drug SB-203580. **i** Co-ip between CSNK1D, shh and ptch1 in HN4 and CAL27 cells treated with SB-203580. **j** Schematic diagram of orthotopic tumor model treated with PBS or SB-203580. **k** Representative images of tumors from orthotopic tumor model treated with phosphate-buffered saline (PBS) or SB-203580. **l** Sizes of tumors in the HN4 cells treated with PBS or SB-203580 in subcutaneous tissues at the end of the experiment. **m** Representative IHC staining images of BCL2, BAX and KI67 in orthotopic tumor tissues treated with PBS or SB-203580 (scale bar, 100 μm). **n** Schematic illustration showing the suggested mechanism by which CSNK1D functions as an oncogene for HNSCC apoptosis through the hedgehog pathway. CSNK1D contributes to stable SHH and PTCH1 complexes to promote the nucleus entry of GLI1 to regulate BCL2 transcription.

To examine the role of CSNK1D in tumorigenesis in vivo, we injected 5 × 10^6^ HN4 cells with sh-CSNK1D/sh-NC and CAL27 cells with OE-CSNK1D/OE-NC into the subcutaneous tissues of nude mice, as shown in Fig. 6a. Though the change in CSNK1D expression had no marked effects on the weight of mice (Fig. 6b), while CSNK1D deficit reduced tumor growth (Fig. 6c, d), and CSNK1D overexpression enhanced the growth (Fig. S4a, b). Immunoblotting analyses showed that the CSNK1D expression levels were correlated with the levels of E-cadherin, N-cadherin, Vimentin, BCL2, and Bax in the tumor tissues of nude mice (Fig. 6e). These findings were further validated by immunohistochemistry (IHC) analyses of the tumor specimens (Fig. 6f and Fig. S4c). For clinical translation, the public databases DGIDB and LIF000WD were searched and three small molecule compounds were obtained: TAK-715, BMS-345541 and SB-203580 (Fig. 6g). After molecular docking of these three small molecule compounds with CSNK1D, we observed that SB-203580 had the highest binding affinity with CSNK1D compared to other molecules (Fig. 6h). Therefore, SB-203580 was tested in the subsequent cell experiments. Using CCK8, we determined that the IC50 of SB-203580 in the HN4 cell line was 17.10 μm, and was 14.78 μm in CAL27 (Fig. S4d); thus 17 μm SB-203580 was used in the subsequent experiments. The SB-203580 treatment reduced the migration ability (Fig. S4e) and the invasion ability of HNSCC cells (Fig. S4f). Furthermore, the cloning and tunnel experiment results showed that SB-203580 reduced the proliferation ability of cells (Fig. S4g) and increased the apoptosis rate of cells (Fig. S4h). By further examining the protein levels of CSNK1D, GLI1, BCL2, and BAX in cells after the SB-203580 treatment, we observed that the expression levels of GLI1 and BCL2 were decreased, while that of BAX was increased and CSNK1D was not changed (Fig. S4i). Subsequent immunoprecipitation assay indicated that the SB-203580 treatment reduced the binding of CSNK1D to SHH and PTCH1 (Fig. 6i). The data indicated that SB-203580 only affected the function of CSNK1D without its expression level. To further verify the inhibitory effect of SB-20358 on CSNK1D, we constructed the subcutaneous tumor model by injecting 5 × 10^5^ of HN4 cells into the nude mice (Fig. 6j). The body weight of the nude mice was measured every four days (for 16 days). The nude mice were randomly divided into two groups when the tumor volume reached the same size to ensure that the tumor volume of the two groups was similar. Subsequently, the mice were injected intraperitoneally with PBS or SB-203580 every four days for two weeks, after which the mice were euthanized and the tumors were removed. We observed that the tumor volume (Fig. 6l) and weight (Fig. S4k) in the SB-203580 group were lower than those in the PBS group. Similarly, the weight of mice treated with SB-203580 was smaller than that of the PBS group, but there was no statistical significance between them (Fig. S4j). We used a portion of the tissues to measure the protein levels of BCL2, BAX, and KI67 (Fig. S4l), and another portion for immunohistochemistry to evaluate the expression of BCL2, BAX, and KI67 (Fig. 6m). Consistent with the above data, the SB-203580 treatment significantly reduced the BCL2, GLI1 and KI67 levels, but increased the levels of BAX. In conclusion, SB-203580 inhibited the growth of oral squamous cell carcinoma by regulating the function of CSNK1D.

## Discussion

In this study, we identified CSNK1D as a novel promoter of HNSCC. Elevated expression of CSNK1D in the HNSCC tissues correlated with poor prognosis while suppressing CSNK1D expression inhibited HNSCC growth and metastasis both in vitro and in vivo. Additionally, we found that CSNK1D protein promoted the development of HNSCC by regulating the hedgehog pathway. Additionally, CSNK1D (AA314-415) promoted the formation of CSNK1D/SHH/PTCH1 complex and regulated the entry of GLI1 into the nucleus. In general, our results indicate that CSNK1D acts as a protumor factor in HNSCC and might serve as a prognostic indicator and therapeutic target for HNSCC treatment (Fig. 6n).

Previous studies demonstrated the oncogenic role of CSNK1D in various cancers, including breast carcinoma [6, 7], hepatocellular carcinoma [8, 9], prostate cancer [10], glioblastoma [11], multiple myeloma [12], and bladder cancer [13]. However, research on CSNK1D in HNSCC remains limited. Only one study has reported on the expression of CSNK1D in the low T-stage oral squamous cell carcinoma, which involved an immunohistochemical assay [14]. Through large-scale bioinformatics analysis and in vitro and in vivo assays, this study comprehensively evaluated the expression pattern and effect of CSNK1D in HNSCC. Our analysis validated that CSNK1D functions as a positive regulator of tumor growth, providing a novel and comprehensive demonstration of the involved mechanism.

Hedgehog signaling is implicated in various cancers. Ligand-independent pathway activation results from mutations in the Hedgehog pathway components, including PTCH1, which has been correlated with basal cell carcinoma, medulloblastoma, and several other types of cancer [15]. The ligand-dependent pathway activation has also been linked to multiple cancers including gastrointestinal tumors, and prostate and pancreatic cancers [16, 17]. The two key steps in the ligand-dependent pathway are the binding of the hedgehog ligand to PTCH1 and the activator (CiA/GliA) forms of Cubitus interruptus (Ci)/glioma-associated oncogene homolog (Gli) transcription factors and translocating into the nucleus [17]. The formation of the SHH-PTCH1 complex is the initiation step crucial for the activation of the ligand-dependent Hedgehog pathway. Previous research confirmed that TSPAN8 promotes cancer cell stemness by directly interacting with PTCH1 and enhancing the stability of the SHH-PTCH1 complex [18]. Moreover, SLITRK5, a Hh co-receptor, inhibits Hedgehog signaling by competing with PTCH1 for Hh ligand binding and negatively regulates osteoblasts [19]. Previously, it has been proven that CSNK1D is associated with the components that affect the HH pathway such as the full-length Cubitus interruptus positive transcription factor (CiA) which is phosphorylated by CK1δ and protected from proteasomal degradation [20]. However, evidence regarding the role of CK1 in the formation of the SHH-PTCH1 complex is lacking. Our results revealed that CSNK1D can promote the formation of the CSNK1D-SHH-PTCH1 complex, thereby activating hedgehog signaling. It is generally believed that the kinase domain is the main functional domain of CSNK1D and its mutant form is usually used to evaluate the functionality of the protein. However, the activity of the kinase segment has recently been shown to exhibit a contradictory effect on cancer. For example, decreased kinase activity promoted the development of colorectal adenomas [21]. Conversely, impaired CK1δ activity inhibited SV40-induced cellular transformation in vitro and mouse mammary carcinogenesis in vivo [22]. These results indicate that the function of CSNK1D may be independent of the kinase activity and may differ in a context-dependent way. We obtained a clear segment (AA314-415) that binds the SHH-PTCH1 complex by constructing a truncated plasmid to demonstrate an alternative way to influence hedgehog signaling. These findings align with previous literature, indicating that the regulatory function of CSNK1D could be independent of the kinase section [23]. Thus, the segment could be a potential novel therapeutic target for HNSCC treatment.

Three closely related members of the Gli gene family, Gli1, Gli2, and Gli3, are the key components of the Hedgehog pathway in vertebrates. Gli2 and Gli3 were considered the main transcriptional activator (GliA) and repressor (GliR), of the Hh pathway in mammals, respectively [24–27]. Contrary to this report, Gli1 rather than Gli2 was the principal activator of Hh signaling in early zebrafish and Xenopus embryos [28, 29]. However, in response to Hh stimulation, Gli2 acts as the principal activator to trigger the expression of Gli1 and other Gli targets. Gli1 is a robust activator and potentiates the transcriptional output of Hh signaling. Therefore, elevated GLI1 activity could serve as a marker for the activation of the HH signaling pathway. Moreover, recent reports have demonstrated that elevated Gli activity was linked to transcriptional activation of Gli genes, posttranslational modification of Gli proteins [30] and nuclear localization. Therefore, this study focused more on the nucleus entry of Gli proteins, especially Gli1. We also demonstrated that CSNK1D can indeed promote the translocation of Gli1 into the nucleus by regulating the expression of CSNK1D and binding nuclear transport inhibitors. Several studies also indicated that elevated Gli activity has been observed in various human cancers, including basal cell carcinoma, breast cancer, gastrointestinal cancer, glioma, leukemia, medulloblastoma, melanoma, and prostate cancer [31].

Our study confirmed that Gli1 affects the apoptosis of HNSCC cells by regulating the transcription of BCL2 after translocating into the nucleus, consistent with the previous research indicating that BCL2 is a direct target of the SHH-Gli1 pathway [32, 33]. Moreover, anti-apoptotic BCL2 proteins promote hedgehog/Gli signaling by enhancing the turnover of a Gli antagonist, SUFU tumor suppressor to inhibit Gli-SUFU interaction, thus increasing the expression of Gli target genes such as BCL2 [34].

The pyridinyl imidazole compound SB-203580, commonly used as a p38 MAPK kinase inhibitor, has been widely studied in inflammation [35] and neoplasms [36]. However, several recent reports suggest that SB-203580 has some p38 MAPK-independent effects. It has been demonstrated that SB203580 elevated G-CSF expression in macrophages by enhancing the stability of G-CSF mRNA via its 3’UTR, without inhibiting the p38 MAPK activity [37]. SB-203580 also induced HCC cell autophagy independent of p38 MAPK [38] and inhibited the growth and migration ability of the human MDA-MB-231 cancer cell line through the inactivation of ERK1/2 phosphorylation [39]. Furthermore, non-specific inhibition of CK1 resulted in the inhibitory effect of SB-203580 on CREB phosphorylation. Moreover, DGIDB analysis indicated that SB-203580 may target many kinases, including CSNK1D. After demonstrating that CSNK1D (AA314-415) can promote the stable regulation of the GLI1-BCL2 axis in the CSNK1D-SHH-PTCH1 complex, we directly searched for CSNK1D-related drugs from DGIDB and differently expressed genes between CSNK1D high vs low group by dividing the TCGA HNSCC tumor patients based on the expression of CSNK1D-related drugs in L1000FWD. We identified three small molecule compounds: TAK-715, BMS-345541, and SB-203580, and determined their binding affinity with CSNK1D via molecular docking. SB-203580 had a smaller binding affinity and was selected for further research because the smaller the binding affinity, the more stable the binding [40]. However, whether SB-203580 can exert anti-cancer effects by inhibiting CSNK1D remains unclear.

In the present study, the IC50 of SB203580 was determined via the CCK8 assay, and the IC50 value was 17.10 μM in the HN4 cells and 14.78 μM in the CAL27 cells. However, the IC50 value of SB203580 was 85.1 μM in MDA-MB-231 cells [38], four times larger than that reported in our study, indicating that SB-203580 may be more effective in HNSCC cell lines. As presented in the current study, SB-203580 inhibited the proliferation, migration and invasion but promoted the apoptosis of HNSCC cell lines, in line with previous research findings [41]. To address whether SB-203580 influences CSNK1D activity, we conducted immunoprecipitation and Western blot experiments demonstrating that SB-203580 can affect the binding of CSNK1D to SHH-PTCH1 complex without affecting CSNK1D expression. To the best of our knowledge, this is the first report of SB-203580 inhibiting CSNK1D as a therapeutic target for HNSCC. In general, SB-203580 was characterized as a small CSNK1D-targeting molecule, suggesting that SB-203580 may not be limited to its known target P38. The presented data highlights SB-203580 as a potential hit for the development of novel and effective CSNK1D inhibitors.

In conclusion, our findings illustrate that CSNK1D regulates the stability of the CSNK1D-SHH-PTCH1 complex to control the Gli1-BCL2 axis, promoting the development of HNSCC. SB-203580 is a potential inhibitor of CSNK1D, which offering new perspectives for the clinical treatment of HNSCC.

## Supplementary information

Supplemental Tables

Supplemental Figures

Full original pictures

## Data Availability

All data and materials generated and analyzed during the present study are available from the corresponding author on reasonable requests.
