# Supplementary material for: CSNK1D inhibition suppresses head and neck squamous cell carcinoma progression through SHH and PTCH1 pathway
Source: Cell Death Dis. 2025 Dec 6;17(1):3. doi: 10.1038/s41419-025-08276-7 (PMC12779978; doi:10.1038/s41419-025-08276-7)
Supplement: Supplementary file 2 — Supplemental Figures [file 41419_2025_8276_MOESM2_ESM.pdf]

Supplemental Figures

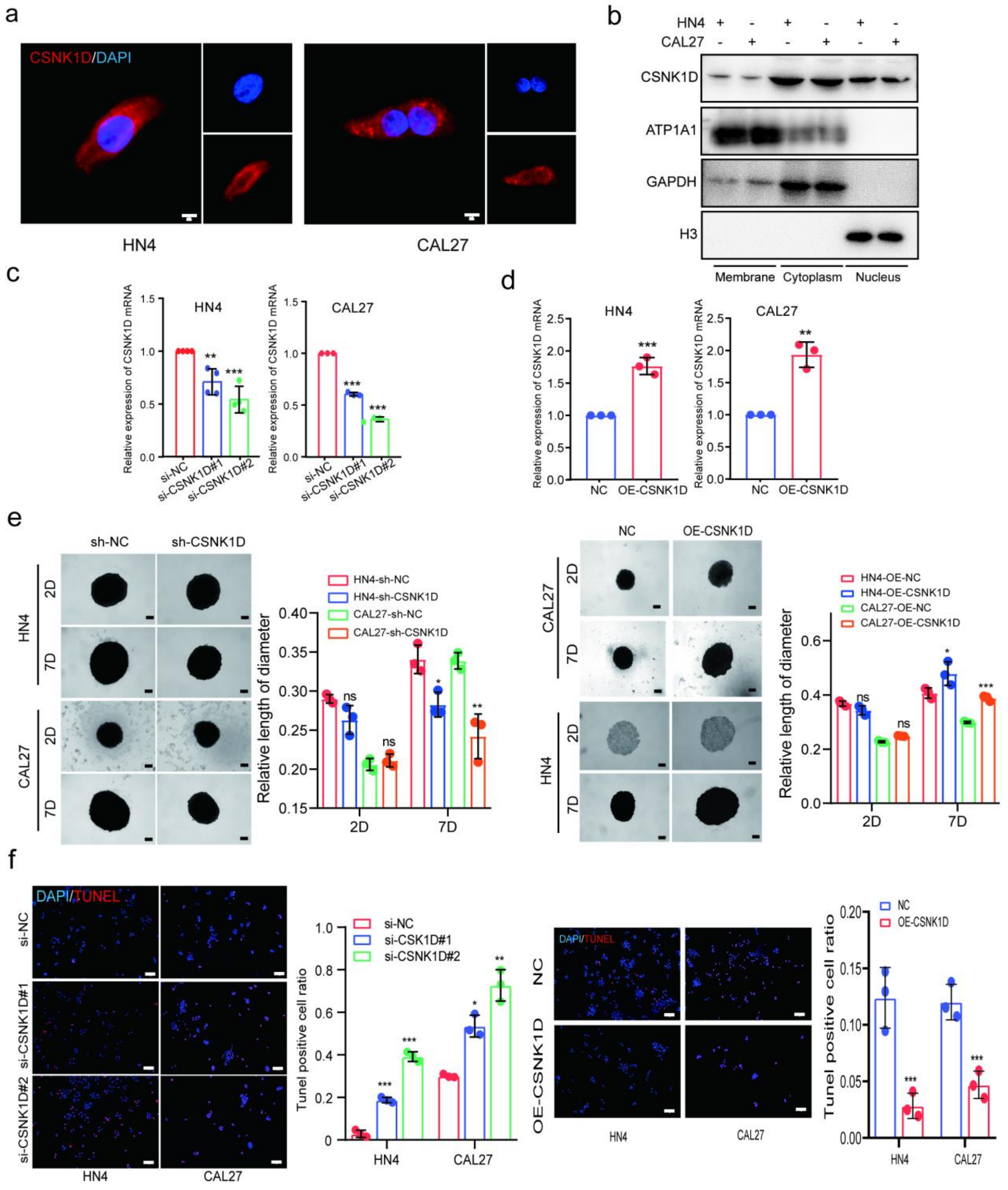

Figure S1. (A) Representative immunofluorescence images showing the cellular localization of CSNK1D. The CSNK1D was labeled with Cy3 (red), and nuclei were stained with DAPI (blue). (scale bar,25μm). (B) A subcellular fractionation assay indicating that CSNK1D is mainly located in the cytoplasm, with a small proportion in the nucleus and membrane. (C) qRT-PCR analysis of CSNK1D mRNA levels assessed in HN4 and CAL27 cells treated with si-CSNK1D. (D) qRT-PCR analysis of CSNK1D mRNA levels assessed in HN4 and CAL27 cells treated with oe-CSNK1D.(E)

Spheroid proliferation assays showing reduced cell proliferation of the shCSNK1D knockdown CAL27 and HN4 cells(scale bar,200μm). (F) Tunel assay of si-CSNK1D or oe-CSNK1D and corresponding control HN4 and CAL27 cells (scale bar,100μm).

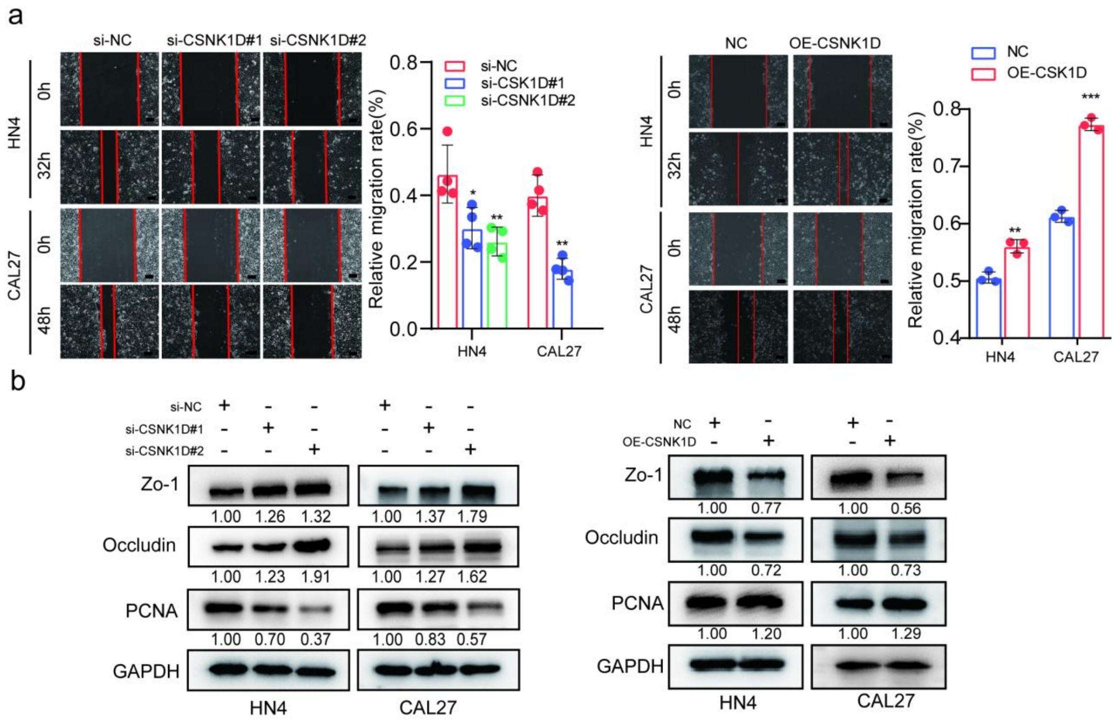

Figure S2. (A) The migration capability was measured in HN4 and CAL27 cells treated with si-CSNK1D or oe-CSNK1D, as determined using the wound-healing assay(scale bar,100μm). (B) Western blot analysis of Zo-1, Occludin, and PCNA proteins in HN4 and CAL27 cells after CSNK1D-knockdown or CSNK1D overexpression. GAPDH was used as the loading control.

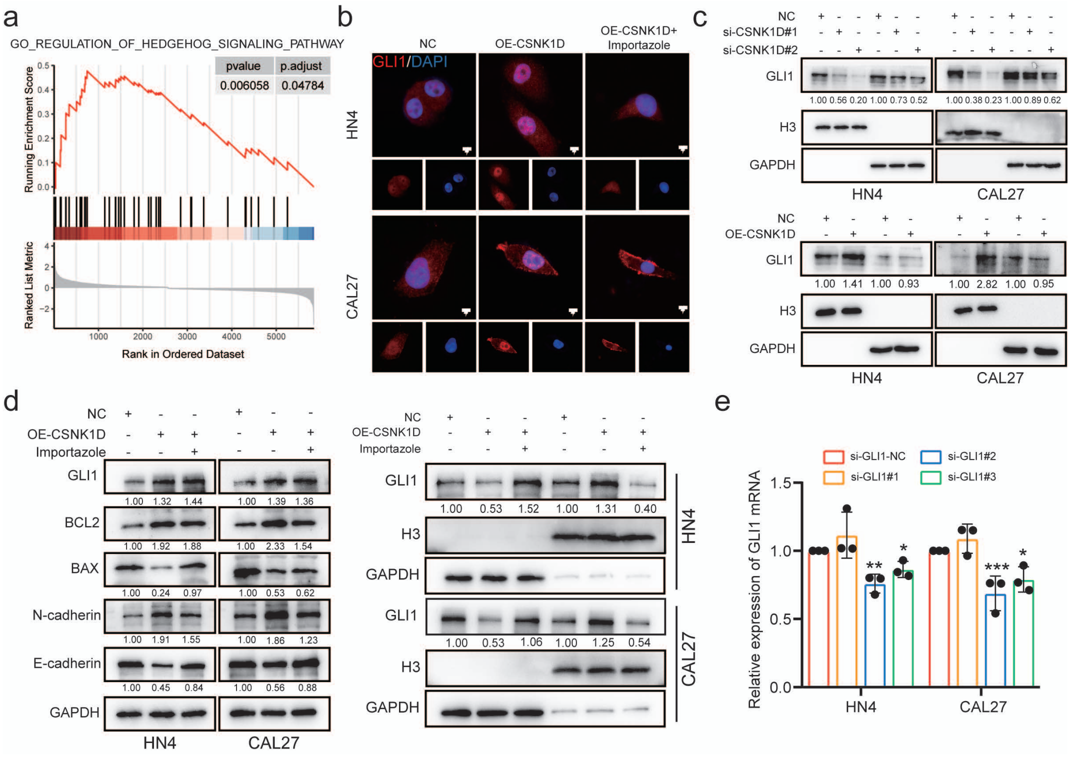

Figure S3. (A) Gene Set Enrichment Analysis of CSNK1D high vs CSNK1D low groups in TCGA datasets. (B) The distribution of GLI1 in HN4 and CAL27 cells treated with importazole (nuclear transport inhibitor) after CSNK1D overexpression(scale bar,25μm). (C) The nuclear and cytoplasmic distribution of GLI1 after CSNK1D silencing or overexpression detected via Western blot. (D) Protein expression of Gli1, Bcl2, Bax, N-cadherin, and E-cadherin and distribution of GLI1 in the nucleus and cytoplasm of HN4 and CAL27 cells treated with importazole (nuclear transport inhibitor) after CSNK1D overexpression. (E) GLI1 mRNA levels were assessed by qRT-PCR in HN4 and CAL27 cells treated with si-GLI1.

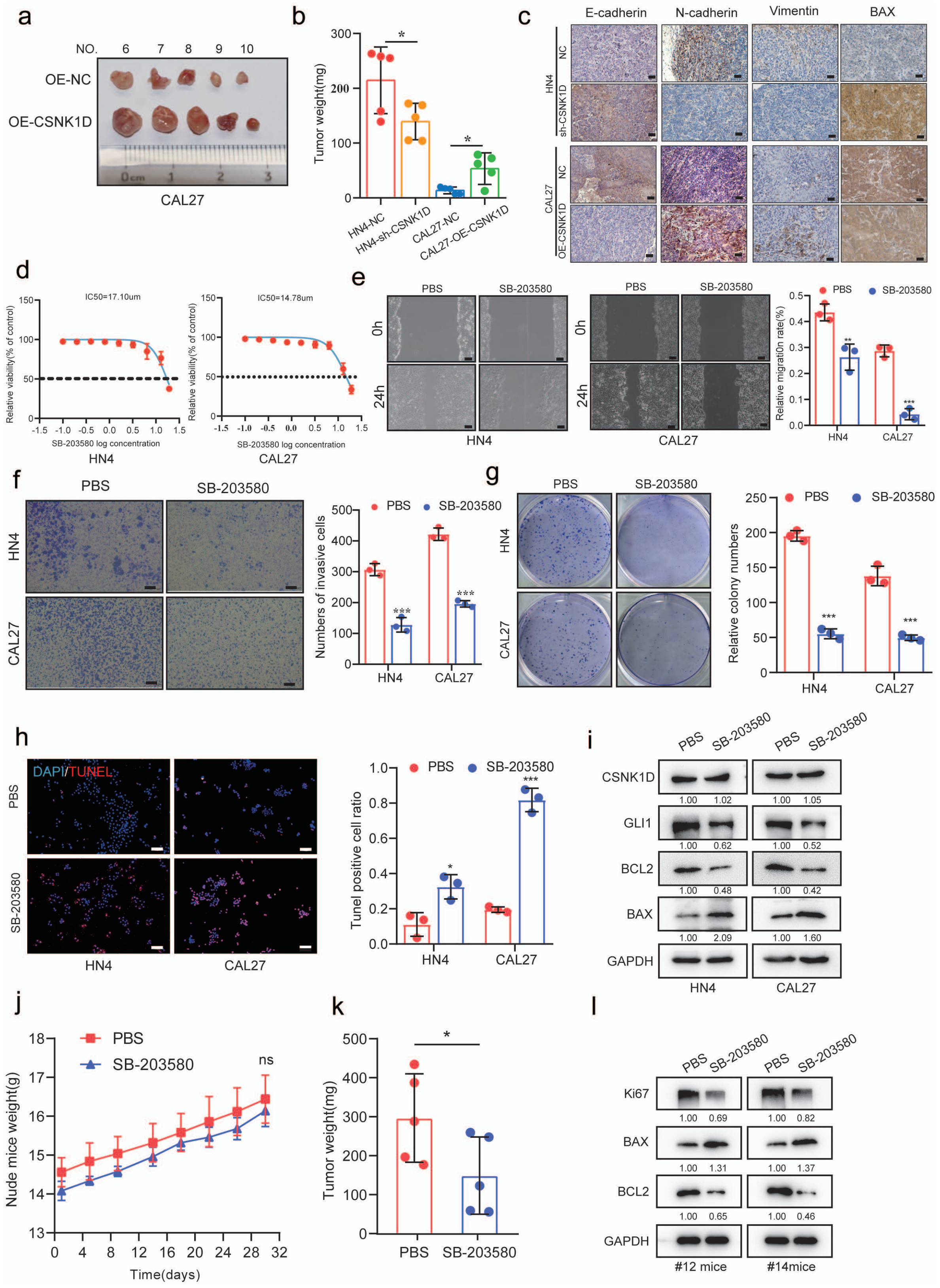

Figure S4. (A) Images of tumors in the CAL27-NC and CAL27-oe-CSNK1D cells in the subcutaneous tissues. (B) Weight of tumors in the HN4-NC and HN4-sh-CSNK1D cells or CAL27-NC and CAL27-oe-CSNK1D cells in the subcutaneous tissues at the end of the experiment. (C)

Representative IHC staining images of E-cadherin, N-cadherin, vimentin, and bax in mouse orthotopic tumor tissues (scale bar,100μm). (D) IC50 of SB-203580 in HN4 and CAL27 cells, as determined by CCK8 assay. (E) The wound-healing assay showing the SB-203580-mediated suppression of the migration capability of HN4 and CAL27 cells(scale bar,100μm). (F)Transwell assay showing SB-203580-mediated inhibition of the invasion ability of HN4 and CAL27 cells(scale bar,200μm). (G) Colony formation and quantification analysis of SB-203580 treatment in HN4 and CAL27 cells. (H) Apoptosis of HN4 and CAL27 cells treated with SB-203580, as shown by Tunel assay(scale bar,100μm). (I) Detection of CSNK1D, gli1, bcl2, and bax proteins in HN4 and CAL27 cells treated with SB-203580. (J) Evaluation of mouse body weights every four days for two weeks after tumour cell injection. (K) Weight of tumors in the HN4 cells treated with phosphate-buffered saline (PBS) or SB-203580 in the subcutaneous tissues at the end of the experiment. (L) Protein expression of KI67, BAX and Bcl2 protein in HN4 cells treated with PBS or SB-203580 in the subcutaneous mouse orthotopic tumor tissues.
