# Supplementary material for: CSNK1D inhibition suppresses head and neck squamous cell carcinoma progression through SHH and PTCH1 pathway
Source: Cell Death Dis. 2025 Dec 6;17(1):3. doi: 10.1038/s41419-025-08276-7 (PMC12779978; doi:10.1038/s41419-025-08276-7)

Full original pictures

Fig.2A

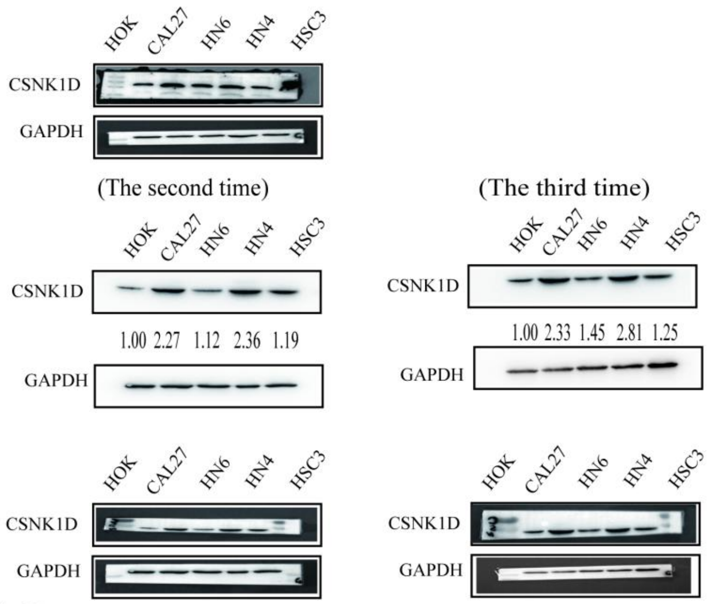

Fig.2B

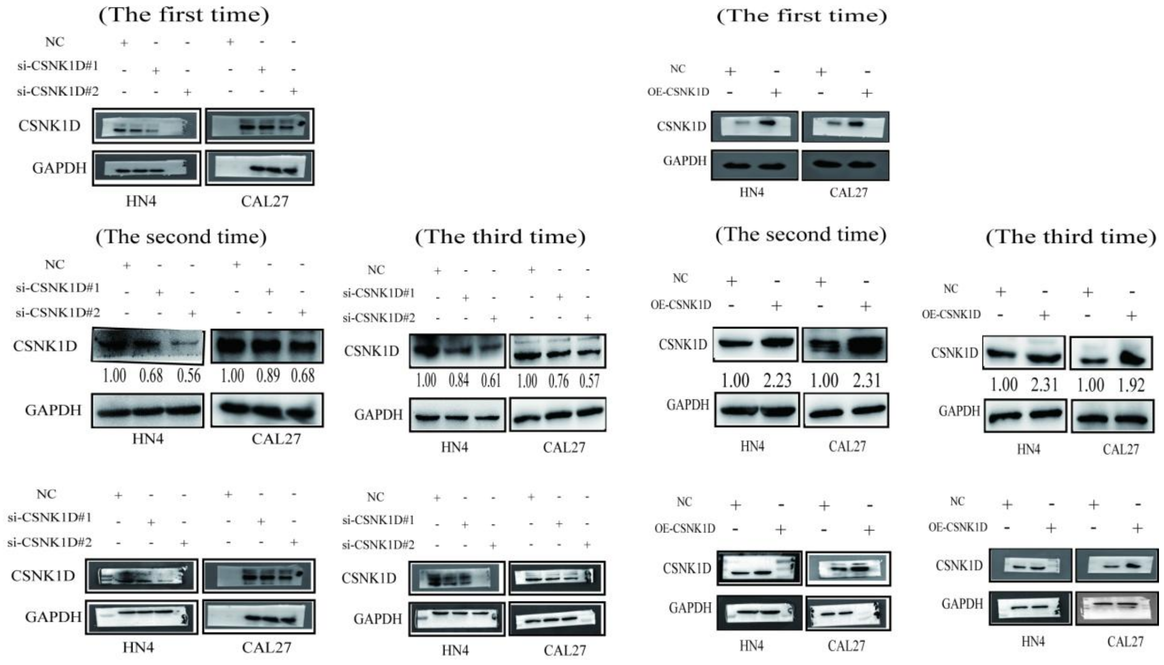

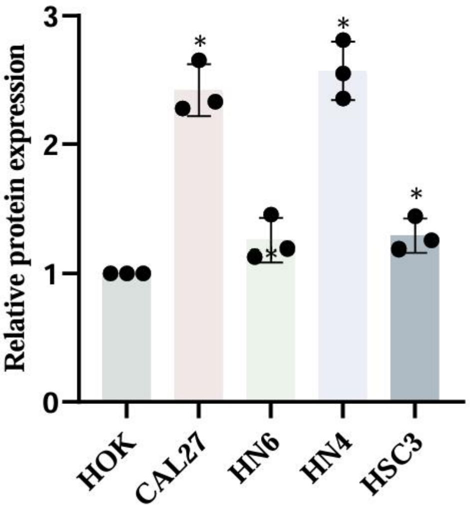
Relative protein expression
3
2
1
0
*
*
*
*
HOK
CAL27
HN6
HN4
HSC3

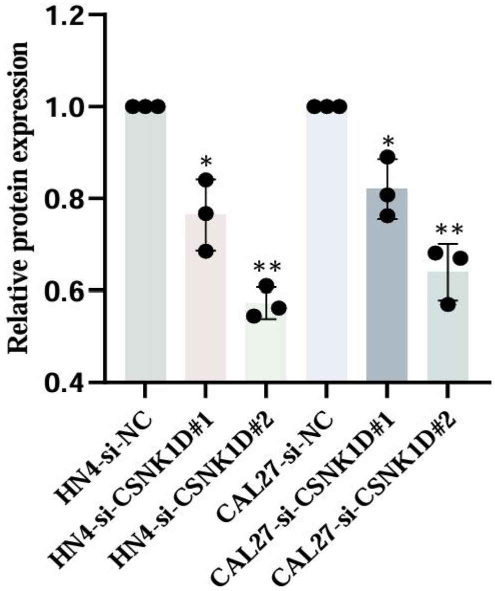
Relative protein expression
1.2
1.0
0.8
0.6
0.4
*
**
*
**
HN4-si-NC
HN4-si-CSNK1D#1
HN4-si-CSNK1D#2
CAL27-si-NC
CAL27-si-CSNK1D#1
CAL27-si-CSNK1D#2

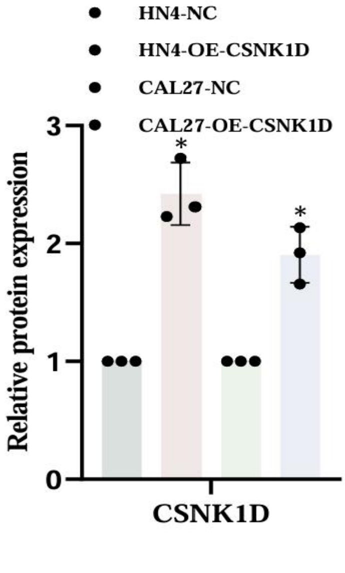
HN4-NC
HN4-OE-CSNK1D
CAL27-NC
CAL27-OE-CSNK1D
Relative protein expression
3
2
1
0
*
*
CSNK1D

Fig.3C

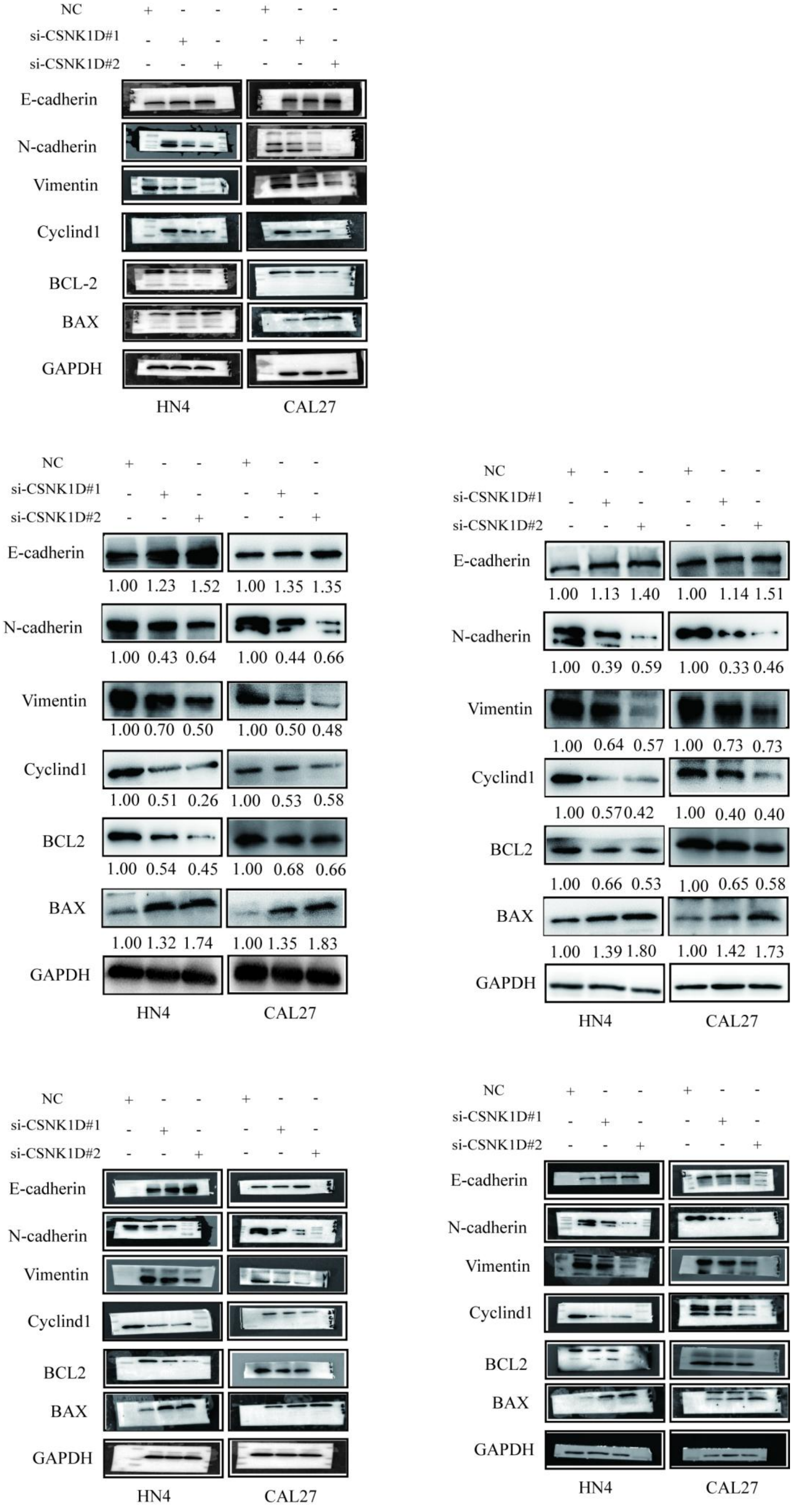
NC
si-CSNK1D#1
si-CSNK1D#2
E-cadherin
N-cadherin
Vimentin
Cyclind1
BCL-2
BAX
GAPDH
HN4
CAL27
NC
si-CSNK1D#1
si-CSNK1D#2
E-cadherin
1.00 1.23 1.52 1.00 1.35 1.35
N-cadherin
1.00 0.43 0.64 1.00 0.44 0.66
Vimentin
1.00 0.70 0.50 1.00 0.50 0.48
Cyclind1
1.00 0.51 0.26 1.00 0.53 0.58
BCL2
1.00 0.54 0.45 1.00 0.68 0.66
BAX
1.00 1.32 1.74 1.00 1.35 1.83
GAPDH
HN4
CAL27
NC
si-CSNK1D#1
si-CSNK1D#2
E-cadherin
1.00 1.13 1.40 1.00 1.14 1.51
N-cadherin
1.00 0.39 0.59 1.00 0.33 0.46
Vimentin
1.00 0.64 0.57 1.00 0.73 0.73
Cyclind1
1.00 0.570.42 1.00 0.40 0.40
BCL2
1.00 0.66 0.53 1.00 0.65 0.58
BAX
1.00 1.39 1.80 1.00 1.42 1.73
GAPDH
HN4
CAL27
NC
si-CSNK1D#1
si-CSNK1D#2
E-cadherin
N-cadherin
Vimentin
Cyclind1
BCL2
BAX
GAPDH
HN4
CAL27
NC
si-CSNK1D#1
si-CSNK1D#2
E-cadherin
N-cadherin
Vimentin
Cyclind1
BCL2
BAX
GAPDH
HN4
CAL27

Fig.3C

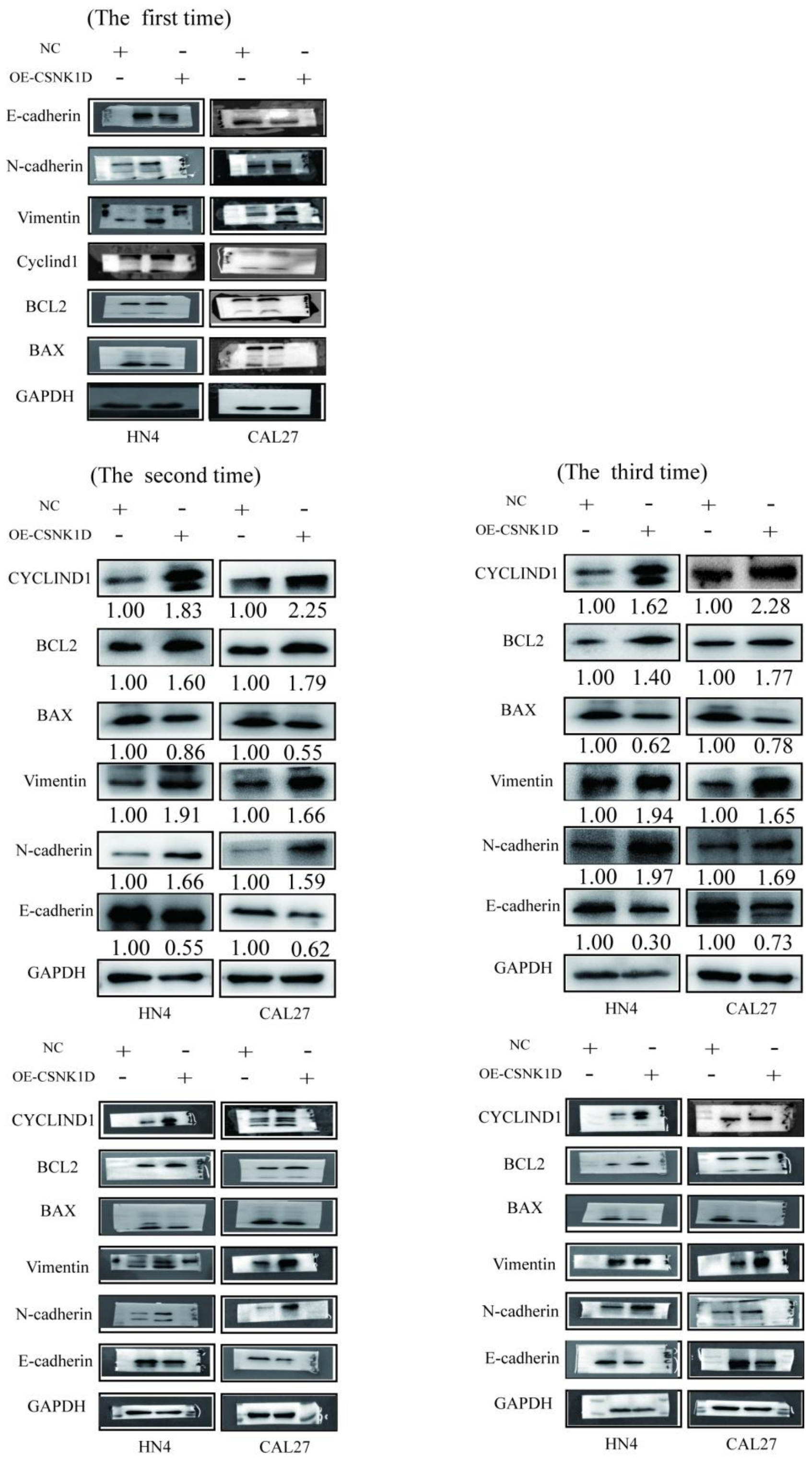
(The first time)
NC + - + -
OE-CSNK1D - + - +
E-cadherin
N-cadherin
Vimentin
Cyclind1
BCL2
BAX
GAPDH
HN4
CAL27
(The second time)
NC + - + -
OE-CSNK1D - + - +
CYCLIND1
1.00 1.83 1.00 2.25
BCL2
1.00 1.60 1.00 1.79
BAX
1.00 0.86 1.00 0.55
Vimentin
1.00 1.91 1.00 1.66
N-cadherin
1.00 1.66 1.00 1.59
E-cadherin
1.00 0.55 1.00 0.62
GAPDH
HN4
CAL27
(The third time)
NC + - + -
OE-CSNK1D - + - +
CYCLIND1
1.00 1.62 1.00 2.28
BCL2
1.00 1.40 1.00 1.77
BAX
1.00 0.62 1.00 0.78
Vimentin
1.00 1.94 1.00 1.65
N-cadherin
1.00 1.97 1.00 1.69
E-cadherin
1.00 0.30 1.00 0.73
GAPDH
HN4
CAL27
NC + - + -
OE-CSNK1D - + - +
CYCLIND1
BCL2
BAX
Vimentin
N-cadherin
E-cadherin
GAPDH
HN4
CAL27
NC + - + -
OE-CSNK1D - + - +
CYCLIND1
BCL2
BAX
Vimentin
N-cadherin
E-cadherin
GAPDH
HN4
CAL27

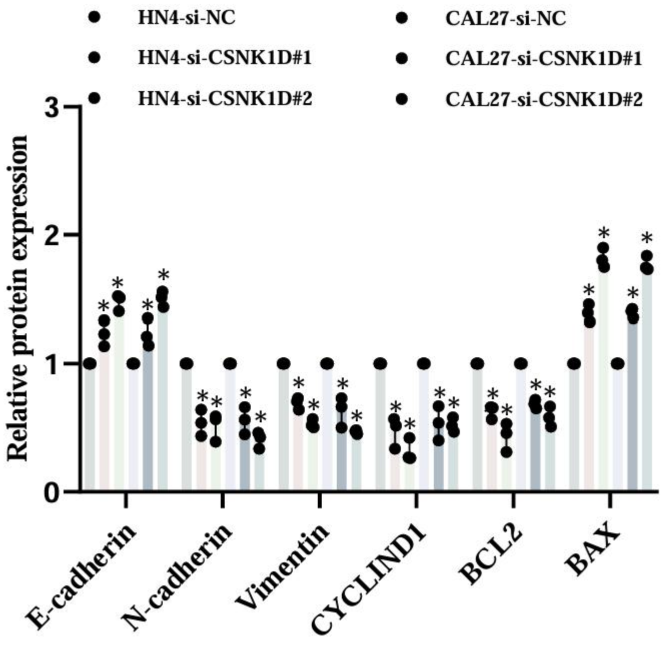
HN4-si-NC
HN4-si-CSNK1D#1
HN4-si-CSNK1D#2
CAL27-si-NC
CAL27-si-CSNK1D#1
CAL27-si-CSNK1D#2
Relative protein expression
3
2
1
0
E-cadherin
N-cadherin
Vimentin
CYCLIND1
BCL2
BAX

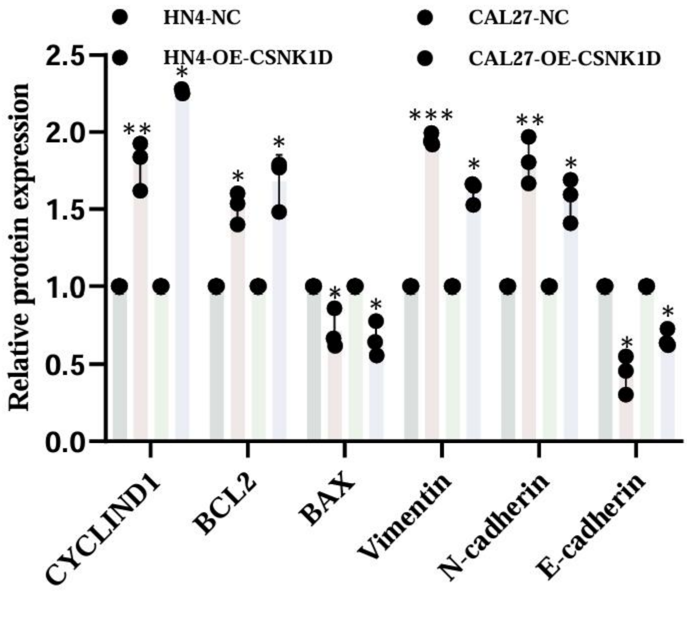
HN4-NC
HN4-OE-CSNK1D
CAL27-NC
CAL27-OE-CSNK1D
Relative protein expression
2.5
2.0
1.5
1.0
0.5
0.0
CYCLIND1
BCL2
BAX
Vimentin
N-cadherin
E-cadherin

Fig.4C

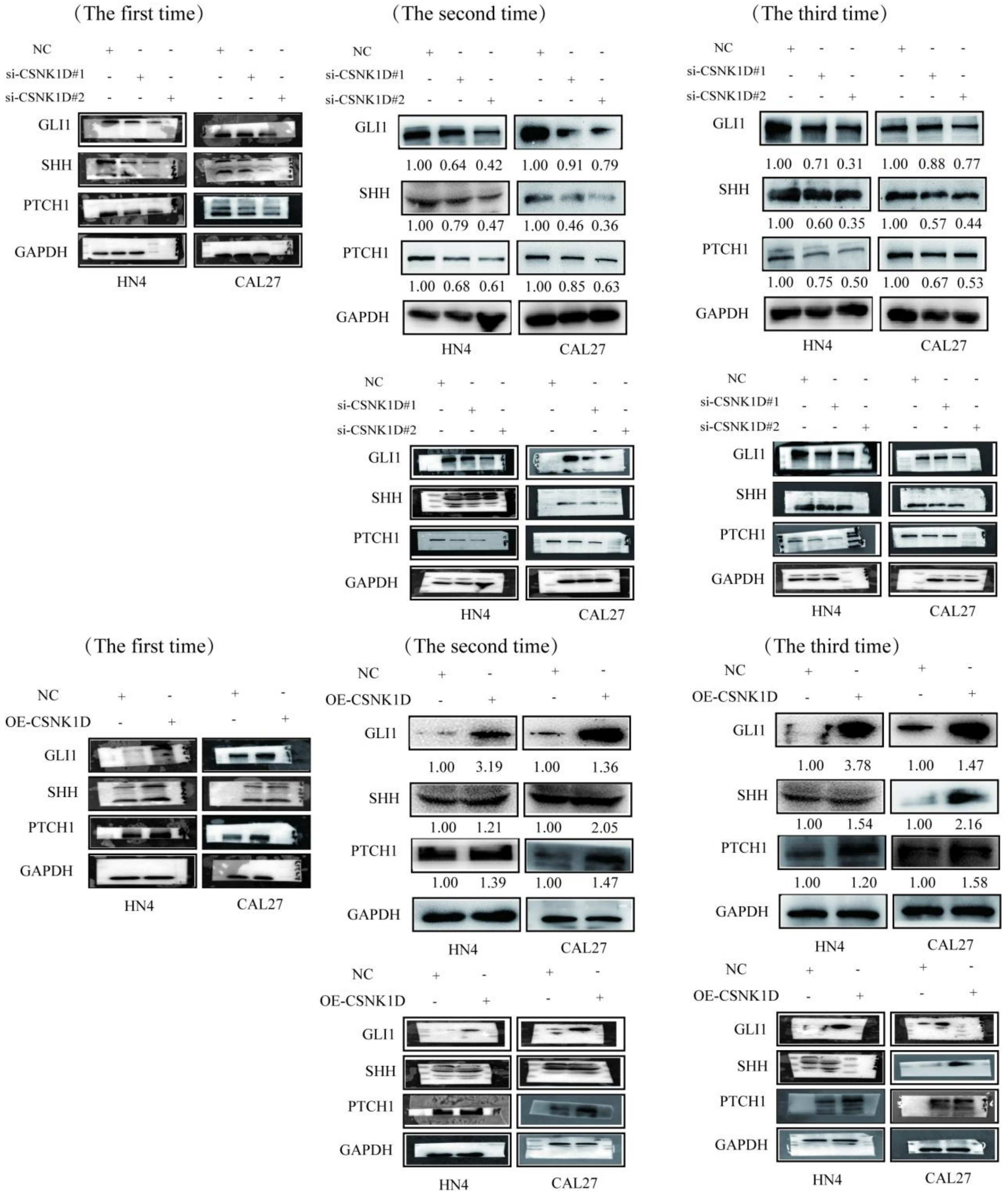

(The first time)
NC
si-CSNK1D#1
si-CSNK1D#2
GLI1
SHH
PTCH1
GAPDH
HN4
CAL27
(The second time)
NC
si-CSNK1D#1
si-CSNK1D#2
GLI1
1.00 0.64 0.42 1.00 0.91 0.79
SHH
1.00 0.79 0.47 1.00 0.46 0.36
PTCH1
1.00 0.68 0.61 1.00 0.85 0.63
GAPDH
HN4
CAL27
(The third time)
NC
si-CSNK1D#1
si-CSNK1D#2
GLI1
1.00 0.71 0.31 1.00 0.88 0.77
SHH
1.00 0.60 0.35 1.00 0.57 0.44
PTCH1
1.00 0.75 0.50 1.00 0.67 0.53
GAPDH
HN4
CAL27
NC
si-CSNK1D#1
si-CSNK1D#2
GLI1
SHH
PTCH1
GAPDH
HN4
CAL27
NC
si-CSNK1D#1
si-CSNK1D#2
GLI1
SHH
PTCH1
GAPDH
HN4
CAL27
(The first time)
NC
OE-CSNK1D
GLI1
SHH
PTCH1
GAPDH
HN4
CAL27
(The second time)
NC
OE-CSNK1D
GLI1
1.00 3.19 1.00 1.36
SHH
1.00 1.21 1.00 2.05
PTCH1
1.00 1.39 1.00 1.47
GAPDH
HN4
CAL27
(The third time)
NC
OE-CSNK1D
GLI1
1.00 3.78 1.00 1.47
SHH
1.00 1.54 1.00 2.16
PTCH1
1.00 1.20 1.00 1.58
GAPDH
HN4
CAL27
NC
OE-CSNK1D
GLI1
SHH
PTCH1
GAPDH
HN4
CAL27
NC
OE-CSNK1D
GLI1
SHH
PTCH1
GAPDH
HN4
CAL27

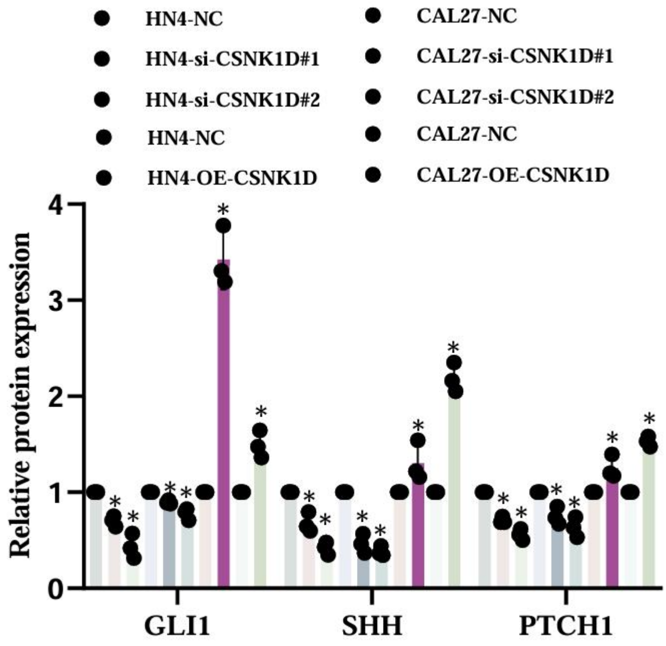

HN4-NC
HN4-si-CSNK1D#1
HN4-si-CSNK1D#2
HN4-NC
HN4-OE-CSNK1D
CAL27-NC
CAL27-si-CSNK1D#1
CAL27-si-CSNK1D#2
CAL27-NC
CAL27-OE-CSNK1D
Relative protein expression
0
1
2
3
4
GLI1
SHH
PTCH1

Fig.4D

(The first time)

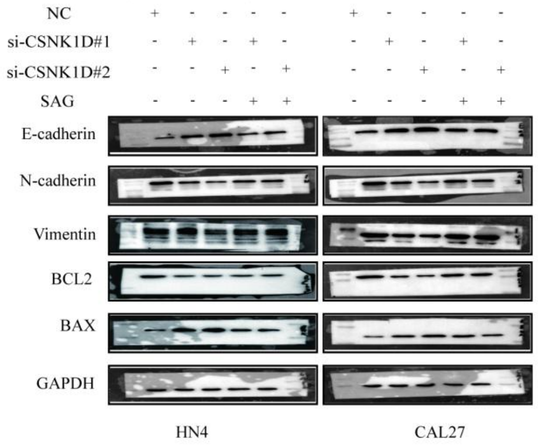

(The second time)

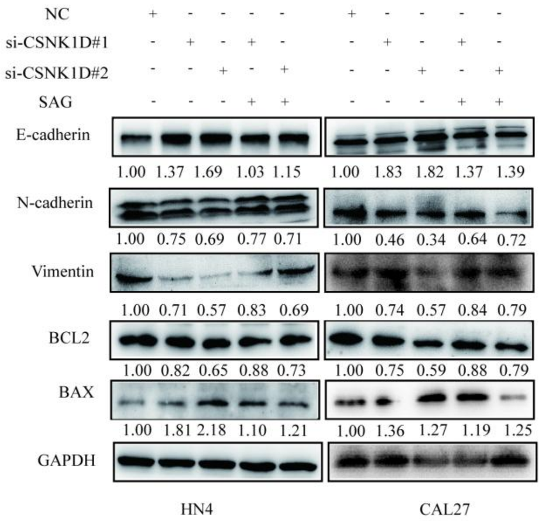

(The third time)

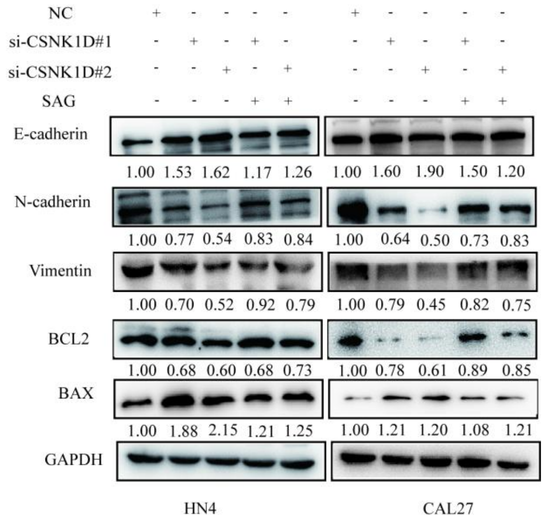

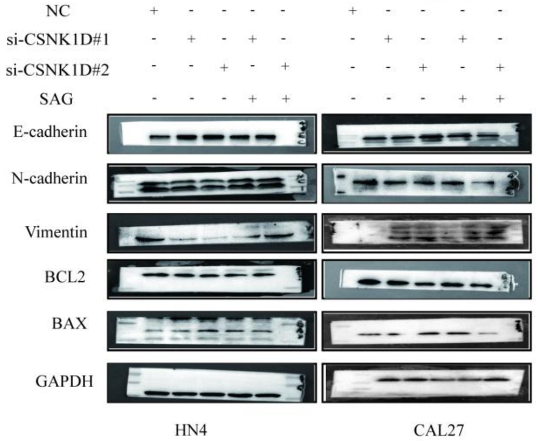

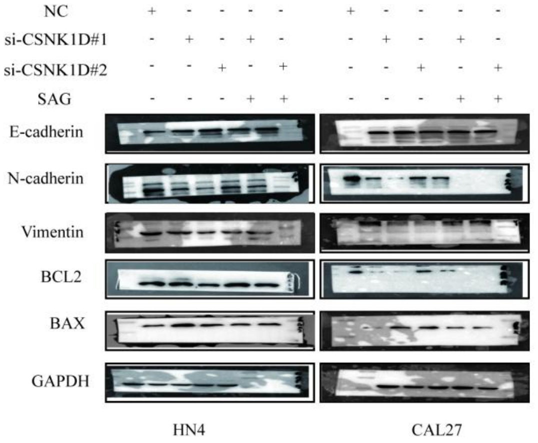

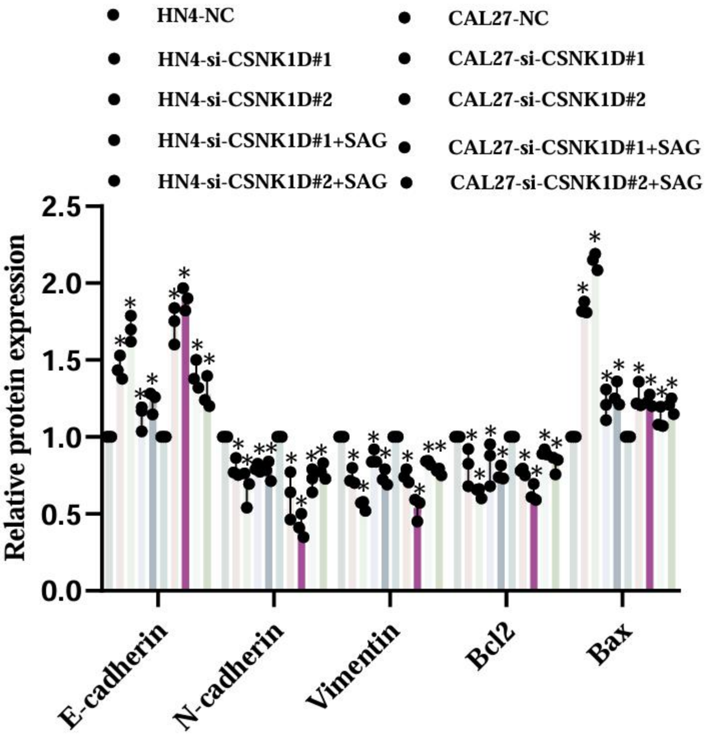

HN4-NC
HN4-si-CSNK1D#1
HN4-si-CSNK1D#2
HN4-si-CSNK1D#1+SAG
HN4-si-CSNK1D#2+SAG
CAL27-NC
CAL27-si-CSNK1D#1
CAL27-si-CSNK1D#2
CAL27-si-CSNK1D#1+SAG
CAL27-si-CSNK1D#2+SAG
Relative protein expression
2.5
2.0
1.5
1.0
0.5
0.0
E-cadherin
N-cadherin
Vimentin
Bcl2
Bax

Fig.4D

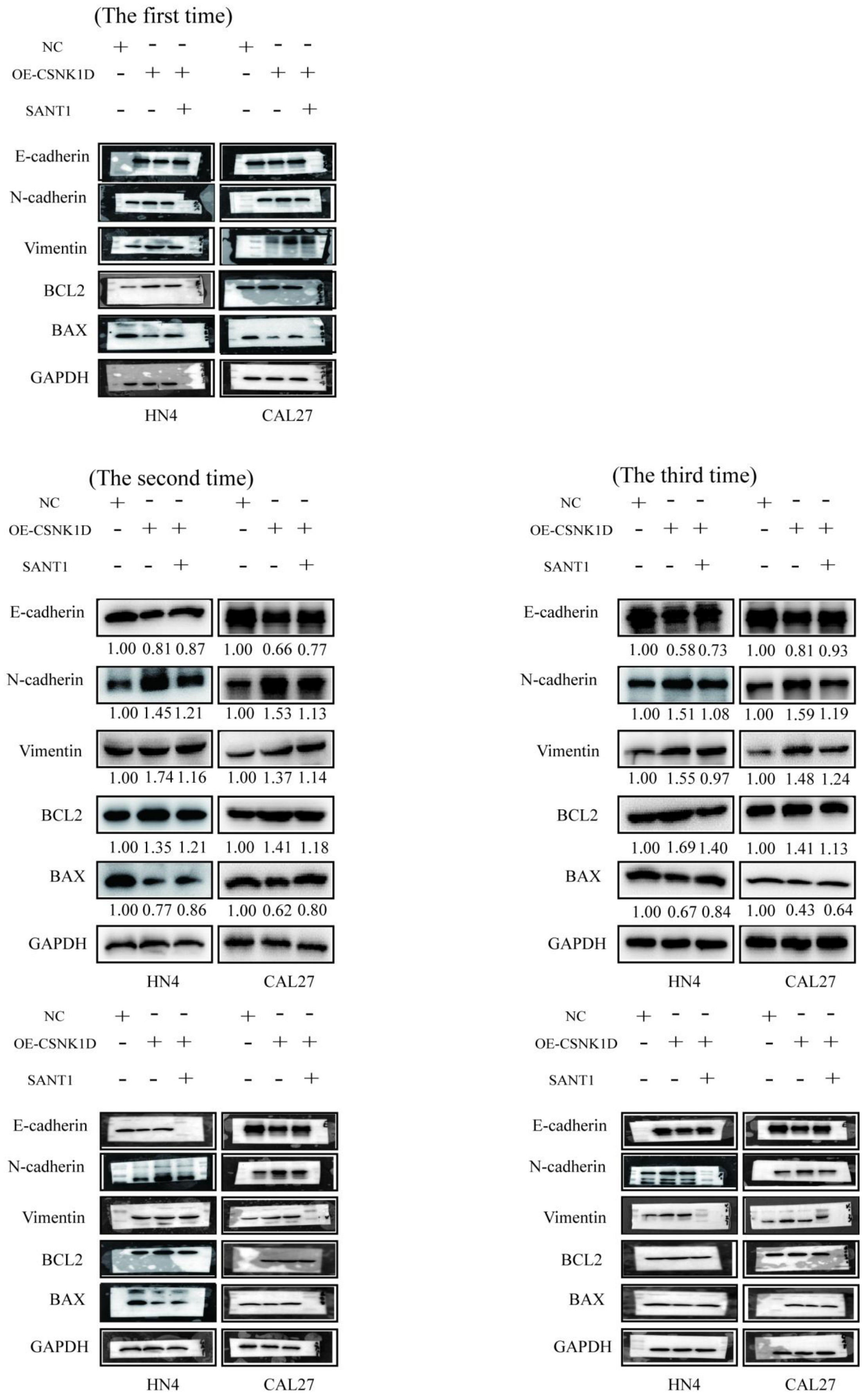
(The first time)
NC + - - + - -
OE-CSNK1D - + + - + +
SANT1 - - + - - +
E-cadherin
N-cadherin
Vimentin
BCL2
BAX
GAPDH
HN4
CAL27
(The second time)
NC + - - + - -
OE-CSNK1D - + + - + +
SANT1 - - + - - +
E-cadherin
1.00 0.81 0.87 1.00 0.66 0.77
N-cadherin
1.00 1.45 1.21 1.00 1.53 1.13
Vimentin
1.00 1.74 1.16 1.00 1.37 1.14
BCL2
1.00 1.35 1.21 1.00 1.41 1.18
BAX
1.00 0.77 0.86 1.00 0.62 0.80
GAPDH
HN4
CAL27
(The third time)
NC + - - + - -
OE-CSNK1D - + + - + +
SANT1 - - + - - +
E-cadherin
1.00 0.58 0.73 1.00 0.81 0.93
N-cadherin
1.00 1.51 1.08 1.00 1.59 1.19
Vimentin
1.00 1.55 0.97 1.00 1.48 1.24
BCL2
1.00 1.69 1.40 1.00 1.41 1.13
BAX
1.00 0.67 0.84 1.00 0.43 0.64
GAPDH
HN4
CAL27
NC + - - + - -
OE-CSNK1D - + + - + +
SANT1 - - + - - +
E-cadherin
N-cadherin
Vimentin
BCL2
BAX
GAPDH
HN4
CAL27
NC + - - + - -
OE-CSNK1D - + + - + +
SANT1 - - + - - +
E-cadherin
N-cadherin
Vimentin
BCL2
BAX
GAPDH
HN4
CAL27

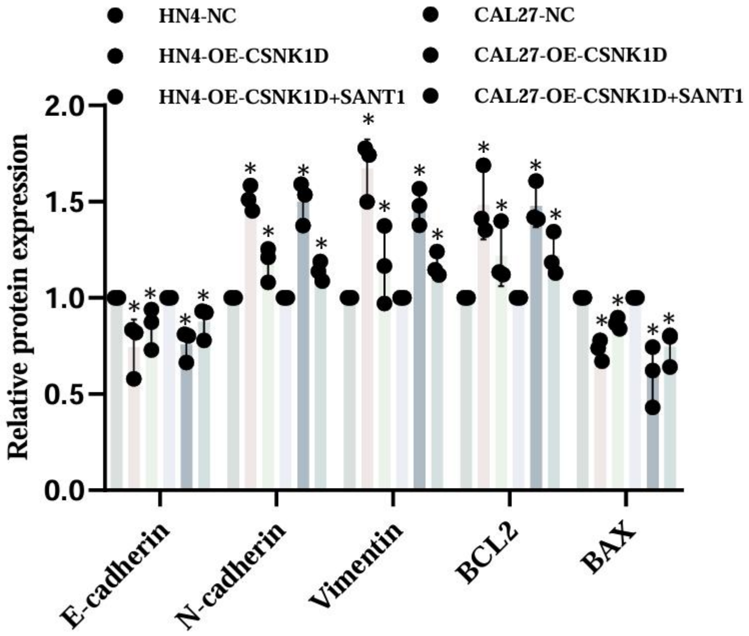
HN4-NC
HN4-OE-CSNK1D
HN4-OE-CSNK1D+SANT1
CAL27-NC
CAL27-OE-CSNK1D
CAL27-OE-CSNK1D+SANT1
Relative protein expression
2.0
1.5
1.0
0.5
0.0
E-cadherin
N-cadherin
Vimentin
BCL2
BAX

Fig.4F

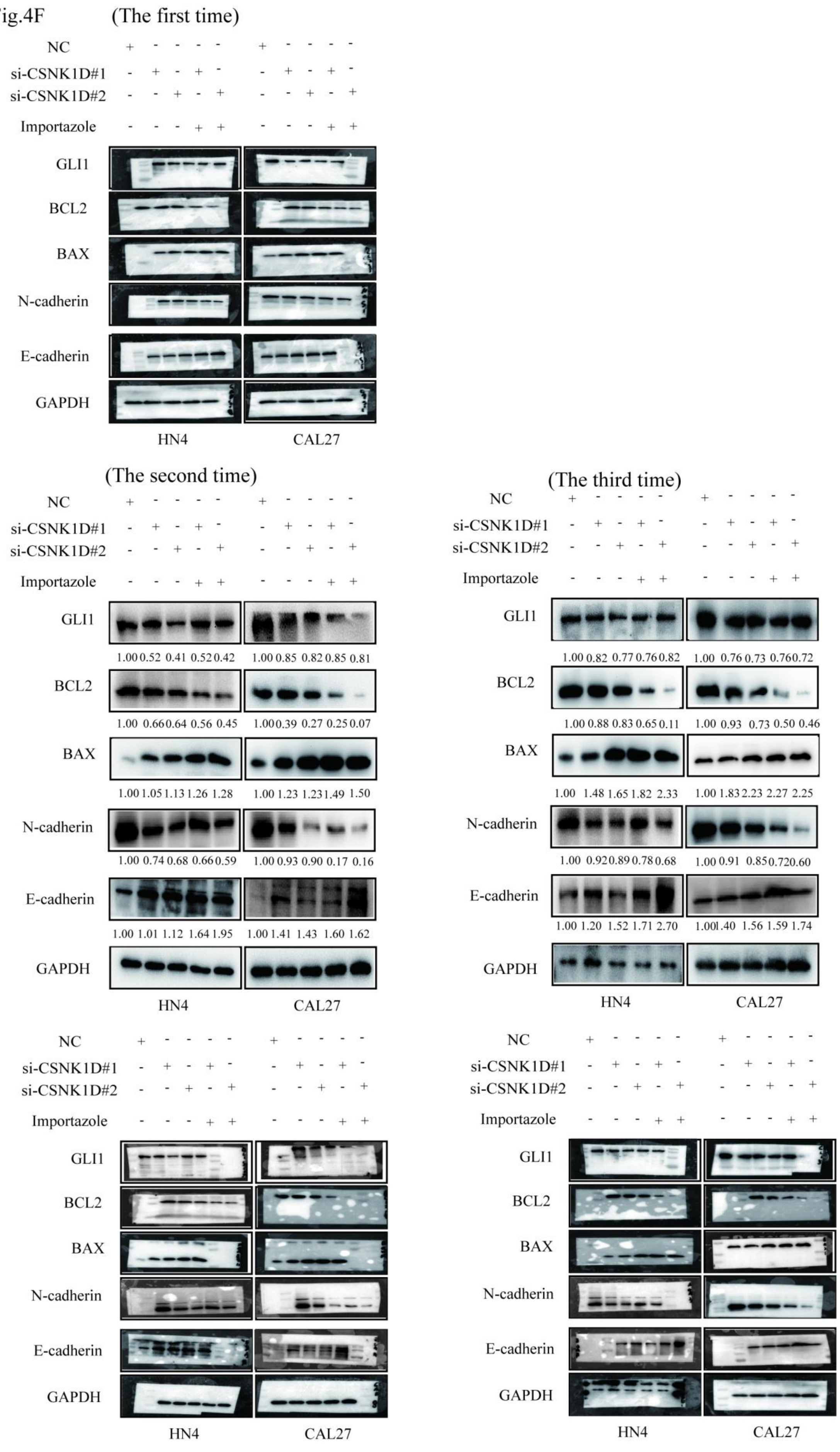
(The first time)
NC
si-CSNK1D#1
si-CSNK1D#2
Importazole
GLI1
BCL2
BAX
N-cadherin
E-cadherin
GAPDH
HN4
CAL27
(The second time)
NC
si-CSNK1D#1
si-CSNK1D#2
Importazole
GLI1
1.00 0.52 0.41 0.52 0.42
1.00 0.85 0.82 0.85 0.81
BCL2
1.00 0.66 0.64 0.56 0.45
1.00 0.39 0.27 0.25 0.07
BAX
1.00 1.05 1.13 1.26 1.28
1.00 1.23 1.23 1.49 1.50
N-cadherin
1.00 0.74 0.68 0.66 0.59
1.00 0.93 0.90 0.17 0.16
E-cadherin
1.00 1.01 1.12 1.64 1.95
1.00 1.41 1.43 1.60 1.62
GAPDH
HN4
CAL27
(The third time)
NC
si-CSNK1D#1
si-CSNK1D#2
Importazole
GLI1
1.00 0.82 0.77 0.76 0.82
1.00 0.76 0.73 0.76 0.72
BCL2
1.00 0.88 0.83 0.65 0.11
1.00 0.93 0.73 0.50 0.46
BAX
1.00 1.48 1.65 1.82 2.33
1.00 1.83 2.23 2.27 2.25
N-cadherin
1.00 0.92 0.89 0.78 0.68
1.00 0.91 0.85 0.72 0.60
E-cadherin
1.00 1.20 1.52 1.71 2.70
1.00 1.40 1.56 1.59 1.74
GAPDH
HN4
CAL27
NC
si-CSNK1D#1
si-CSNK1D#2
Importazole
GLI1
BCL2
BAX
N-cadherin
E-cadherin
GAPDH
HN4
CAL27
NC
si-CSNK1D#1
si-CSNK1D#2
Importazole
GLI1
BCL2
BAX
N-cadherin
E-cadherin
GAPDH
HN4
CAL27

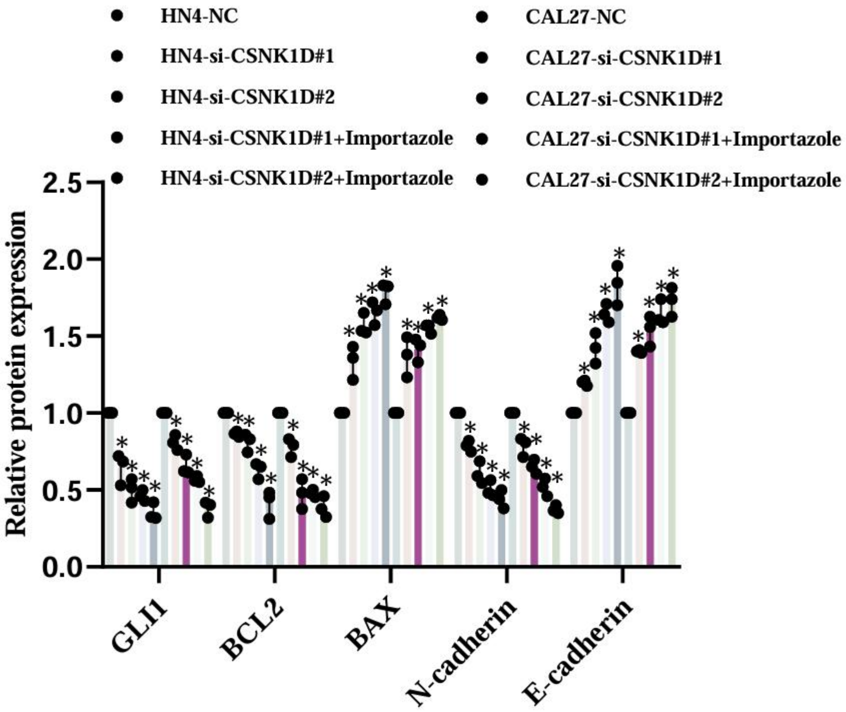

HN4-NC
HN4-si-CSNK1D#1
HN4-si-CSNK1D#2
HN4-si-CSNK1D#1+Importazole
HN4-si-CSNK1D#2+Importazole
CAL27-NC
CAL27-si-CSNK1D#1
CAL27-si-CSNK1D#2
CAL27-si-CSNK1D#1+Importazole
CAL27-si-CSNK1D#2+Importazole
Relative protein expression
2.5
2.0
1.5
1.0
0.5
0.0
GLI1
BCL2
BAX
N-cadherin
E-cadherin

Fig.4F

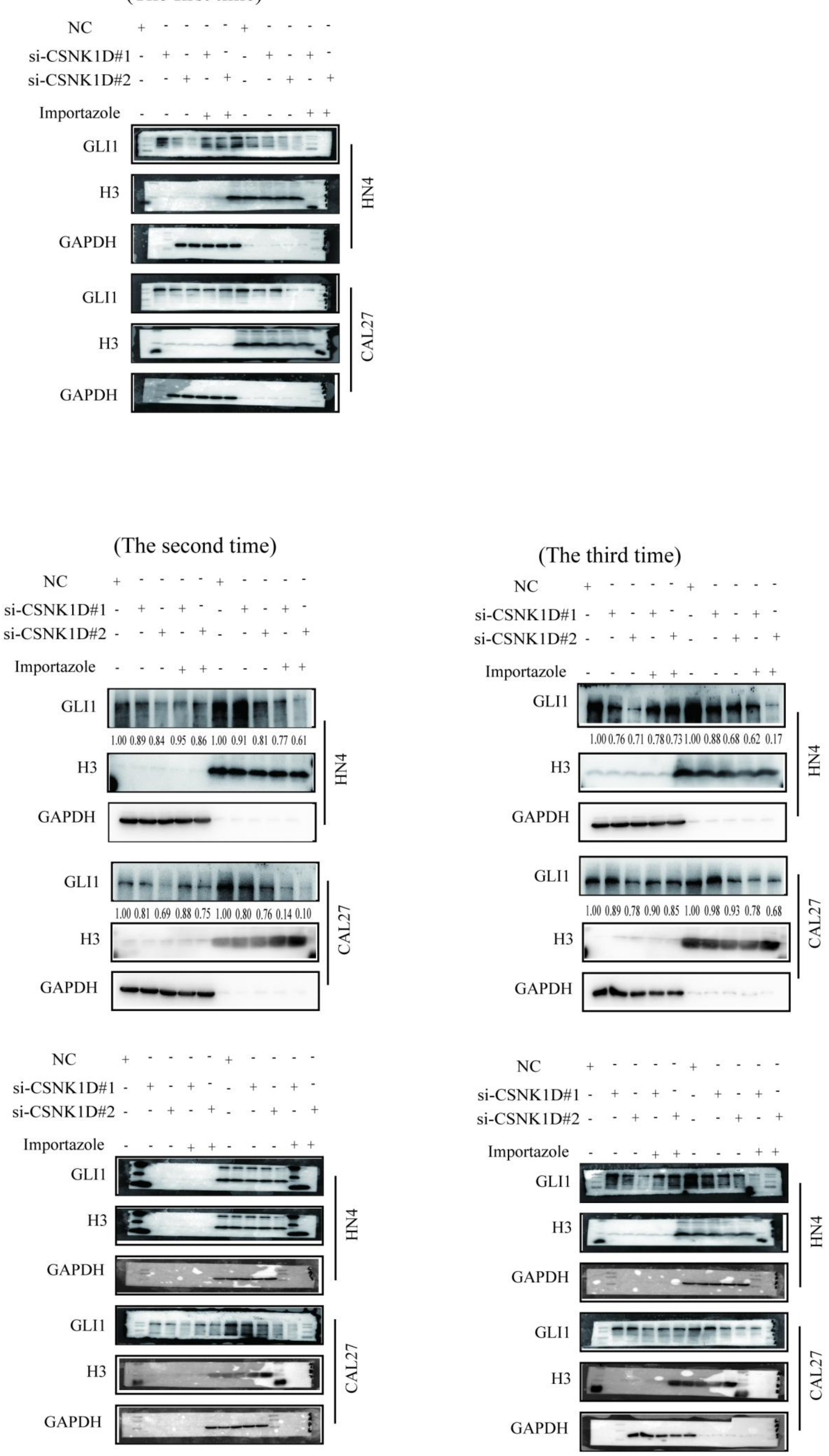
(The first time)
NC
si-CSNK1D#1
si-CSNK1D#2
Importazole
GLI1
H3
GAPDH
HN4
GLI1
H3
GAPDH
CAL27
(The second time)
NC
si-CSNK1D#1
si-CSNK1D#2
Importazole
GLI1
1.00 0.89 0.84 0.95 0.86 1.00 0.91 0.81 0.77 0.61
H3
GAPDH
HN4
GLI1
1.00 0.81 0.69 0.88 0.75 1.00 0.80 0.76 0.14 0.10
H3
GAPDH
CAL27
(The third time)
NC
si-CSNK1D#1
si-CSNK1D#2
Importazole
GLI1
1.00 0.76 0.71 0.78 0.73 1.00 0.88 0.68 0.62 0.17
H3
GAPDH
HN4
GLI1
1.00 0.89 0.78 0.90 0.85 1.00 0.98 0.93 0.78 0.68
H3
GAPDH
CAL27

Fig.5B

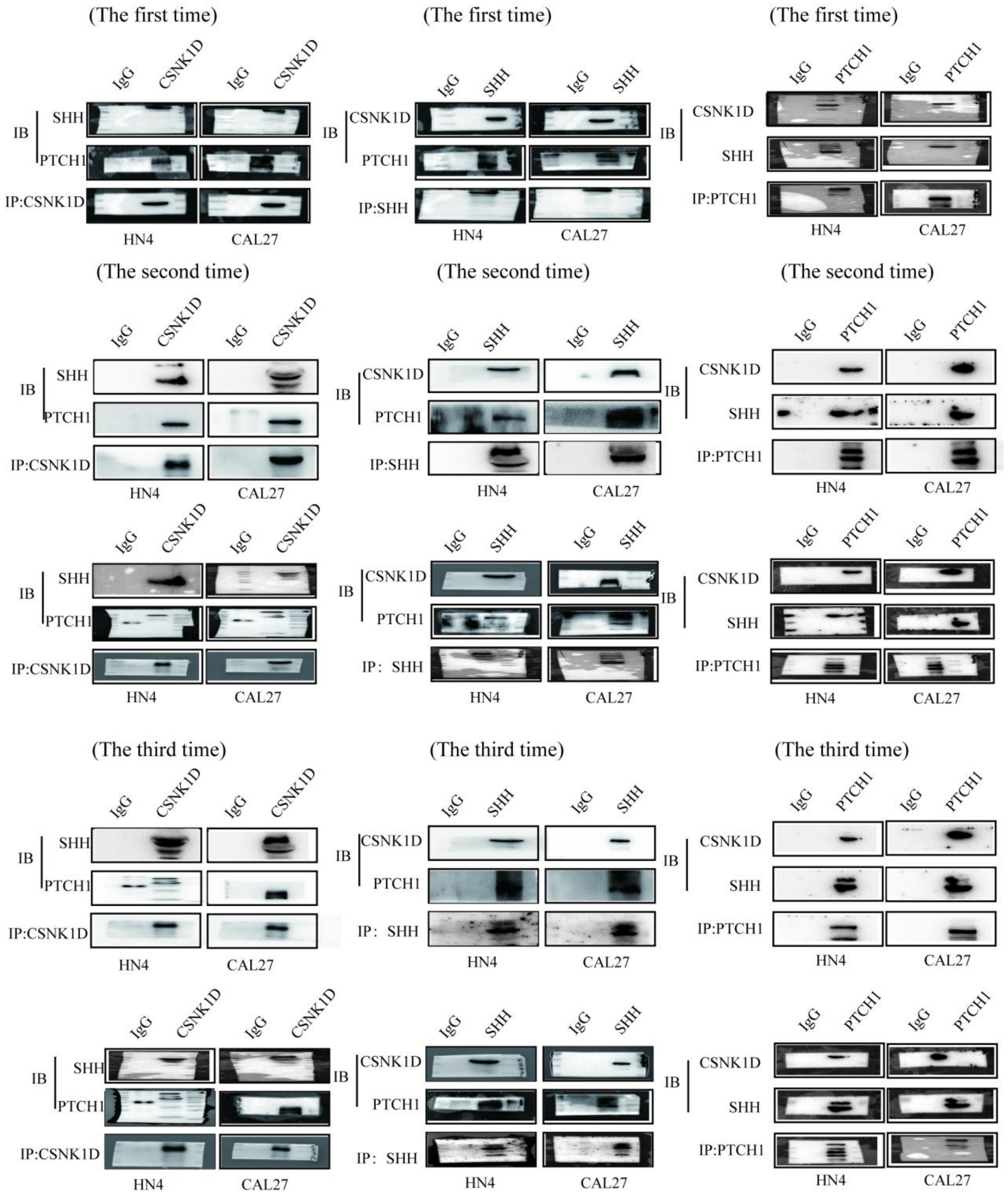
(The first time)
IgG
CSNK1D
IgG
CSNK1D
IB
SHH
PTCH1
IP:CSNK1D
HN4
CAL27
(The first time)
IgG
SHH
IgG
SHH
IB
CSNK1D
PTCH1
IP:SHH
HN4
CAL27
(The first time)
IgG
PTCH1
IgG
PTCH1
IB
CSNK1D
SHH
IP:PTCH1
HN4
CAL27
(The second time)
IgG
CSNK1D
IgG
CSNK1D
IB
SHH
PTCH1
IP:CSNK1D
HN4
CAL27
(The second time)
IgG
SHH
IgG
SHH
IB
CSNK1D
PTCH1
IP:SHH
HN4
CAL27
(The second time)
IgG
PTCH1
IgG
PTCH1
IB
CSNK1D
SHH
IP:PTCH1
HN4
CAL27
IgG
CSNK1D
IgG
CSNK1D
IB
SHH
PTCH1
IP:CSNK1D
HN4
CAL27
IgG
SHH
IgG
SHH
IB
CSNK1D
PTCH1
IP： SHH
HN4
CAL27
IgG
PTCH1
IgG
PTCH1
IB
CSNK1D
SHH
IP:PTCH1
HN4
CAL27
(The third time)
IgG
CSNK1D
IgG
CSNK1D
IB
SHH
PTCH1
IP:CSNK1D
HN4
CAL27
(The third time)
IgG
SHH
IgG
SHH
IB
CSNK1D
PTCH1
IP： SHH
HN4
CAL27
(The third time)
IgG
PTCH1
IgG
PTCH1
IB
CSNK1D
SHH
IP:PTCH1
HN4
CAL27
IgG
CSNK1D
IgG
CSNK1D
IB
SHH
PTCH1
IP:CSNK1D
HN4
CAL27
IgG
SHH
IgG
SHH
IB
CSNK1D
PTCH1
IP： SHH
HN4
CAL27
IgG
PTCH1
IgG
PTCH1
IB
CSNK1D
SHH
IP:PTCH1
HN4
CAL27

Fig.5C

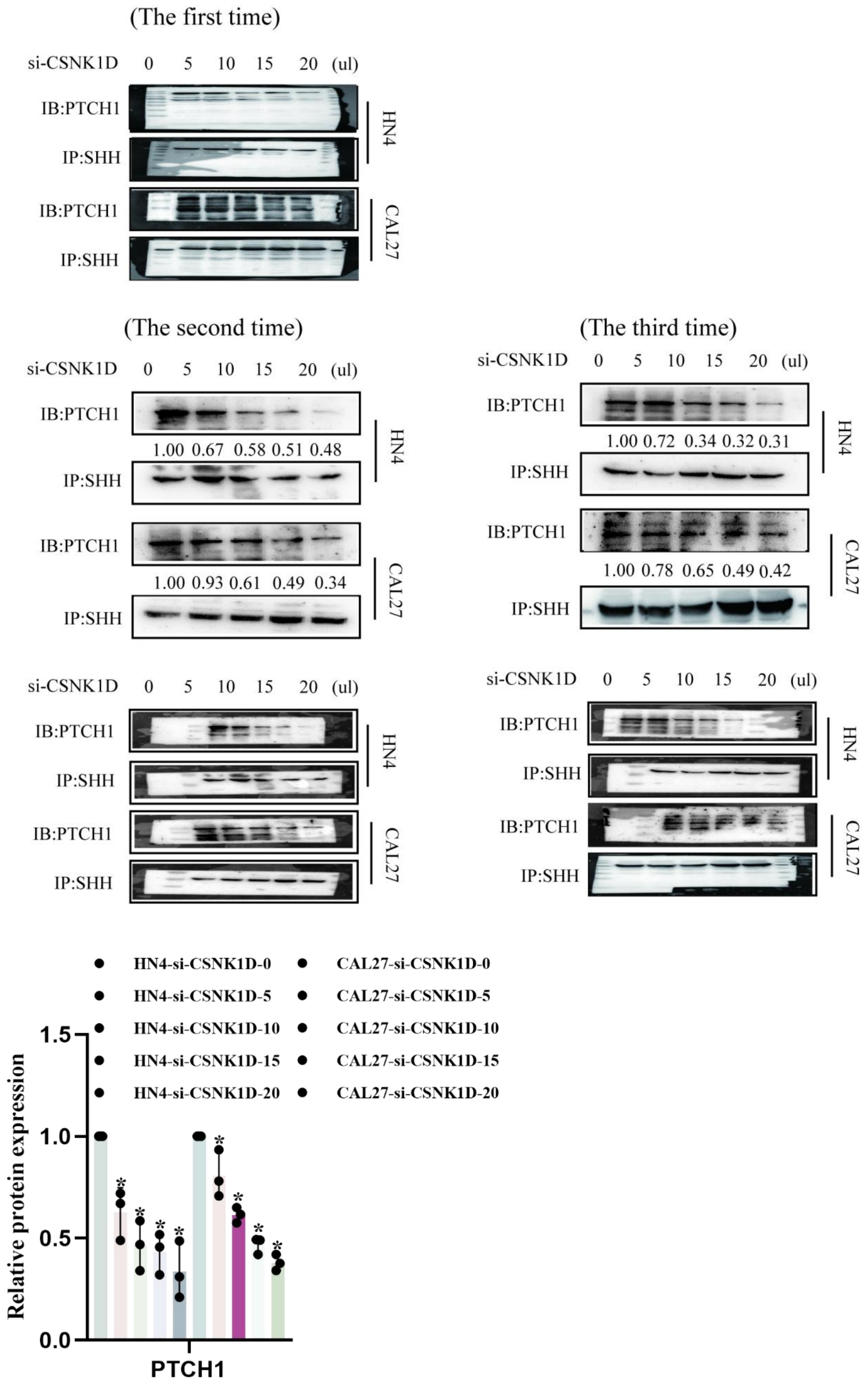
(The first time)
si-CSNK1D 0 5 10 15 20 (ul)
IB:PTCH1
IP:SHH
HN4
IB:PTCH1
IP:SHH
CAL27
(The second time)
si-CSNK1D 0 5 10 15 20 (ul)
IB:PTCH1
1.00 0.67 0.58 0.51 0.48
IP:SHH
HN4
IB:PTCH1
1.00 0.93 0.61 0.49 0.34
IP:SHH
CAL27
(The third time)
si-CSNK1D 0 5 10 15 20 (ul)
IB:PTCH1
1.00 0.72 0.34 0.32 0.31
IP:SHH
HN4
IB:PTCH1
1.00 0.78 0.65 0.49 0.42
IP:SHH
CAL27
si-CSNK1D 0 5 10 15 20 (ul)
IB:PTCH1
IP:SHH
HN4
IB:PTCH1
IP:SHH
CAL27
si-CSNK1D 0 5 10 15 20 (ul)
IB:PTCH1
IP:SHH
HN4
IB:PTCH1
IP:SHH
CAL27
HN4-si-CSNK1D-0
HN4-si-CSNK1D-5
HN4-si-CSNK1D-10
HN4-si-CSNK1D-15
HN4-si-CSNK1D-20
CAL27-si-CSNK1D-0
CAL27-si-CSNK1D-5
CAL27-si-CSNK1D-10
CAL27-si-CSNK1D-15
CAL27-si-CSNK1D-20
Relative protein expression
1.5
1.0
0.5
0.0
PTCH1

Fig.5E

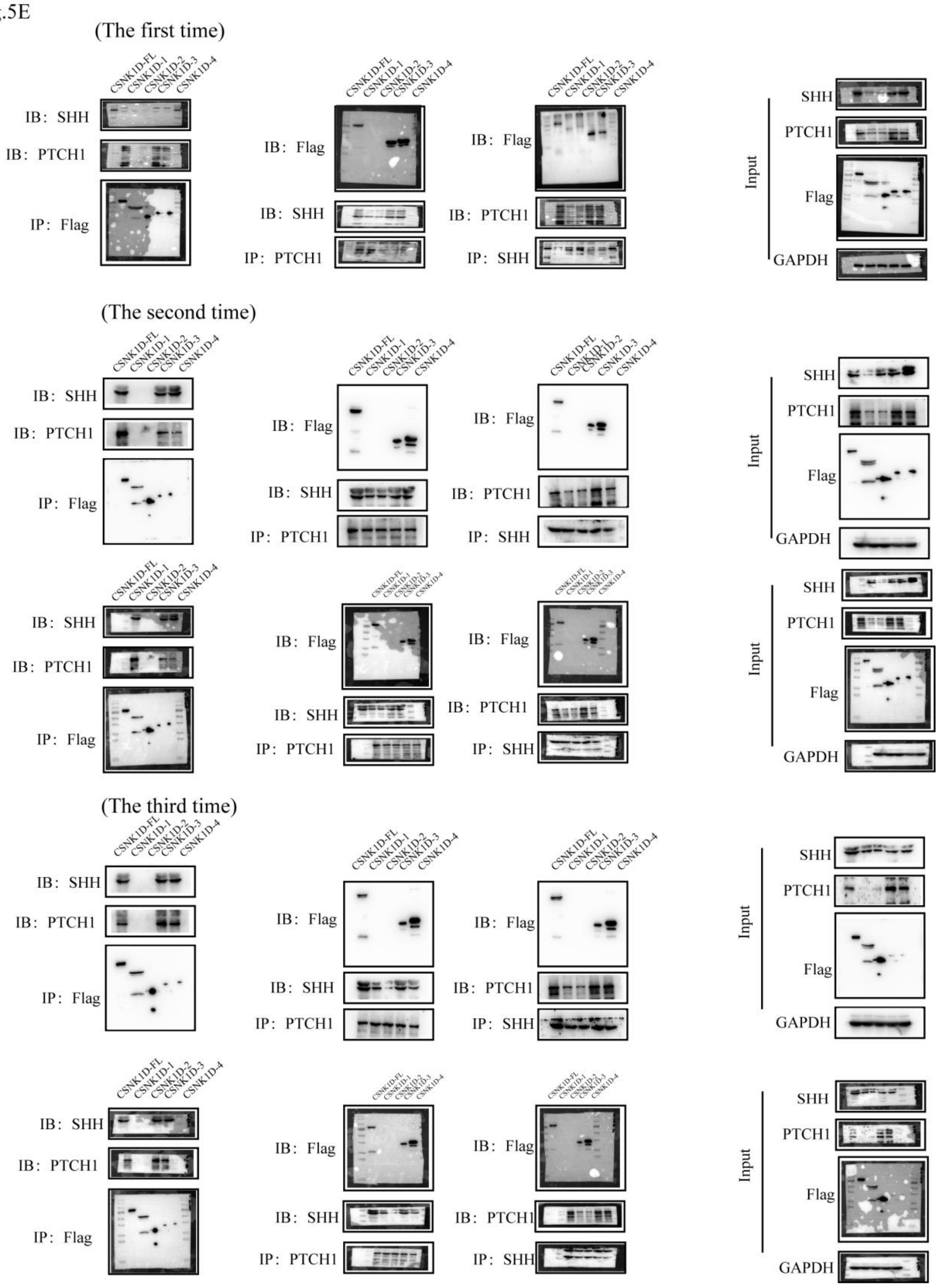
(The first time)
CSNK1D-FL
CSNK1D-1
CSNK1D-2
CSNK1D-3
CSNK1D-4
IB：SHH
IB：PTCH1
IP：Flag
IB：Flag
IB：SHH
IP：PTCH1
IB：Flag
IB：PTCH1
IP：SHH
Input
SHH
PTCH1
Flag
GAPDH
(The second time)
CSNK1D-FL
CSNK1D-1
CSNK1D-2
CSNK1D-3
CSNK1D-4
IB：SHH
IB：PTCH1
IP：Flag
IB：Flag
IB：SHH
IP：PTCH1
IB：Flag
IB：PTCH1
IP：SHH
Input
SHH
PTCH1
Flag
GAPDH
(The third time)
CSNK1D-FL
CSNK1D-1
CSNK1D-2
CSNK1D-3
CSNK1D-4
IB：SHH
IB：PTCH1
IP：Flag
IB：Flag
IB：SHH
IP：PTCH1
IB：Flag
IB：PTCH1
IP：SHH
Input
SHH
PTCH1
Flag
GAPDH

Fig.5G

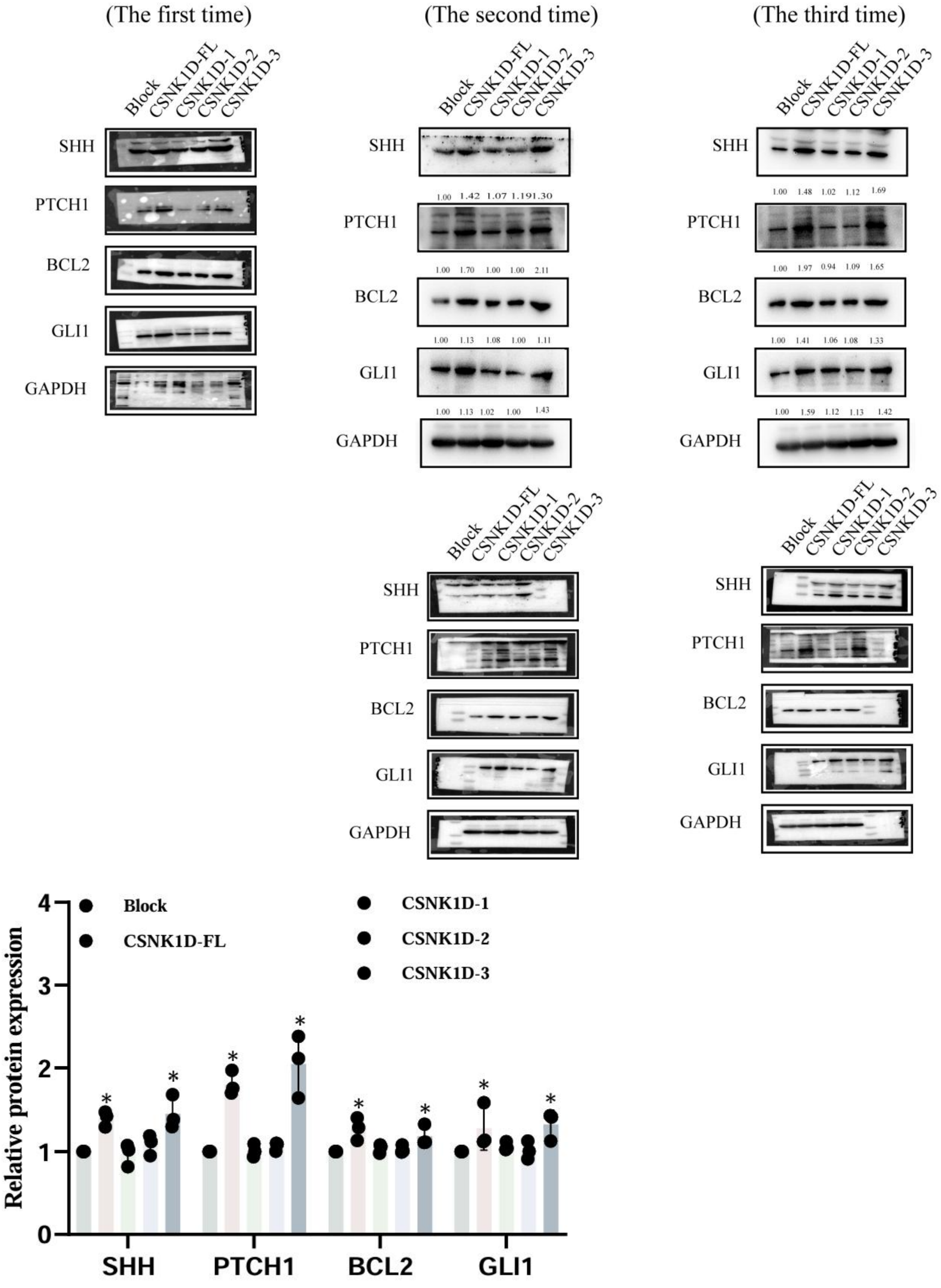

(The first time)
(The second time)
(The third time)
Block
CSNK1D-FL
CSNK1D-1
CSNK1D-2
CSNK1D-3
SHH
PTCH1
BCL2
GLI1
GAPDH
1.00 1.42 1.07 1.19 1.30
1.00 1.70 1.00 1.00 2.11
1.00 1.13 1.08 1.00 1.11
1.00 1.13 1.02 1.00 1.43
1.00 1.48 1.02 1.12 1.69
1.00 1.97 0.94 1.09 1.65
1.00 1.41 1.06 1.08 1.33
1.00 1.59 1.12 1.13 1.42
Relative protein expression
0
1
2
3
4
*

Fig.5H

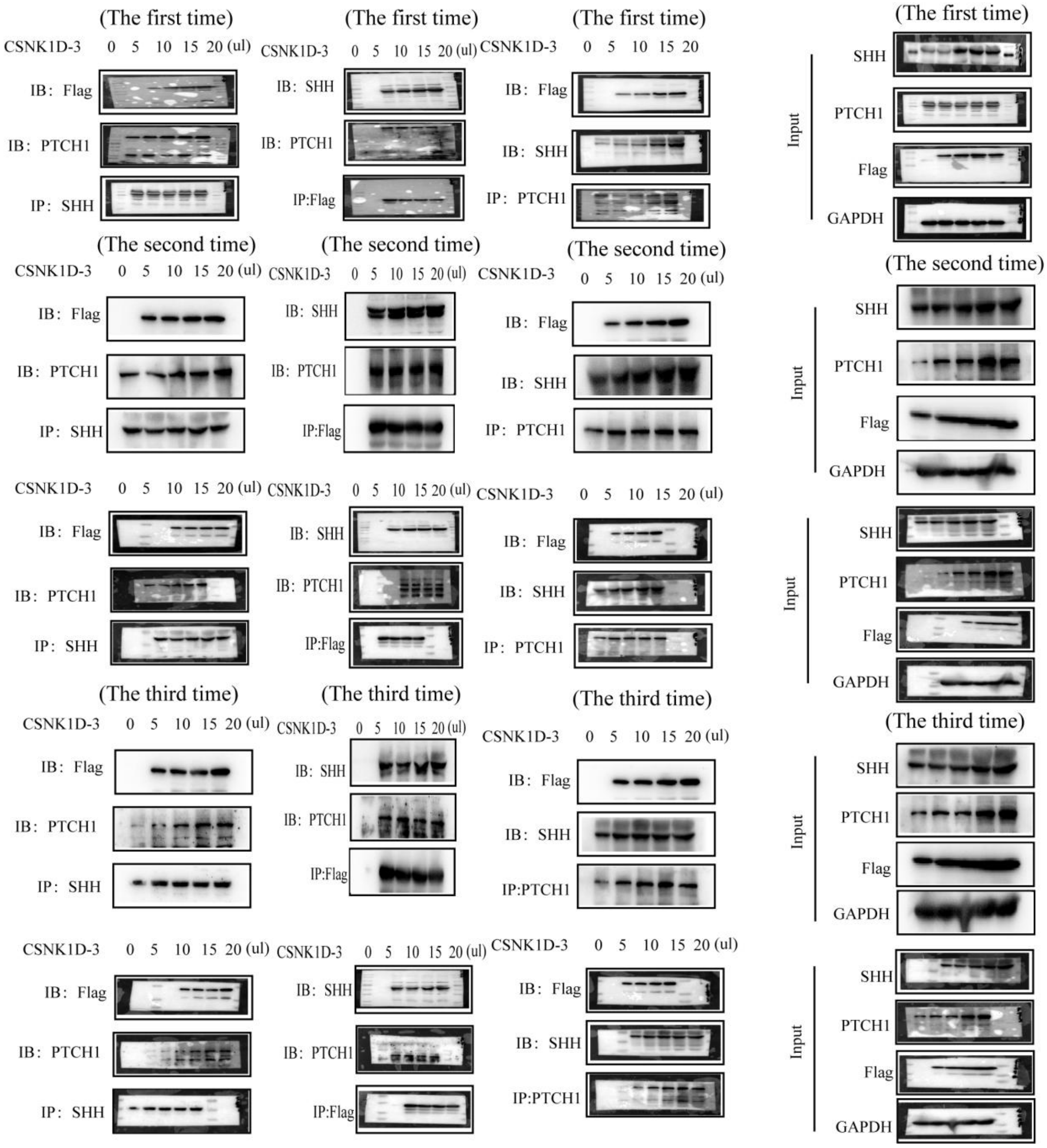
(The first time)
(The second time)
(The third time)
CSNK1D-3
0 5 10 15 20 (ul)
IB: Flag
IB: PTCH1
IP: SHH
IB: SHH
IP:Flag
IP: PTCH1
Input
SHH
PTCH1
Flag
GAPDH

Fig.6E

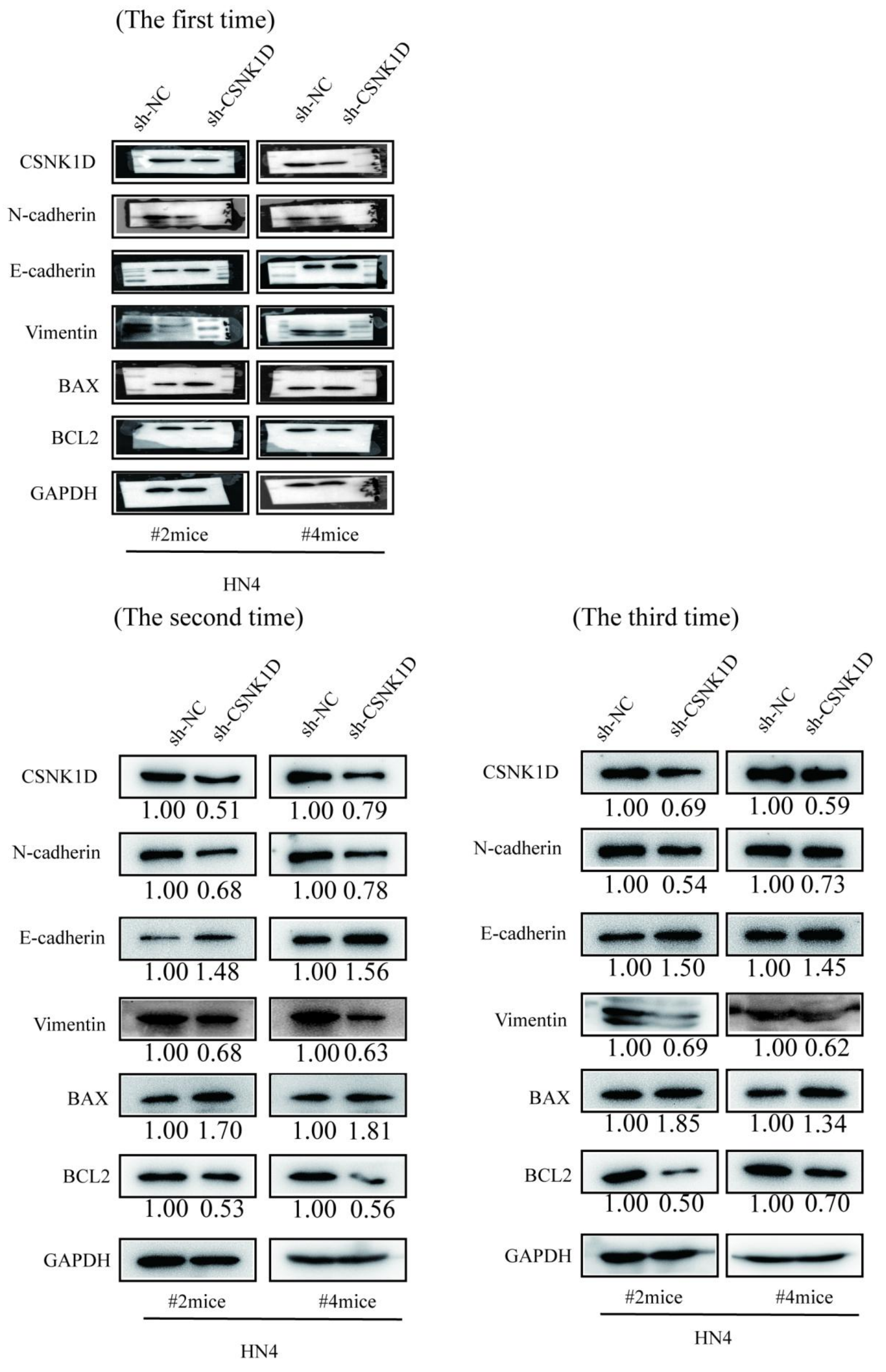

(The first time)
sh-NC
sh-CSNK1D
sh-NC
sh-CSNK1D
CSNK1D
N-cadherin
E-cadherin
Vimentin
BAX
BCL2
GAPDH
#2mice
#4mice
HN4
(The second time)
sh-NC
sh-CSNK1D
sh-NC
sh-CSNK1D
CSNK1D
1.00 0.51
1.00 0.79
N-cadherin
1.00 0.68
1.00 0.78
E-cadherin
1.00 1.48
1.00 1.56
Vimentin
1.00 0.68
1.00 0.63
BAX
1.00 1.70
1.00 1.81
BCL2
1.00 0.53
1.00 0.56
GAPDH
#2mice
#4mice
HN4
(The third time)
sh-NC
sh-CSNK1D
sh-NC
sh-CSNK1D
CSNK1D
1.00 0.69
1.00 0.59
N-cadherin
1.00 0.54
1.00 0.73
E-cadherin
1.00 1.50
1.00 1.45
Vimentin
1.00 0.69
1.00 0.62
BAX
1.00 1.85
1.00 1.34
BCL2
1.00 0.50
1.00 0.70
GAPDH
#2mice
#4mice
HN4

Fig.6E

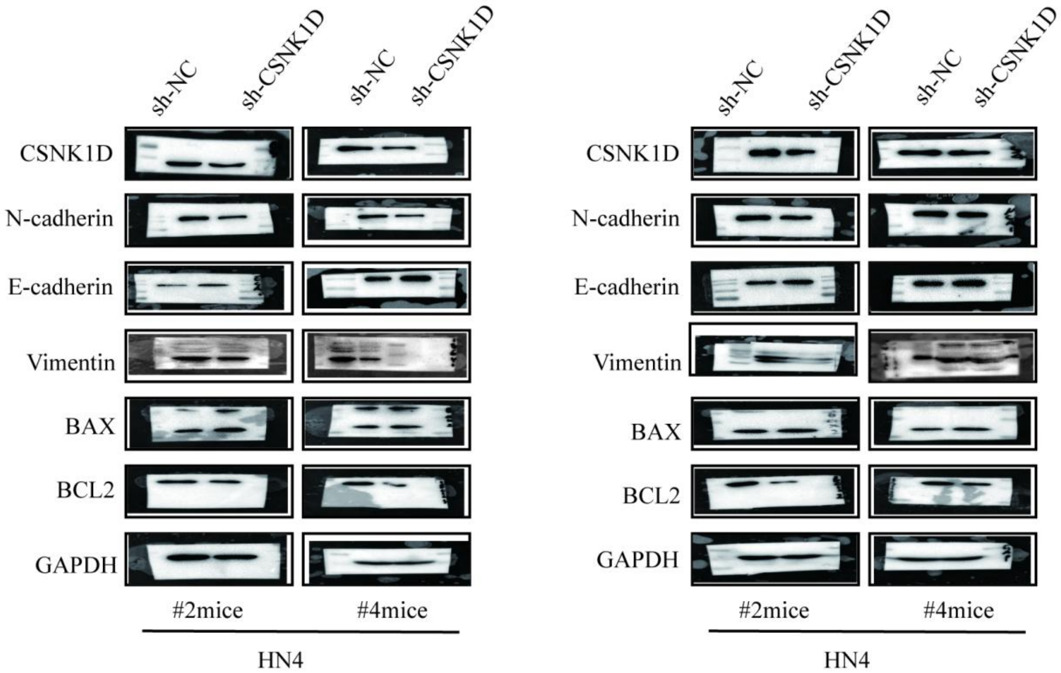

Fig.6E

(The first time)

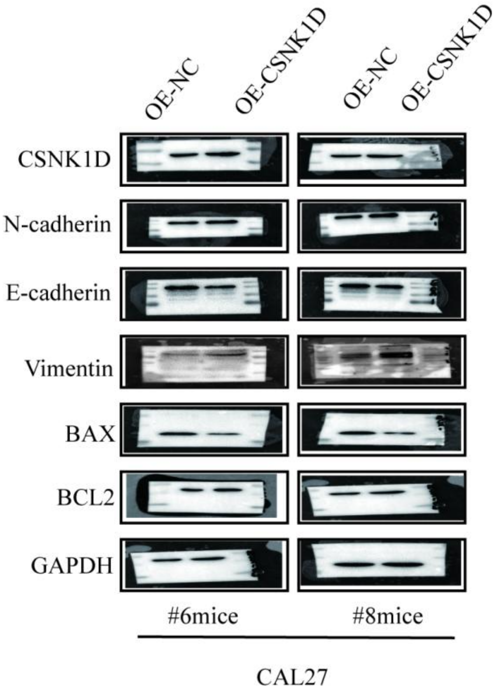

(The second time)

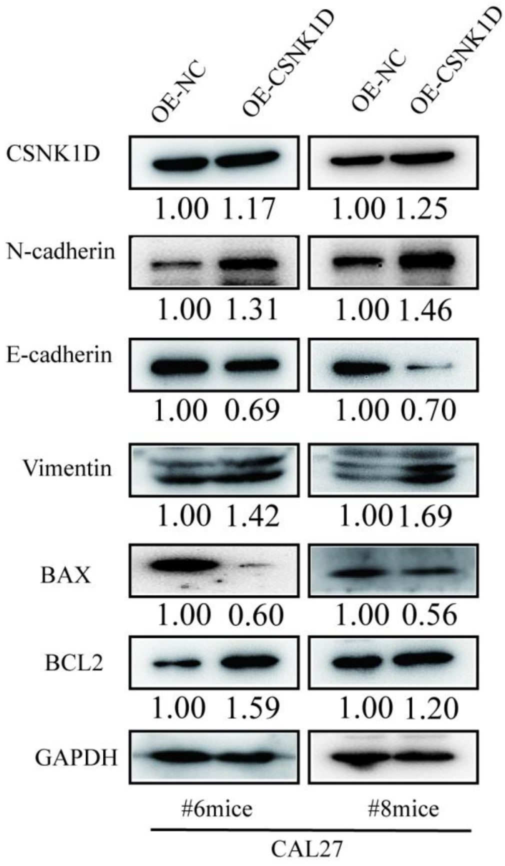

(The third time)

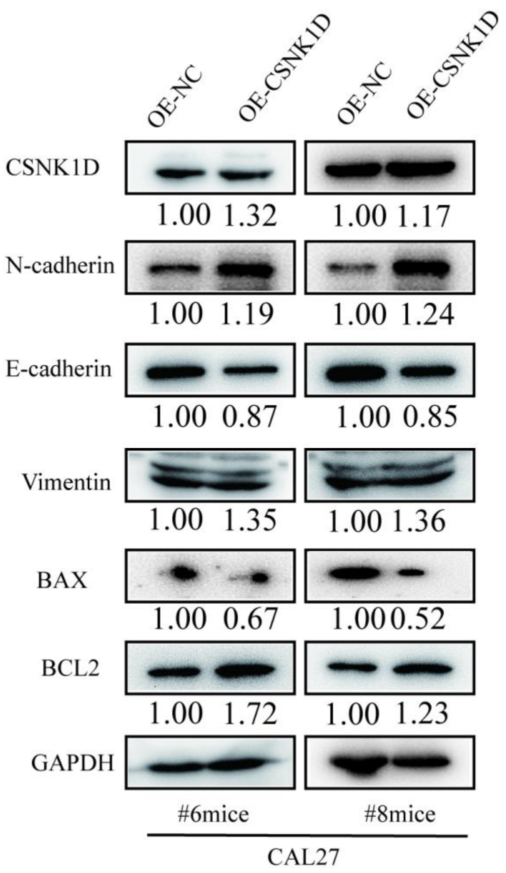

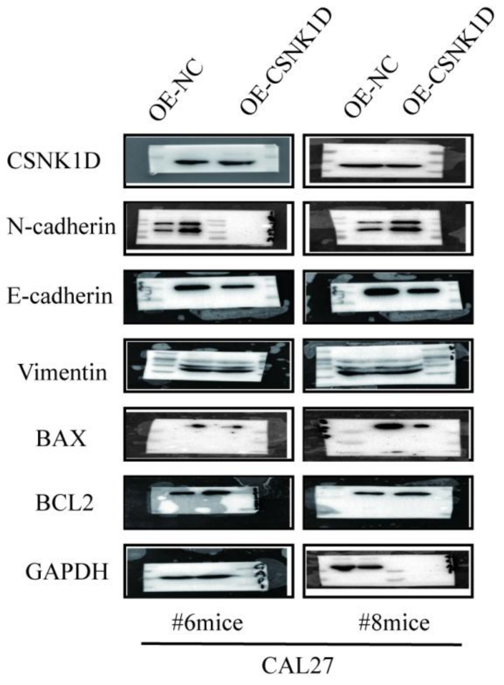

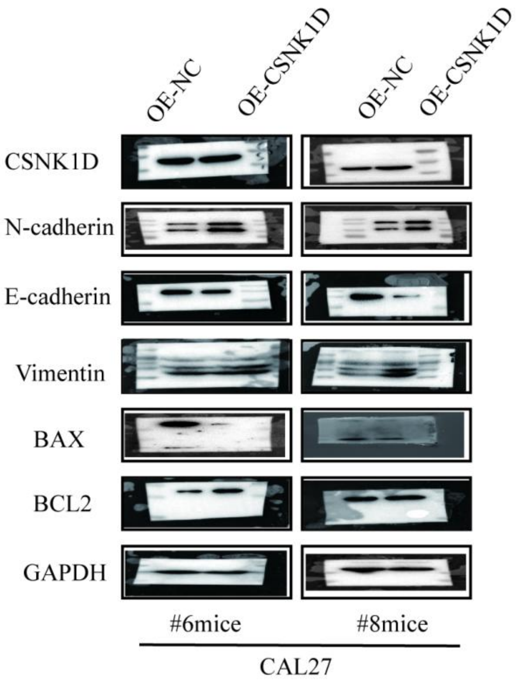

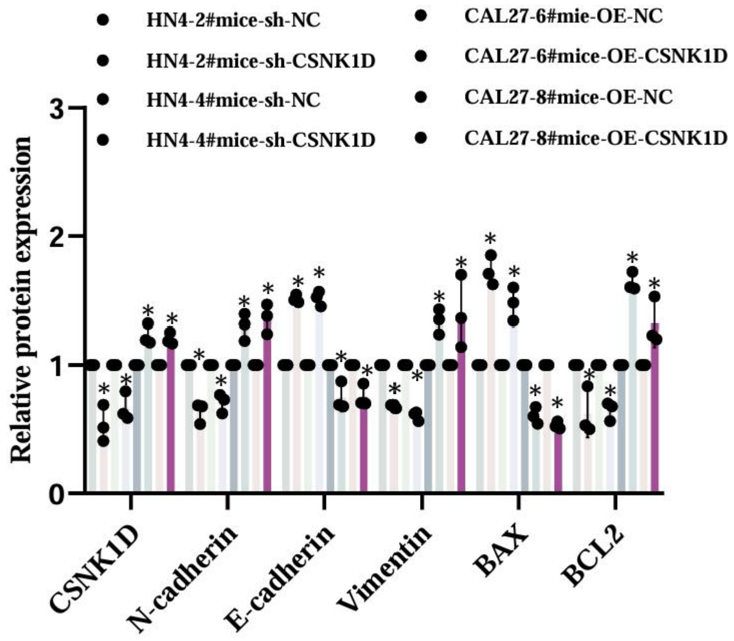

Fig.6I

(The first time)

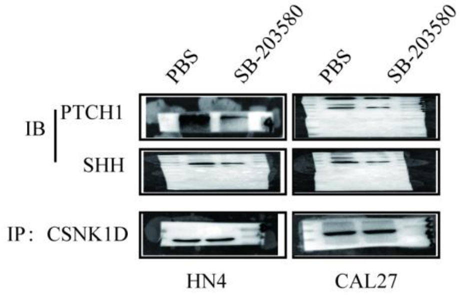

(The second time)

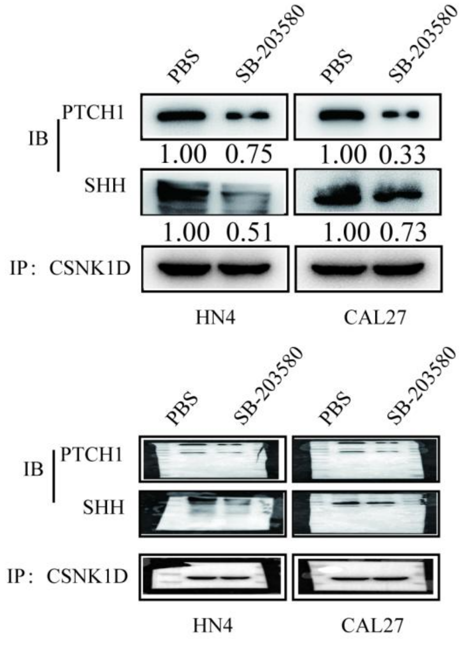

(The third time)

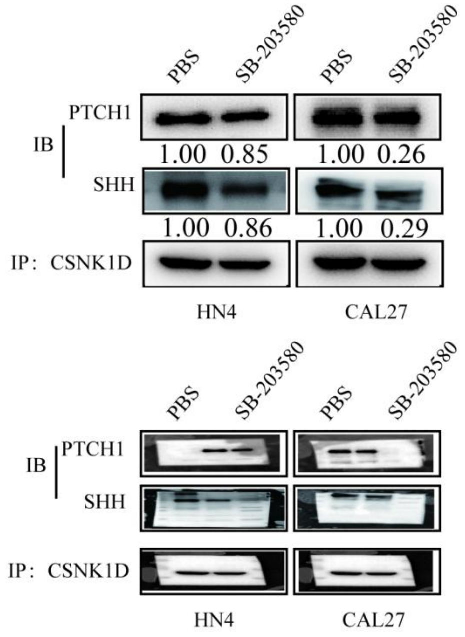

Figure S1. B

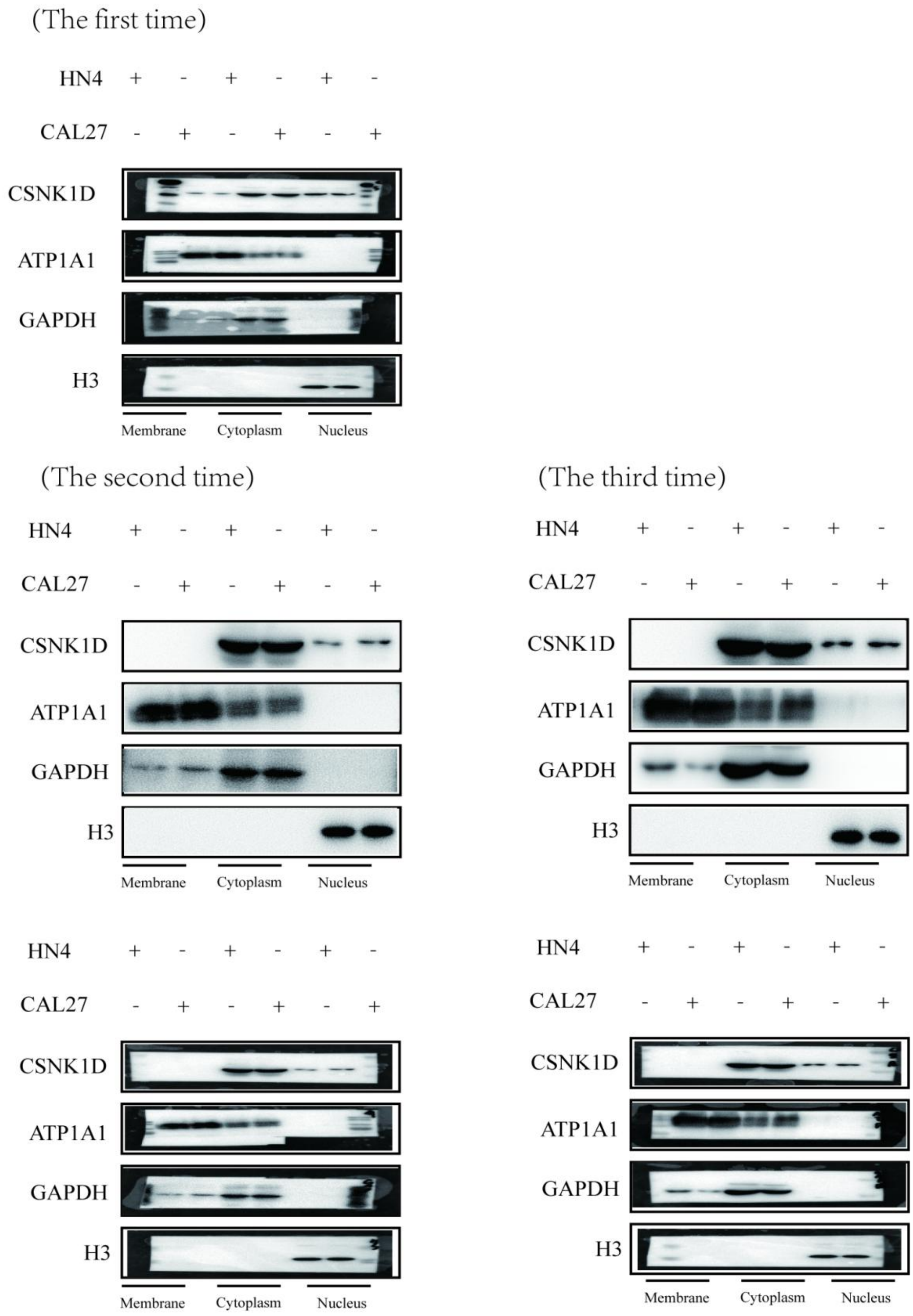
(The first time)
HN4 + - + - + -
CAL27 - + - + - +
CSNK1D
ATP1A1
GAPDH
H3
Membrane
Cytoplasm
Nucleus
(The second time)
HN4 + - + - + -
CAL27 - + - + - +
CSNK1D
ATP1A1
GAPDH
H3
Membrane
Cytoplasm
Nucleus
(The third time)
HN4 + - + - + -
CAL27 - + - + - +
CSNK1D
ATP1A1
GAPDH
H3
Membrane
Cytoplasm
Nucleus
HN4 + - + - + -
CAL27 - + - + - +
CSNK1D
ATP1A1
GAPDH
H3
Membrane
Cytoplasm
Nucleus
HN4 + - + - + -
CAL27 - + - + - +
CSNK1D
ATP1A1
GAPDH
H3
Membrane
Cytoplasm
Nucleus

Figure S2. B

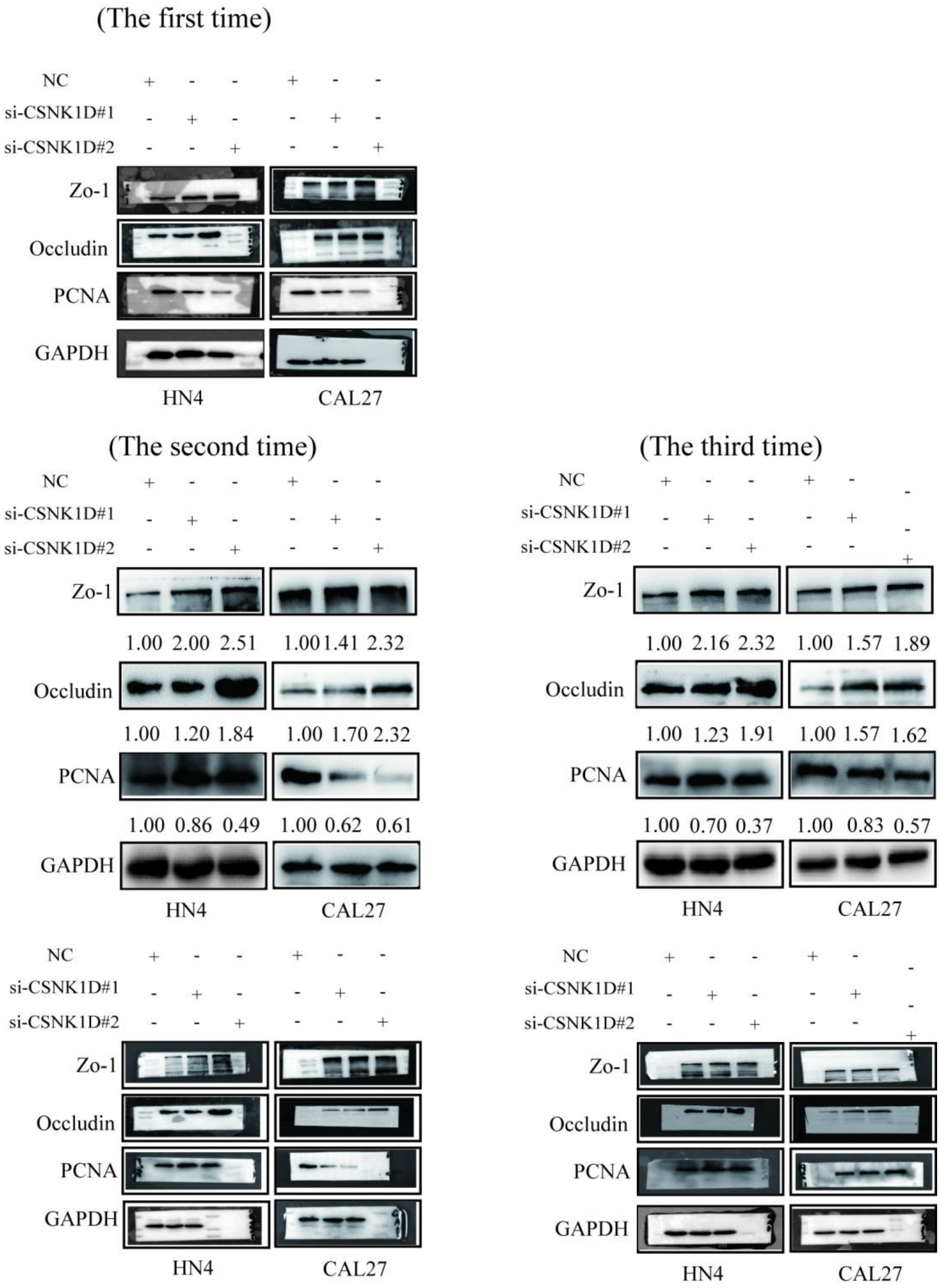
(The first time)
NC
si-CSNK1D#1
si-CSNK1D#2
Zo-1
Occludin
PCNA
GAPDH
HN4
CAL27
(The second time)
1.00 2.00 2.51 1.00 1.41 2.32
1.00 1.20 1.84 1.00 1.70 2.32
1.00 0.86 0.49 1.00 0.62 0.61
(The third time)
1.00 2.16 2.32 1.00 1.57 1.89
1.00 1.23 1.91 1.00 1.57 1.62
1.00 0.70 0.37 1.00 0.83 0.57

(The first time)

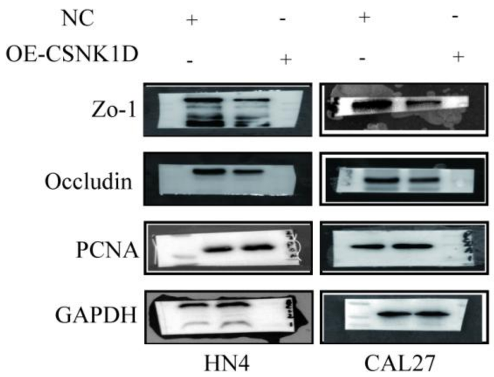

(The second time)

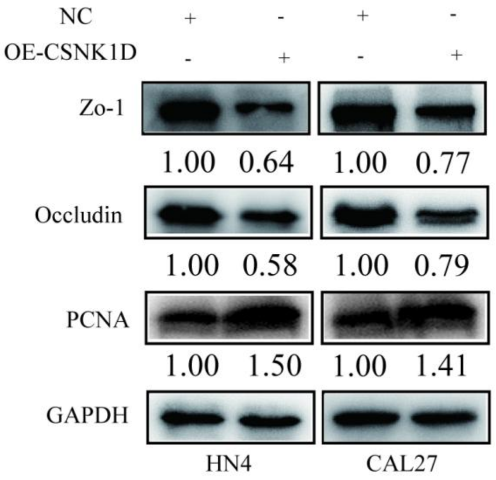

(The third time)

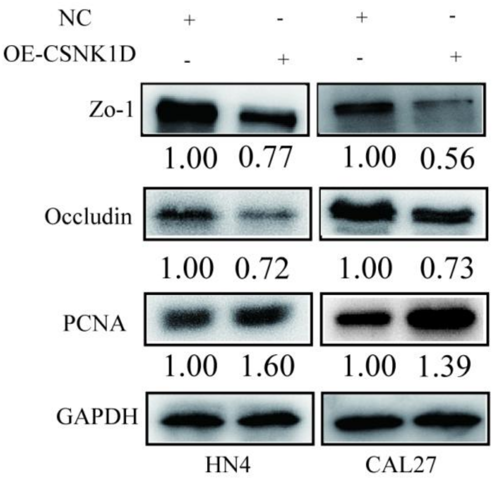

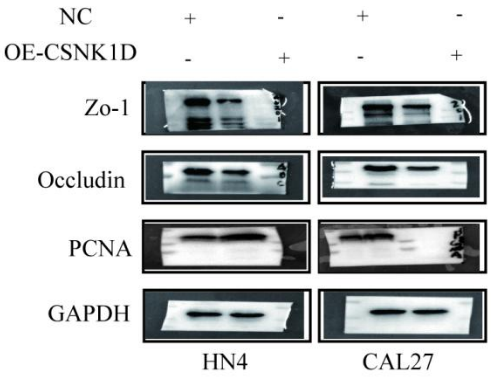

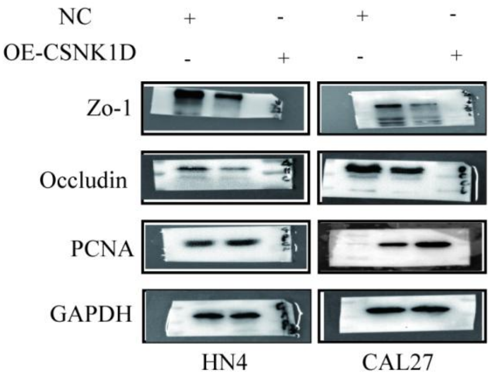

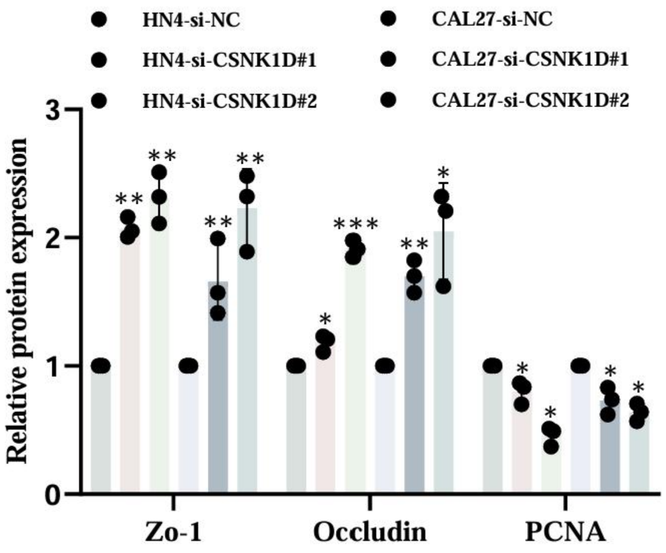
HN4-si-NC
HN4-si-CSNK1D#1
HN4-si-CSNK1D#2
CAL27-si-NC
CAL27-si-CSNK1D#1
CAL27-si-CSNK1D#2
Relative protein expression
0
1
2
3
Zo-1
Occludin
PCNA

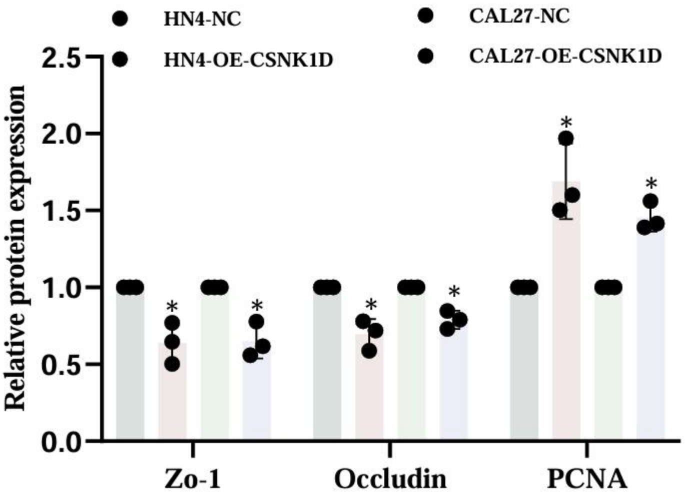
HN4-NC
HN4-OE-CSNK1D
CAL27-NC
CAL27-OE-CSNK1D
Relative protein expression
0.0
0.5
1.0
1.5
2.0
2.5
Zo-1
Occludin
PCNA

Figure S3. C

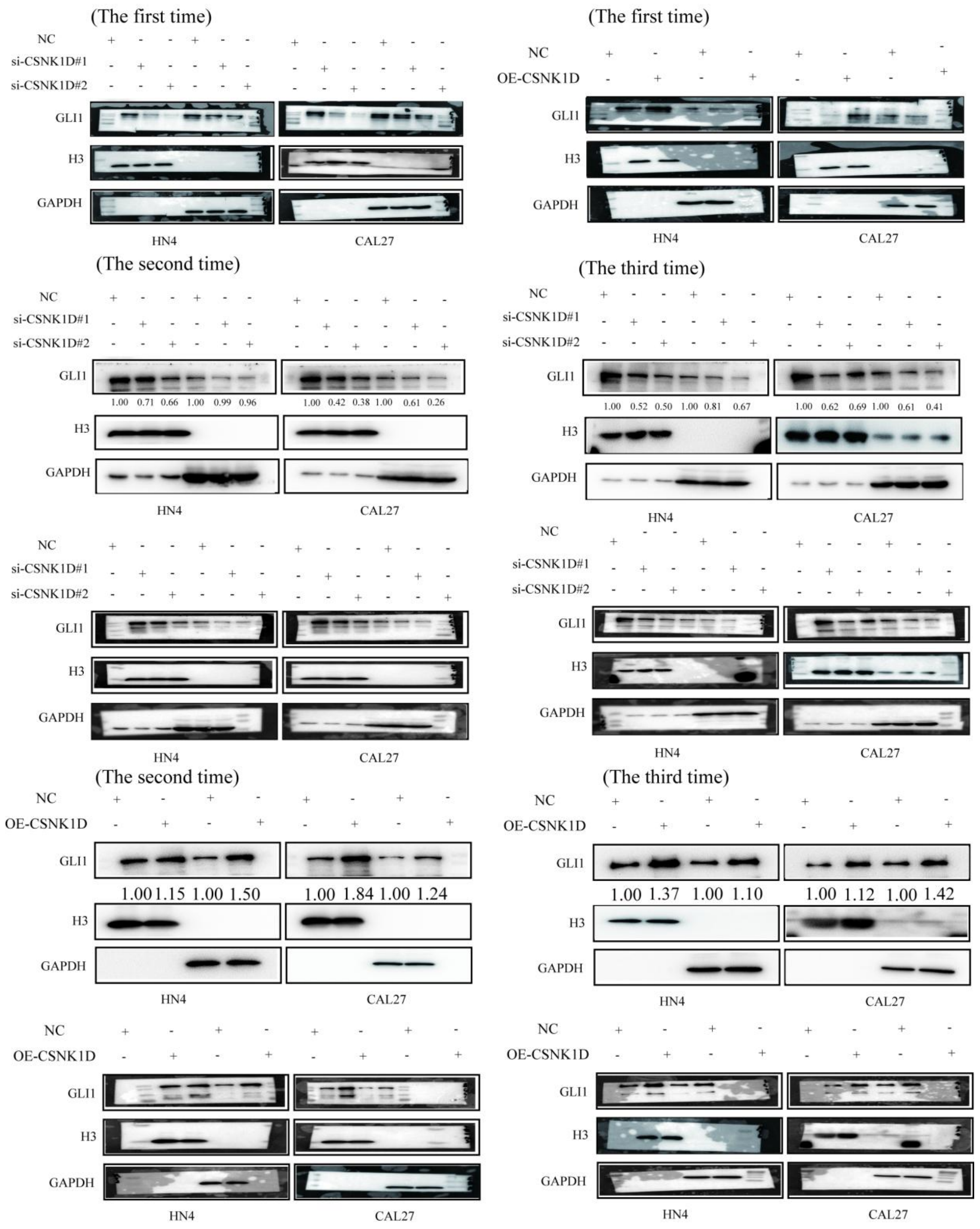
(The first time)
NC
si-CSNK1D#1
si-CSNK1D#2
GLI1
H3
GAPDH
HN4
CAL27
(The first time)
NC
OE-CSNK1D
GLI1
H3
GAPDH
HN4
CAL27
(The second time)
NC
si-CSNK1D#1
si-CSNK1D#2
GLI1
1.00 0.71 0.66 1.00 0.99 0.96
1.00 0.42 0.38 1.00 0.61 0.26
H3
GAPDH
HN4
CAL27
(The third time)
NC
si-CSNK1D#1
si-CSNK1D#2
GLI1
1.00 0.52 0.50 1.00 0.81 0.67
1.00 0.62 0.69 1.00 0.61 0.41
H3
GAPDH
HN4
CAL27
NC
si-CSNK1D#1
si-CSNK1D#2
GLI1
H3
GAPDH
HN4
CAL27
NC
si-CSNK1D#1
si-CSNK1D#2
GLI1
H3
GAPDH
HN4
CAL27
(The second time)
NC
OE-CSNK1D
GLI1
1.00 1.15 1.00 1.50
1.00 1.84 1.00 1.24
H3
GAPDH
HN4
CAL27
(The third time)
NC
OE-CSNK1D
GLI1
1.00 1.37 1.00 1.10
1.00 1.12 1.00 1.42
H3
GAPDH
HN4
CAL27
NC
OE-CSNK1D
GLI1
H3
GAPDH
HN4
CAL27
NC
OE-CSNK1D
GLI1
H3
GAPDH
HN4
CAL27

Figure S3. D

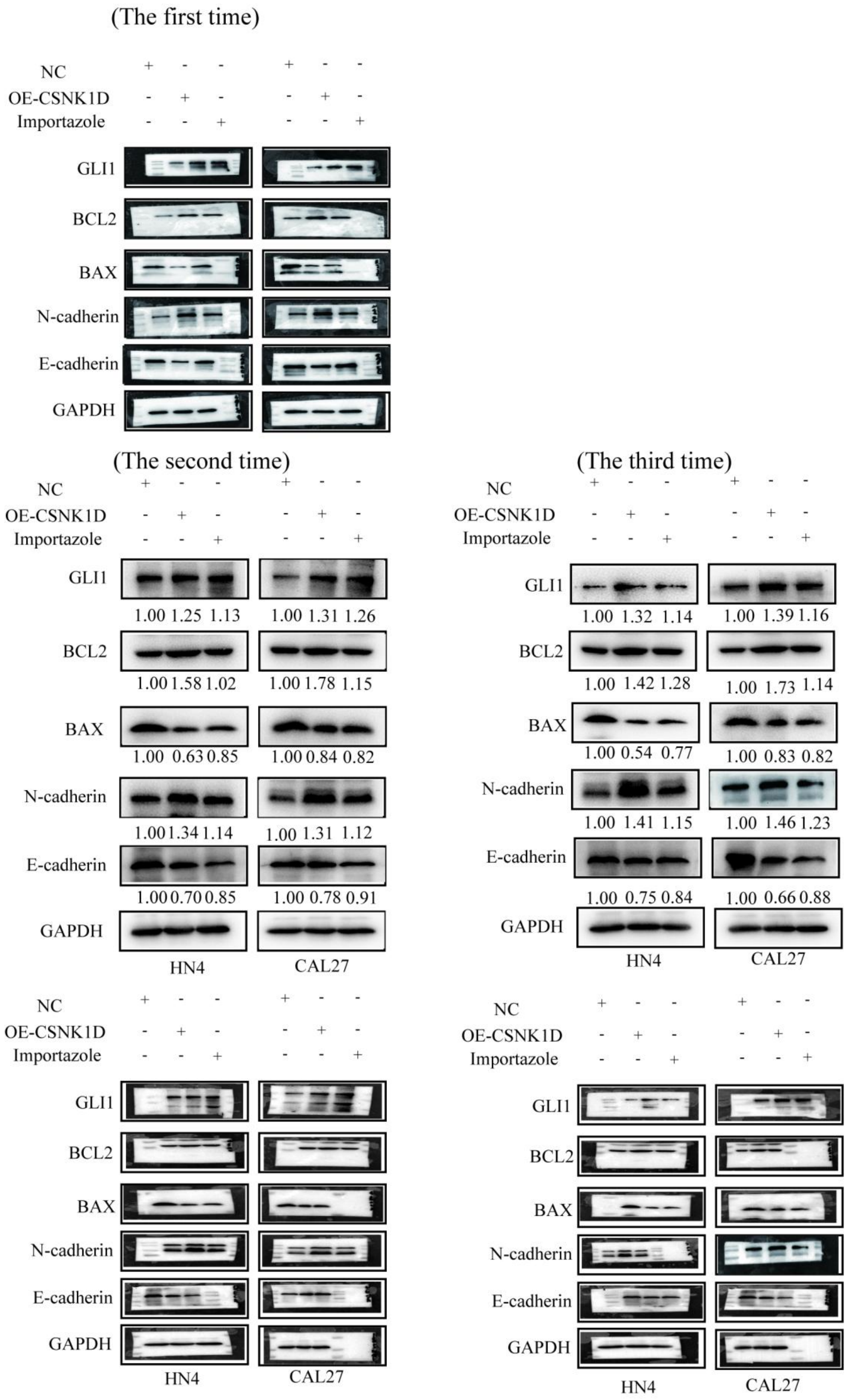
(The first time)
NC + - - + - -
OE-CSNK1D - + - - + -
Importazole - - + - - +
GLI1
BCL2
BAX
N-cadherin
E-cadherin
GAPDH
(The second time)
NC + - - + - -
OE-CSNK1D - + - - + -
Importazole - - + - - +
GLI1
1.00 1.25 1.13 1.00 1.31 1.26
BCL2
1.00 1.58 1.02 1.00 1.78 1.15
BAX
1.00 0.63 0.85 1.00 0.84 0.82
N-cadherin
1.00 1.34 1.14 1.00 1.31 1.12
E-cadherin
1.00 0.70 0.85 1.00 0.78 0.91
GAPDH
HN4 CAL27
(The third time)
NC + - - + - -
OE-CSNK1D - + - - + -
Importazole - - + - - +
GLI1
1.00 1.32 1.14 1.00 1.39 1.16
BCL2
1.00 1.42 1.28 1.00 1.73 1.14
BAX
1.00 0.54 0.77 1.00 0.83 0.82
N-cadherin
1.00 1.41 1.15 1.00 1.46 1.23
E-cadherin
1.00 0.75 0.84 1.00 0.66 0.88
GAPDH
HN4 CAL27
NC + - - + - -
OE-CSNK1D - + - - + -
Importazole - - + - - +
GLI1
BCL2
BAX
N-cadherin
E-cadherin
GAPDH
HN4 CAL27
NC + - - + - -
OE-CSNK1D - + - - + -
Importazole - - + - - +
GLI1
BCL2
BAX
N-cadherin
E-cadherin
GAPDH
HN4 CAL27

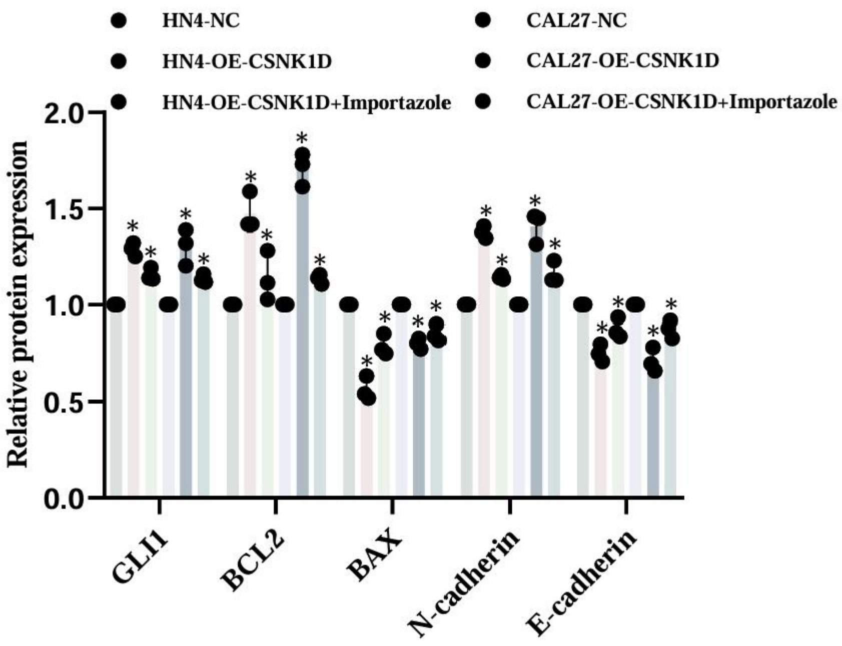

HN4-NC
HN4-OE-CSNK1D
HN4-OE-CSNK1D+Importazole
CAL27-NC
CAL27-OE-CSNK1D
CAL27-OE-CSNK1D+Importazole
Relative protein expression
2.0
1.5
1.0
0.5
0.0
GLI1
BCL2
BAX
N-cadherin
E-cadherin

Figure S3. D

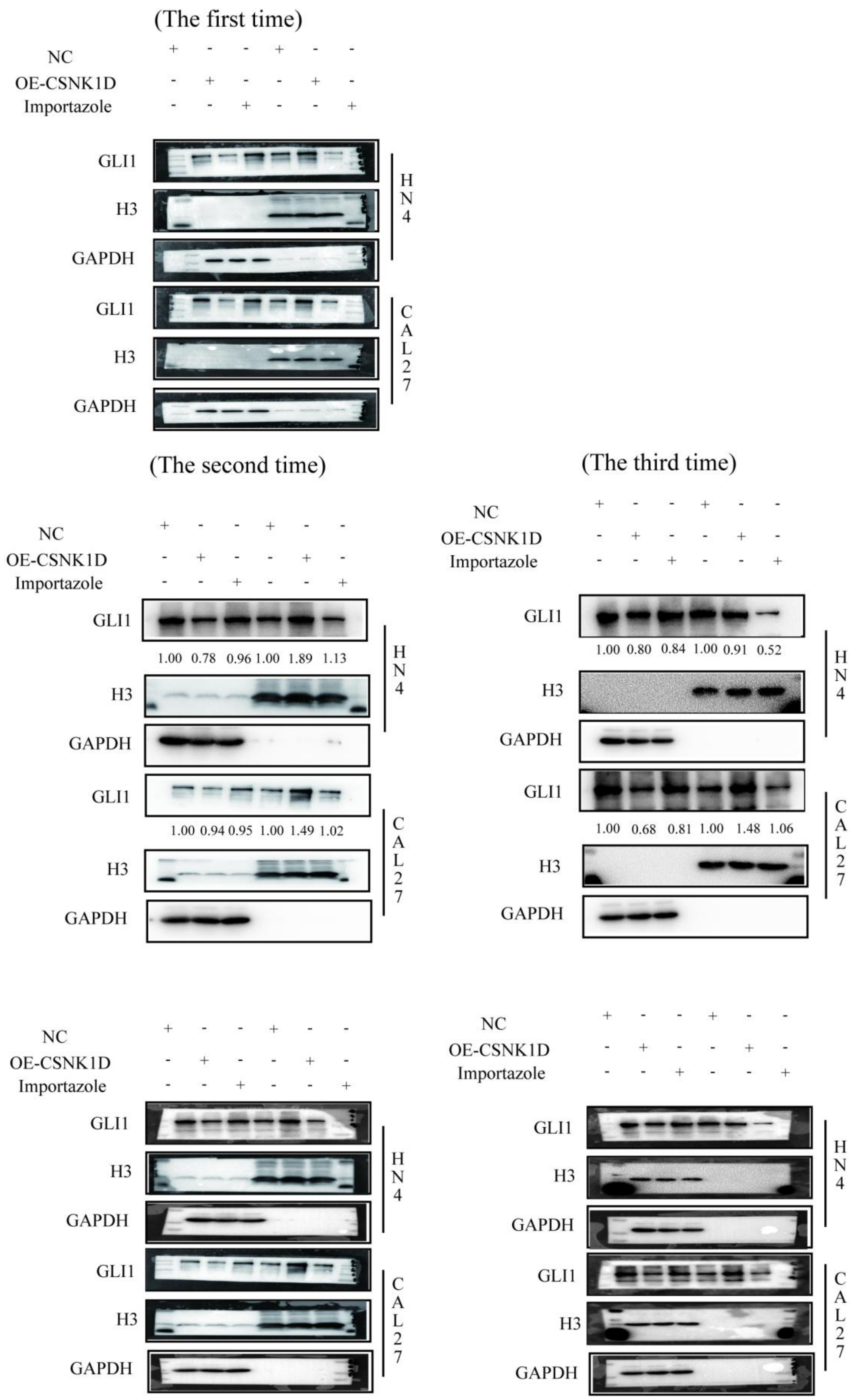
(The first time)
NC
OE-CSNK1D
Importazole
GLI1
H3
GAPDH
HN4
GLI1
H3
GAPDH
CAL27
(The second time)
NC
OE-CSNK1D
Importazole
GLI1
1.00 0.78 0.96 1.00 1.89 1.13
H3
GAPDH
HN4
GLI1
1.00 0.94 0.95 1.00 1.49 1.02
H3
GAPDH
CAL27
(The third time)
NC
OE-CSNK1D
Importazole
GLI1
1.00 0.80 0.84 1.00 0.91 0.52
H3
GAPDH
HN4
GLI1
1.00 0.68 0.81 1.00 1.48 1.06
H3
GAPDH
CAL27
NC
OE-CSNK1D
Importazole
GLI1
H3
GAPDH
HN4
GLI1
H3
GAPDH
CAL27
NC
OE-CSNK1D
Importazole
GLI1
H3
GAPDH
HN4
GLI1
H3
GAPDH
CAL27

Figure S4. L

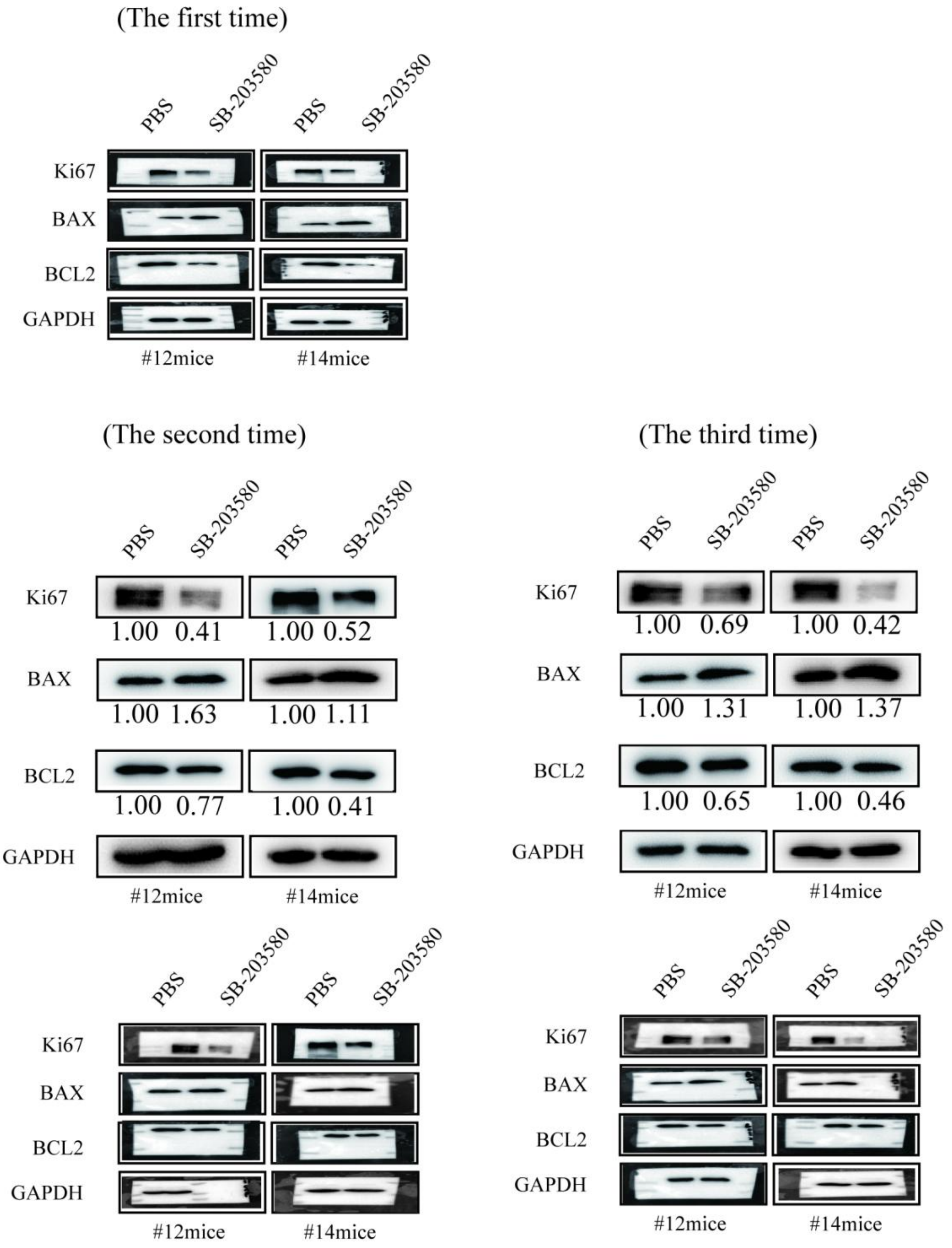
(The first time)
PBS
SB-203580
PBS
SB-203580
Ki67
BAX
BCL2
GAPDH
#12mice
#14mice
(The second time)
PBS
SB-203580
PBS
SB-203580
Ki67
1.00 0.41
1.00 0.52
BAX
1.00 1.63
1.00 1.11
BCL2
1.00 0.77
1.00 0.41
GAPDH
#12mice
#14mice
(The third time)
PBS
SB-203580
PBS
SB-203580
Ki67
1.00 0.69
1.00 0.42
BAX
1.00 1.31
1.00 1.37
BCL2
1.00 0.65
1.00 0.46
GAPDH
#12mice
#14mice
PBS
SB-203580
PBS
SB-203580
Ki67
BAX
BCL2
GAPDH
#12mice
#14mice
PBS
SB-203580
PBS
SB-203580
Ki67
BAX
BCL2
GAPDH
#12mice
#14mice

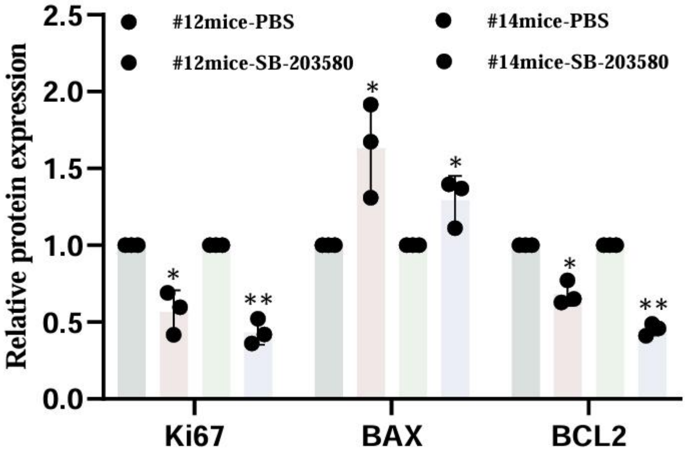

#12mice-PBS
#14mice-PBS
#12mice-SB-203580
#14mice-SB-203580
Relative protein expression
2.5
2.0
1.5
1.0
0.5
0.0
Ki67
BAX
BCL2

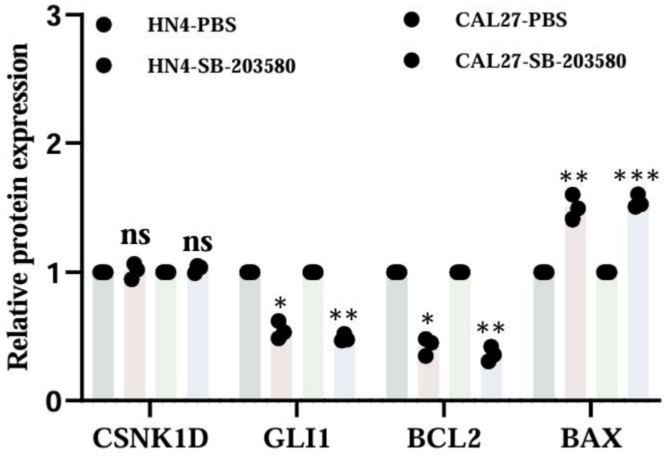

HN4-PBS
CAL27-PBS
HN4-SB-203580
CAL27-SB-203580
Relative protein expression
3
2
1
0
ns
CSNK1D
GLI1
BCL2
BAX

Figure S4. I

(The first time)

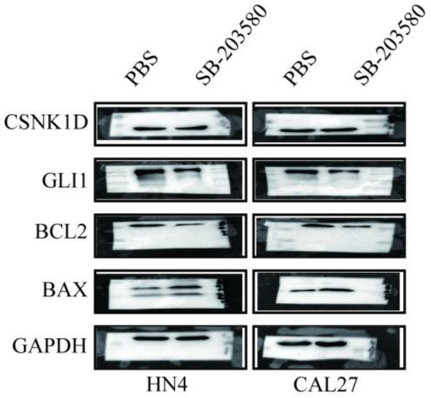

(The second time)

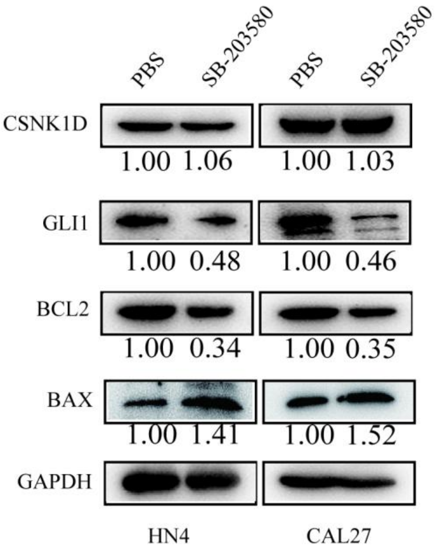

(The third time)

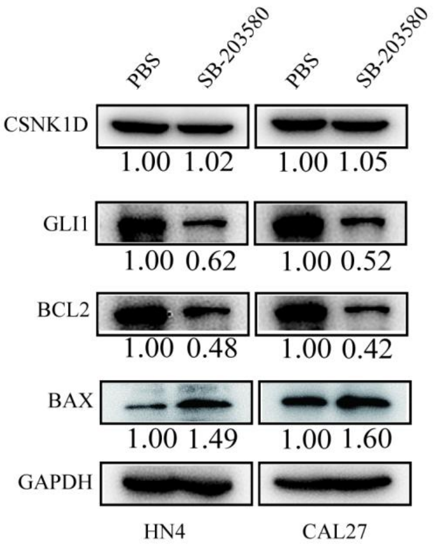

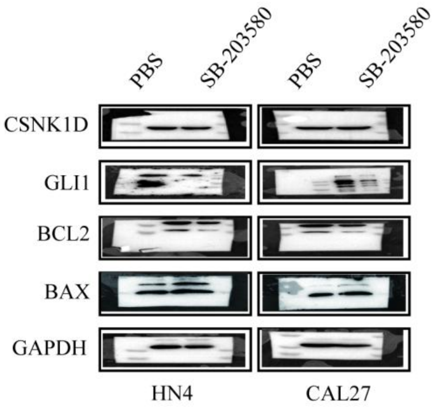

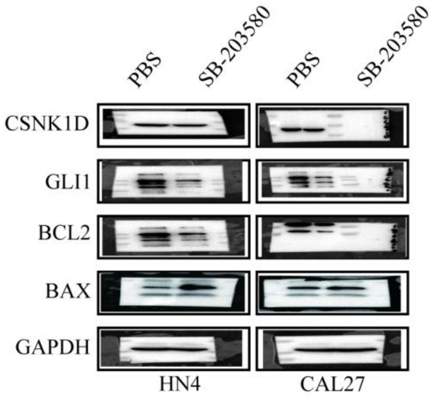

Supplement: Supplementary file 3 — Full original pictures [file 41419_2025_8276_MOESM3_ESM.pdf]
